# Supplementary material for: Synthesis and Biological Activity of N-acyl Anabasine and Cytisine Derivatives with Adamantane, Pyridine and 1,2-Azole Fragments
Source: Molecules. 2022 Oct 31;27(21):7387. doi: 10.3390/molecules27217387 (PMC9656753; doi:10.3390/molecules27217387)
Supplement: Supplementary file 1 [file molecules-27-07387-s001.zip › MS.pdf]

Qualitative Analysis Report

1a

**Data Filename**	KH-2_01.d	**Sample Name**	KH-2
**Sample Type**	Sample	**Position**	Vial 2
**Instrument Name**	Instrument 1	**User Name**	
**Acq Method**	All_2021_kol 1-6.m	**Acquired Time**	7/7/2021 10:53:54 AM
**IRM Calibration Status**	Not Applicable	**DA Method**	ChromPeakSurvey-Default.m
**Comment**			

**Sample Group**		**Info.**	
**Stream Name**	LC 1	**Acquisition SW Version**	6400 Series Triple Quadrupole 10.0 (127)

User Chromatograms

Fragmentor Voltage 135 **Collision Energy** 0 **Ionization Mode** ESI

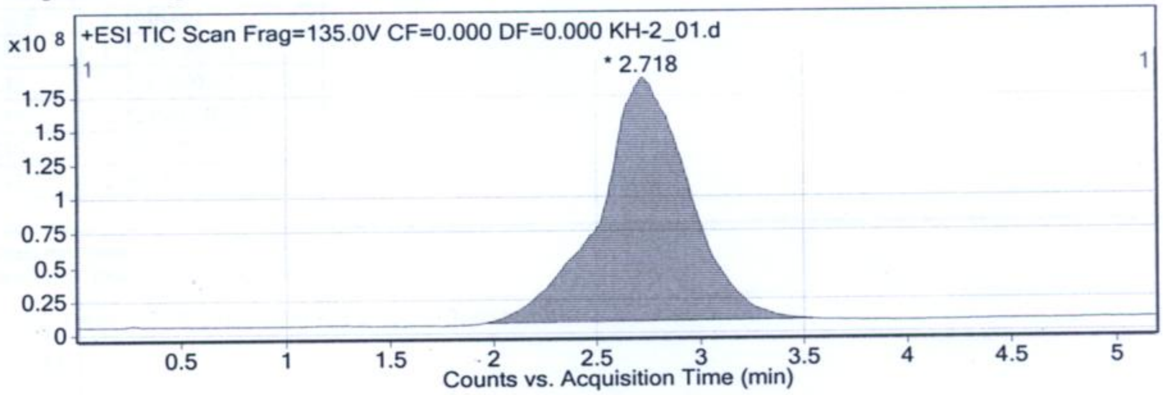

Integration Peak List

Peak	Start	RT	End	Height	Area	Area %
1	1,96	2,718	3,561	179040492	5424107700	100

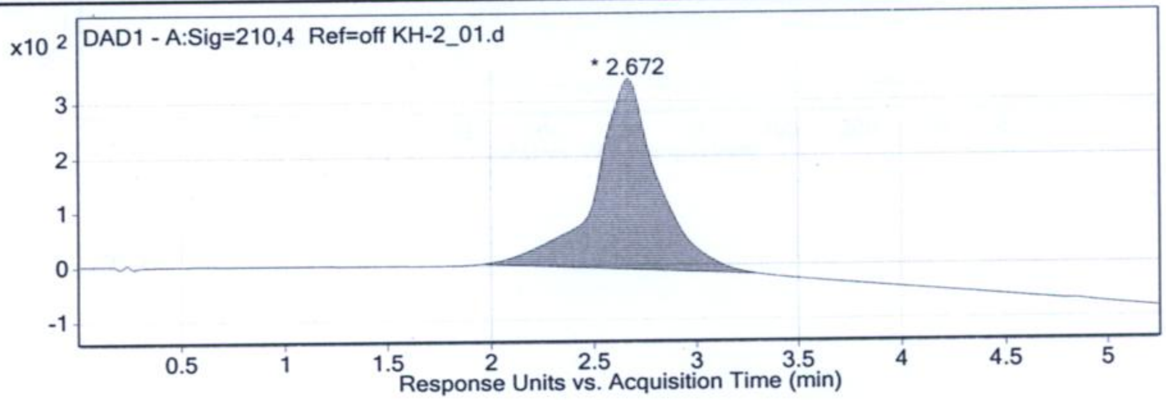

Integration Peak List

Peak	Start	RT	End	Height	Area	Area %
1	1,965	2,672	3,298	349,34	7478,49	100

User Spectra

**Spectrum Source**	**Fragmentor Voltage**	**Collision Energy**	**Ionization Mode**
Peak (1) in "+ TIC Scan"	135	0	ESI

Qualitative Analysis Report

1a

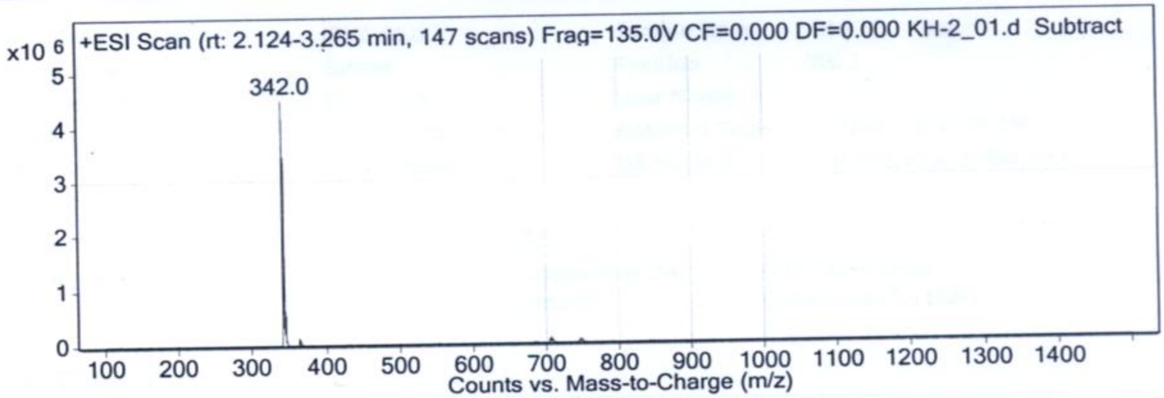

Peak List

m/z	z	Abund
342	1	4483982.5
343	1	779470.44
344	1	3454008.75
345	1	511992.94
346	1	627611

Spectrum Source
Peak (1) in "DAD1 - A:Sig=210,4 Ref=off"

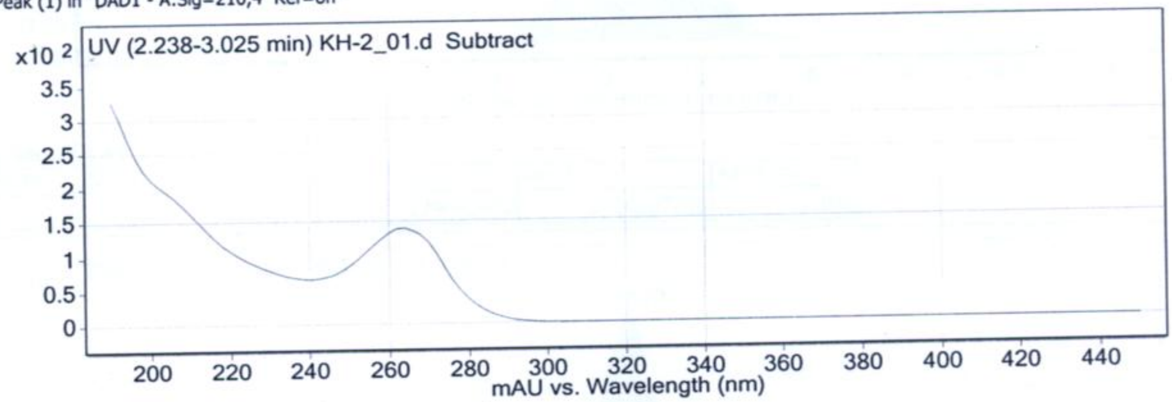

--- End Of Report ---

Qualitative Analysis Report

1b

**Data Filename**	KH-7_01.d	**Sample Name**	KH-7
**Sample Type**	Sample	**Position**	Vial 2
**Instrument Name**	Instrument 1	**User Name**	
**Acq Method**	All_2021_kol 1-6.m	**Acquired Time**	7/7/2021 11:50:12 AM
**IRM Calibration Status**	Not Applicable	**DA Method**	ChromPeakSurvey-Default.m
**Comment**			

Sample Group

Stream Name LC 1

Info.

Acquisition SW Version 6400 Series Triple Quadrupole 10.0 (127)

User Chromatograms

Fragmentor Voltage 135 **Collision Energy** 0 **Ionization Mode** ESI

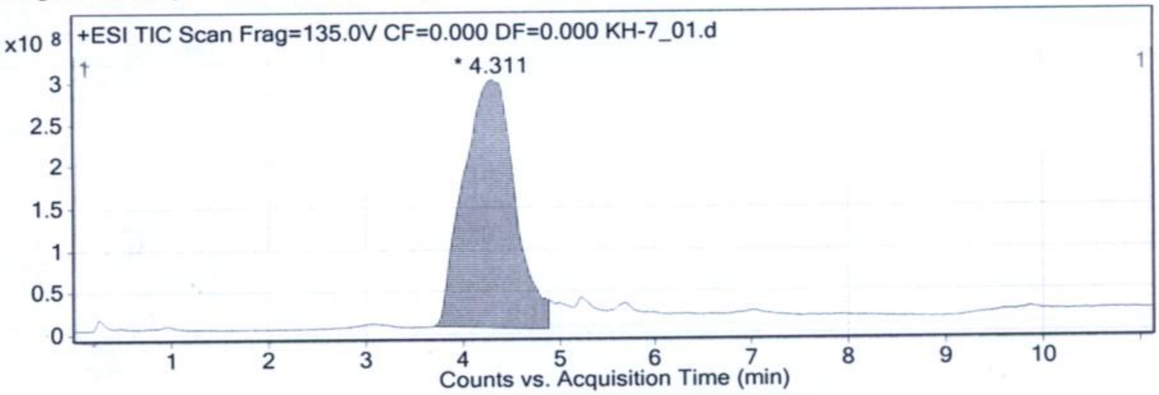

Integration Peak List

Peak	Start	RT	End	Height	Area	Area %
1	3,702	4,311	4,889	296137102	10852315410	100

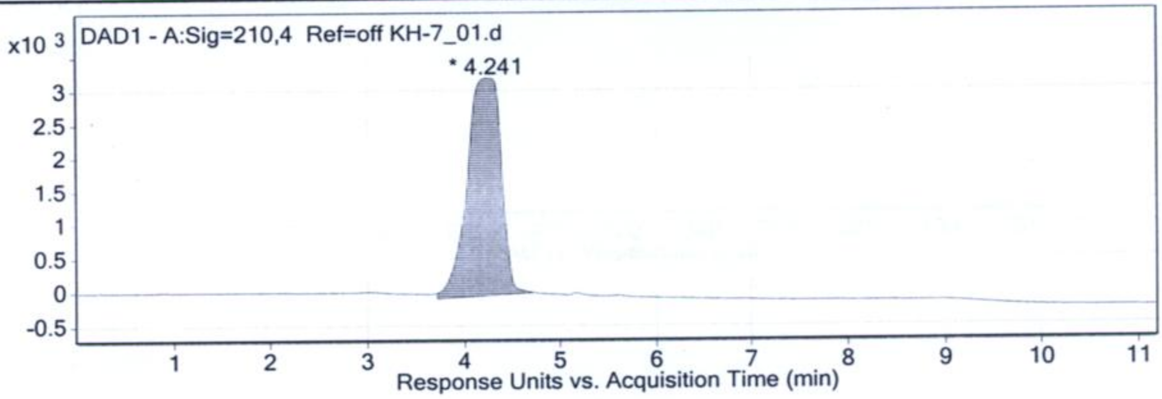

Integration Peak List

Peak	Start	RT	End	Height	Area	Area %
1	3,721	4,241	4,714	3227,47	79221,19	100

User Spectra

Spectrum Source	Fragmentor Voltage	Collision Energy	Ionization Mode
Peak (1) in "+ TIC Scan"	135	0	ESI

Qualitative Analysis Report

1b

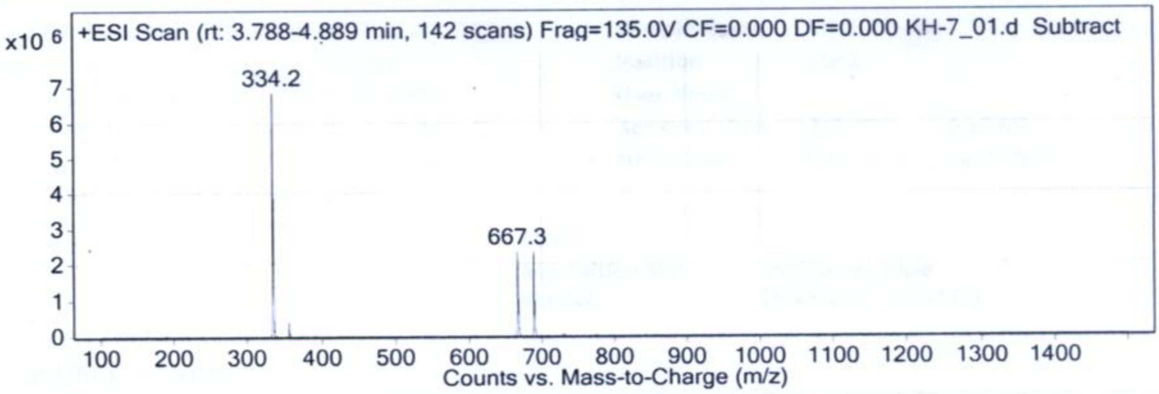

Peak List

m/z	z	Abund
334.2		6855443
335.2	1	3895998.5
336.2	1	399113.72
356.1		413714.38
667.3	1	2352337.25
668.3	1	1043561.69
689.3	1	2347706.25
690.3	1	963482.81

Spectrum Source

Peak (1) in "DAD1 - A:Sig=210,4 Ref=off"

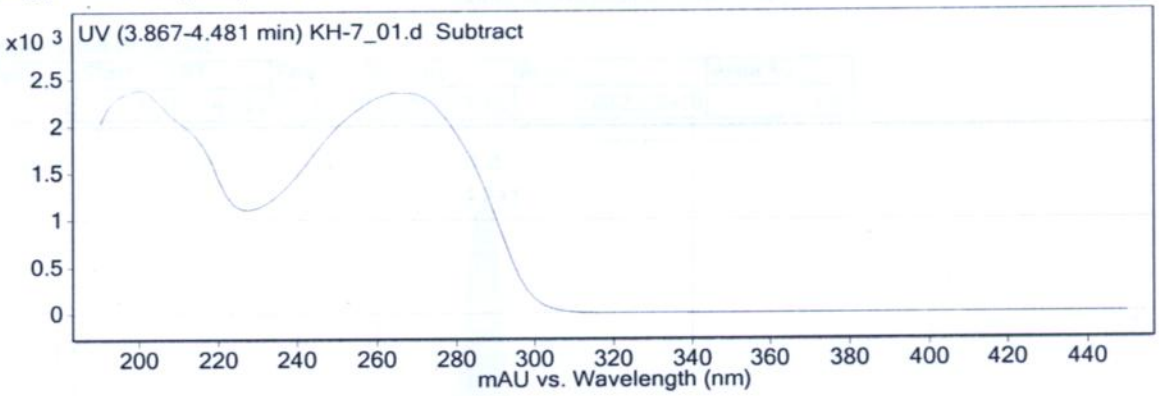

--- End Of Report ---

Qualitative Analysis Report

1c

**Data Filename**	KH-3_01.d	**Sample Name**	KH-3
**Sample Type**	Sample	**Position**	Vial 2
**Instrument Name**	Instrument 1	**User Name**	
**Acq Method**	All_2021_kol 1-6.m	**Acquired Time**	7/7/2021 11:03:42 AM
**IRM Calibration Status**	Not Applicable	**DA Method**	ChromPeakSurvey-Default.m
**Comment**			

**Sample Group**		**Info.**	
**Stream Name**	LC 1	**Acquisition SW Version**	6400 Series Triple Quadrupole 10.0 (127)

User Chromatograms

Fragmentor Voltage 135 **Collision Energy** 0 **Ionization Mode** ESI

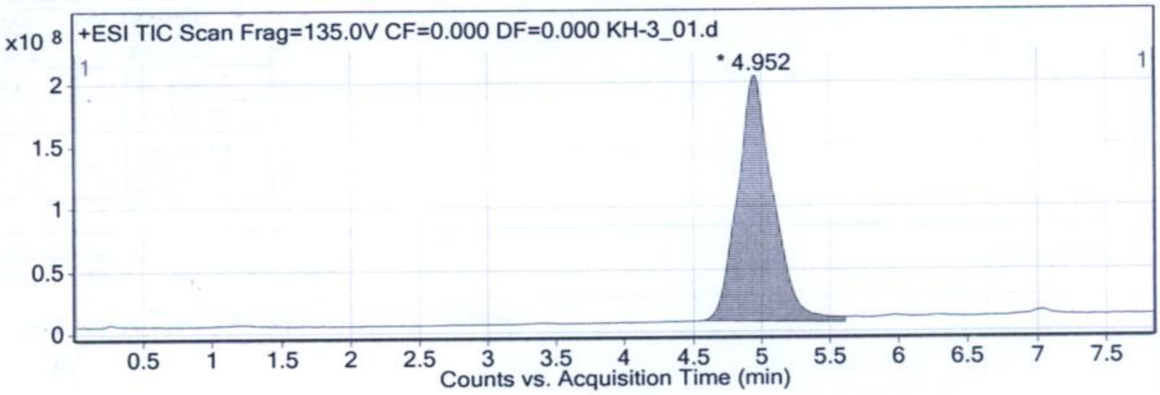

Integration Peak List

Peak	Start	RT	End	Height	Area	Area %
1	4,569	4,952	5,608	196857540	3582515160	100

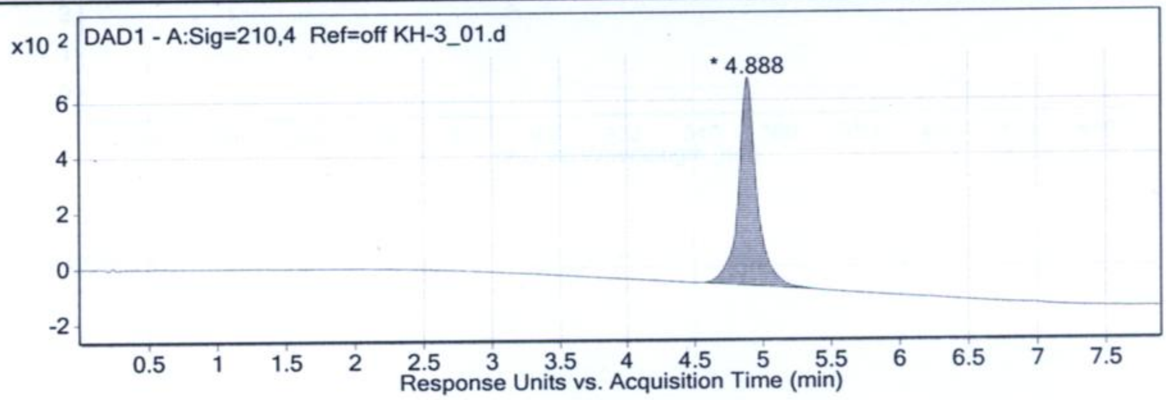

Integration Peak List

Peak	Start	RT	End	Height	Area	Area %
1	4,555	4,888	5,368	746,88	7067,83	100

User Spectra

Spectrum Source	Fragmentor Voltage	Collision Energy	Ionization Mode
Peak (1) in "+ TIC Scan"	135	0	ESI

Qualitative Analysis Report

1c

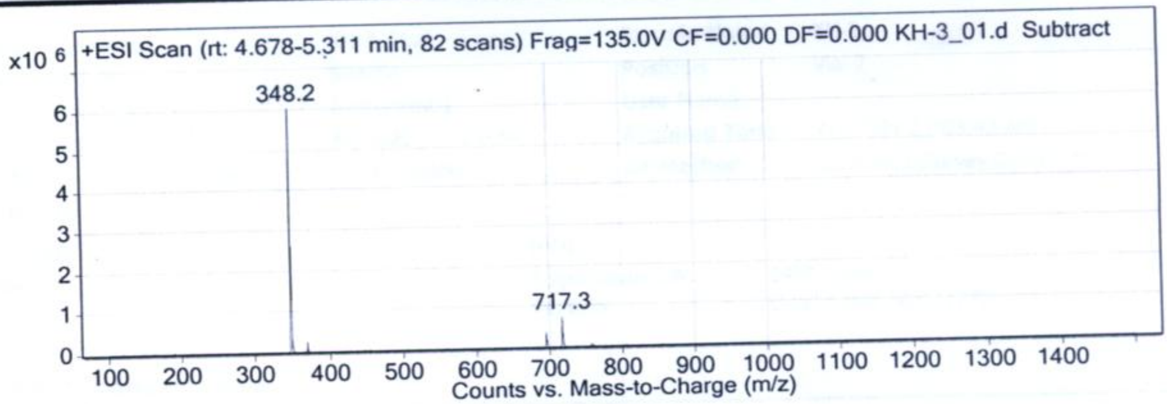

Peak List

m/z	z	Abund
348.2	1	6043613
349.2	1	2611541.5
695.3		364484.53
717.3	1	734109.44
718.3	1	333337.97

Spectrum Source
Peak (1) in "DAD1 - A:Sig=210,4 Ref=off"

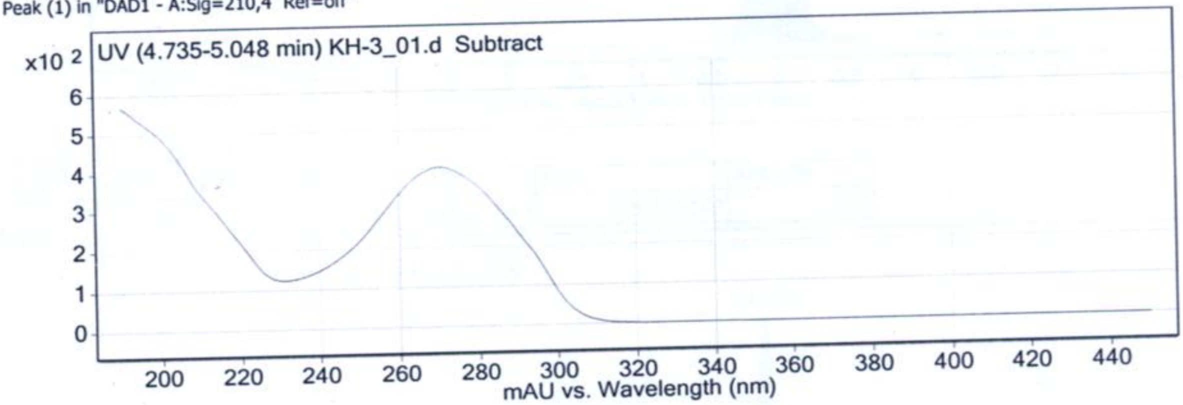

--- End Of Report ---

Qualitative Analysis Report

1d

**Data Filename**	KH-15_01.d	**Sample Name**	KH-15
**Sample Type**	Sample	**Position**	Vial 3
**Instrument Name**	Instrument 1	**User Name**	
**Acq Method**	All_2021_kol 1-2.m	**Acquired Time**	12/9/2021 11:18:06 AM
**IRM Calibration Status**	Not Applicable	**DA Method**	ChromPeakSurvey-Default.m
**Comment**			

**Sample Group**		**Info.**	
**Stream Name**	LC 1	**Acquisition SW Version**	6400 Series Triple Quadrupole 10.0 (127)

User Chromatograms

Fragmentor Voltage 135 **Collision Energy** 0 **Ionization Mode** ESI

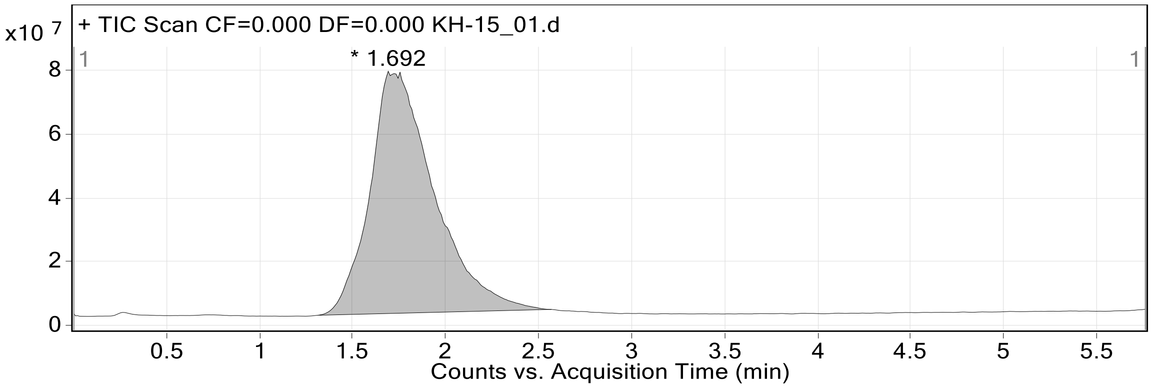

Integration Peak List

Peak	Start	RT	End	Height	Area	Area %
1	1,311	1,692	2,57	76132846	1827851718	100

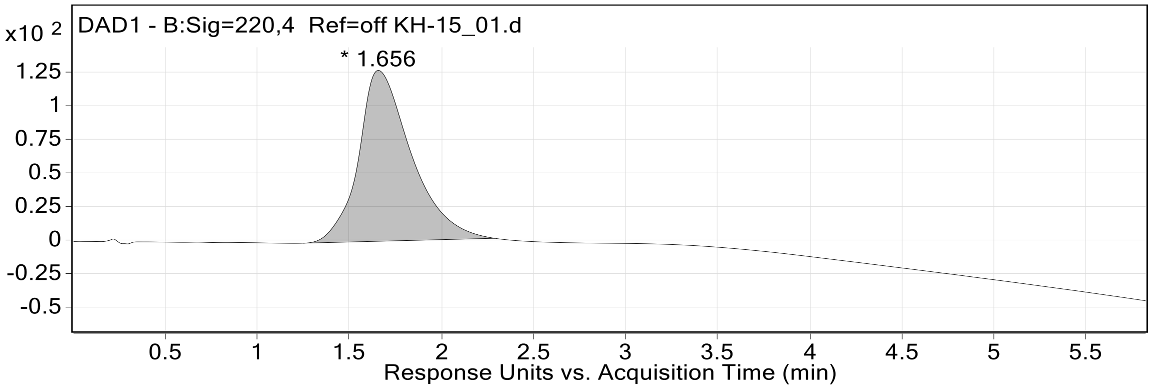

Integration Peak List

Peak	Start	RT	End	Height	Area	Area %
1	1,249	1,656	2,289	127,63	2551,63	100

User Spectra

Spectrum Source	Fragmentor Voltage	Collision Energy	Ionization Mode
Peak (1) in "+ TIC Scan"	135	0	ESI

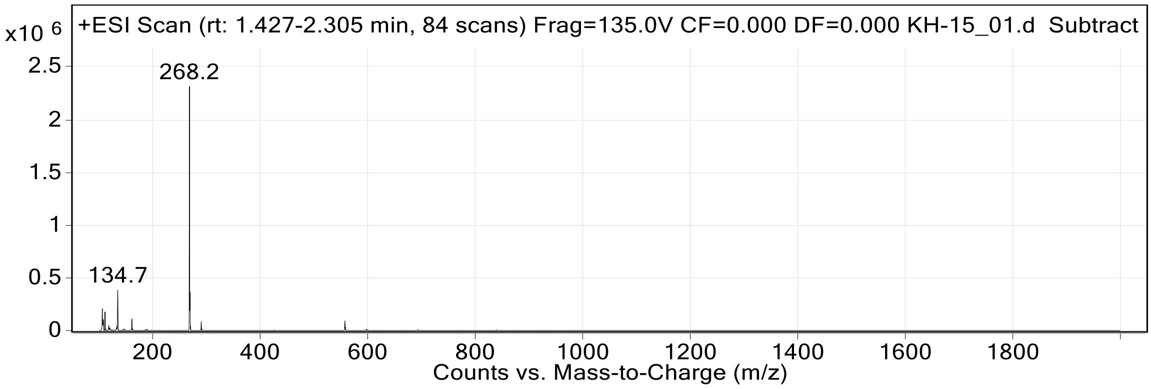

1d

Peak List

m/z	z	Abund
106.1		209516.75
111		176026.27
134.7	2	386140.56
268.2	1	2314920.75
269.2	1	366400.03

Spectrum Source
Peak (1) in "DAD1 - B:Sig=220,4 Ref=off"

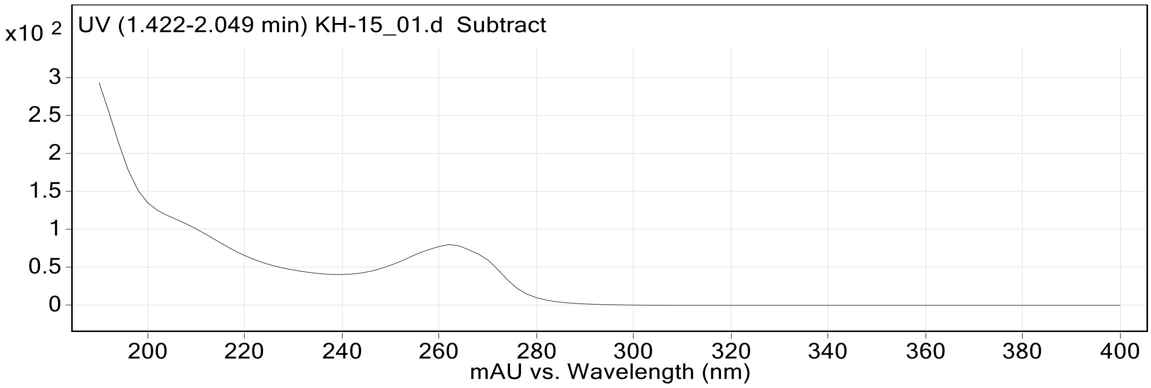

--- End Of Report ---

Qualitative Analysis Report

1e

**Data Filename**	KH-14_02.d	**Sample Name**	KH-14
**Sample Type**	Sample	**Position**	Vial 3
**Instrument Name**	Instrument 1	**User Name**	
**Acq Method**	All_2021_kol 1-2.m	**Acquired Time**	12/9/2021 10:45:01 AM
**IRM Calibration Status**	Not Applicable	**DA Method**	ChromPeakSurvey-Default.m
**Comment**			

**Sample Group**		**Info.**	
**Stream Name**	LC 1	**Acquisition SW Version**	6400 Series Triple Quadrupole 10.0 (127)

User Chromatograms

Fragmentor Voltage 135 **Collision Energy** 0 **Ionization Mode** ESI

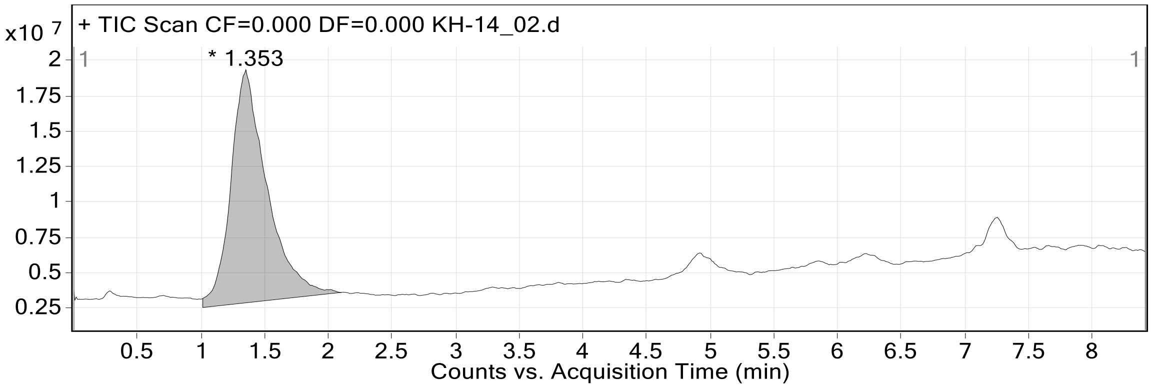

Integration Peak List

Peak	Start	RT	End	Height	Area	Area %
1	1,015	1,353	2,104	16491217	326122349	100

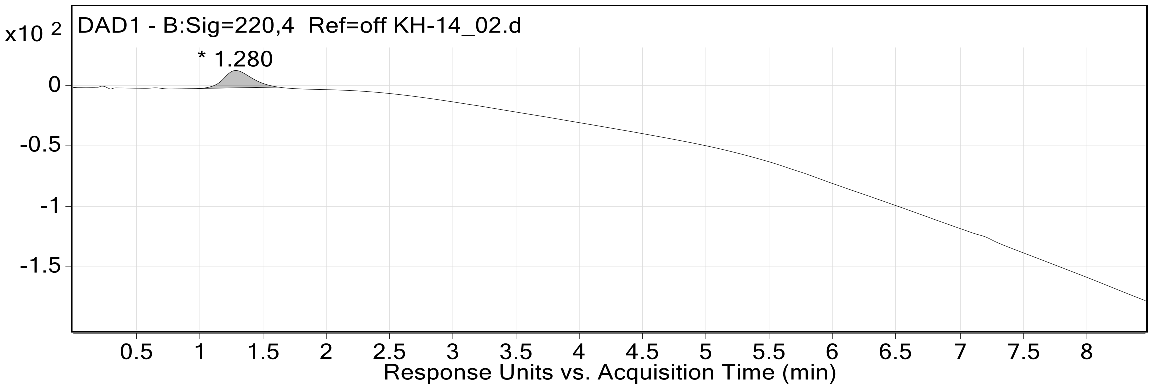

Integration Peak List

Peak	Start	RT	End	Height	Area	Area %
1	0,994	1,28	1,62	14,41	224,5	100

User Spectra

Spectrum Source	Fragmentor Voltage	Collision Energy	Ionization Mode
Peak (1) in "+ TIC Scan"	135	0	ESI

Qualitative Analysis Report

1e

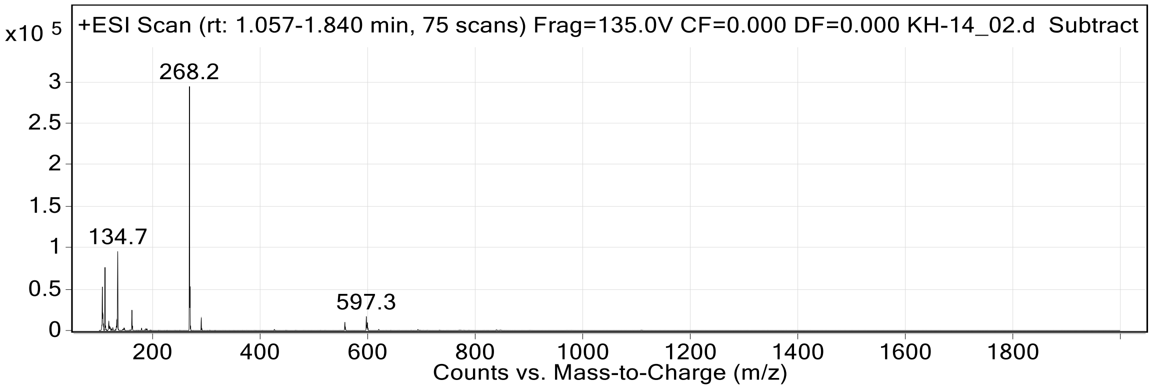

Peak List

m/z	z	Abund
106.1		52663.57
107.1		19463.6
111.1		76161.67
134.7	2	95331.52
161.1		24623.17
268.2	1	294176.53
269.2	1	53026.48
290.2		14820.42
597.3		16650.44

Spectrum Source

Peak (1) in "DAD1 - B:Sig=220,4 Ref=off"

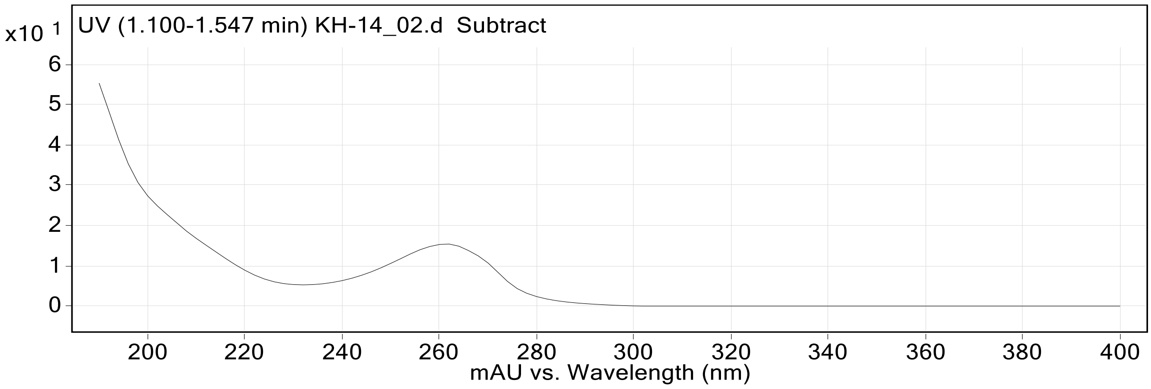

--- End Of Report ---

Qualitative Analysis Report

1f

**Data Filename**	KH-10_02.d	**Sample Name**	KH-10
**Sample Type**	Sample	**Position**	Vial 2
**Instrument Name**	Instrument 1	**User Name**	
**Acq Method**	All_2021_kol 1-6.m	**Acquired Time**	7/7/2021 12:53:56 PM
**IRM Calibration Status**	Not Applicable	**DA Method**	ChromPeakSurvey-Default.m
**Comment**			

Sample Group

Stream Name LC 1

Info.

Acquisition SW Version 6400 Series Triple Quadrupole 10.0 (127)

User Chromatograms

Fragmentor Voltage 135 **Collision Energy** 0 **Ionization Mode** ESI

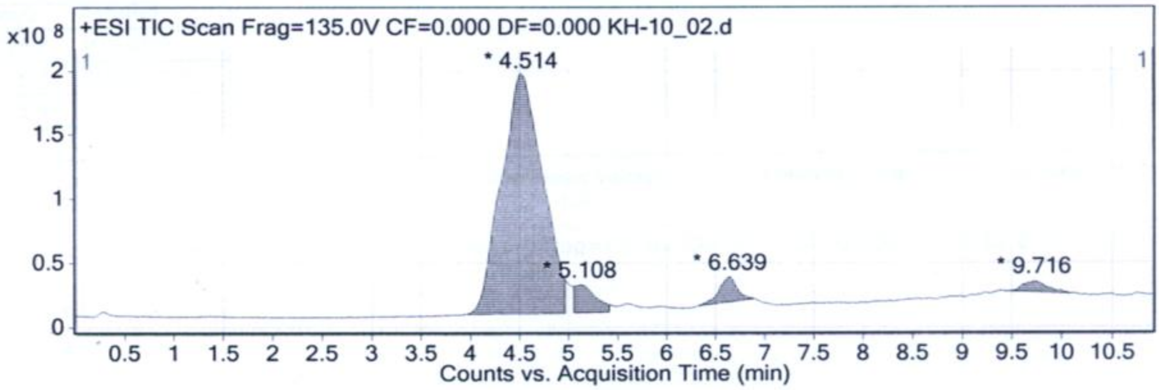

Integration Peak List

Peak	Start	RT	End	Height	Area	Area %
1	3,968	4,514	4,959	186829894	5411004220	100
2	5,045	5,108	5,413	21549467	316606693	5,85
3	6,319	6,639	6,889	19226173	248490324	4,59
4	9,466	9,716	10,107	8659998	139787732	2,58

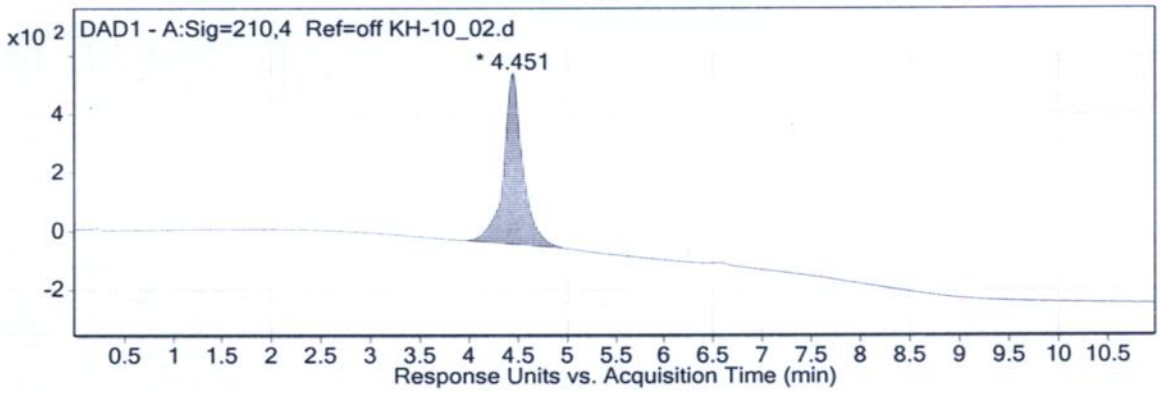

Integration Peak List

Peak	Start	RT	End	Height	Area	Area %
1	3,951	4,451	4,957	585,16	8014,72	100

User Spectra

Spectrum Source	Fragmentor Voltage	Collision Energy	Ionization Mode
Peak (1) in "+ TIC Scan"	135	0	ESI

1f

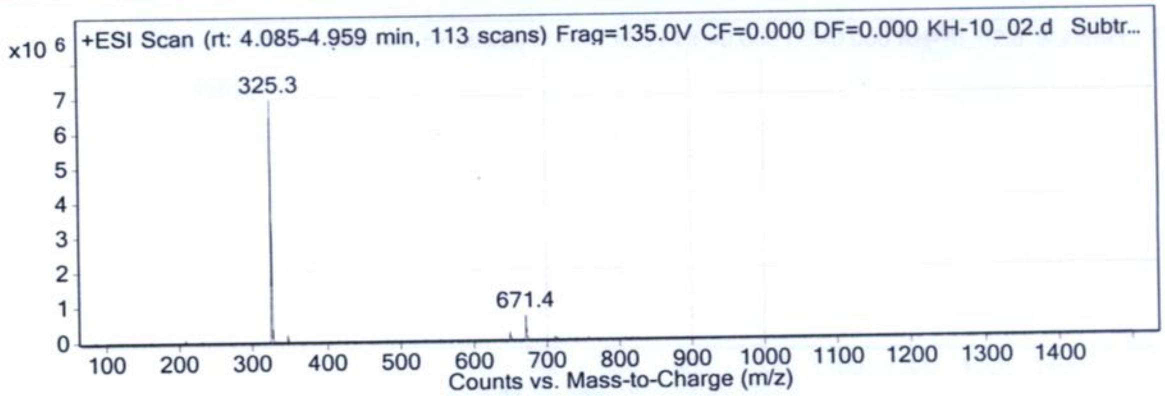

Peak List

m/z	Abund
325.3	6969857
326.2	3419790.5
671.4	694387.19

Spectrum Source	Fragmentor Voltage	Collision Energy	Ionization Mode
Peak (2) in "+ TIC Scan"	135	0	ESI

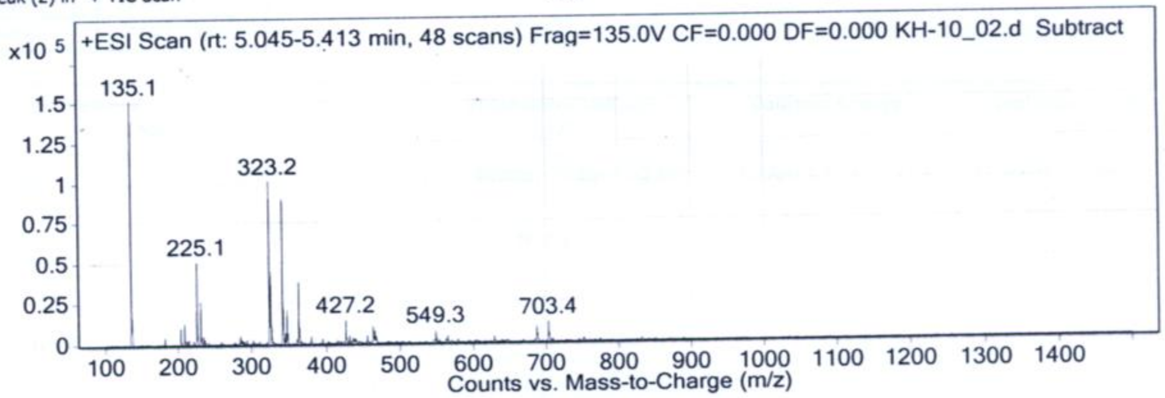

Peak List

m/z	z	Abund
135.1	1	151371.75
225.1		50977.1
230.2		26681.79
323.2		100920.01
324.3		24925.95
325.2	1	44495.69
341.2	1	89726.94
342.2	1	21968.41
347.2		21161.31
363.2	1	38406.2

Spectrum Source	Fragmentor Voltage	Collision Energy	Ionization Mode
Peak (3) in "+ TIC Scan"	135	0	ESI

1f

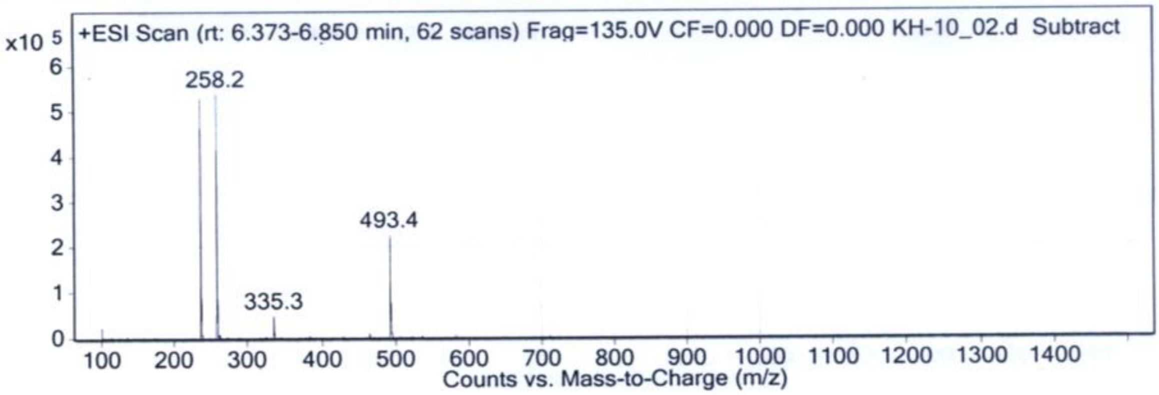

Peak List

m/z	z	Abund
236.2	1	530022.06
237.2	1	86117.61
258.2	1	539195.81
259.2	1	86259.48
335.3		48151.04
493.4	1	226153.64
494.4	1	76522.74

Spectrum Source	Fragmentor Voltage	Collision Energy	Ionization Mode
Peak (4) in "+ TIC Scan"	135	0	ESI

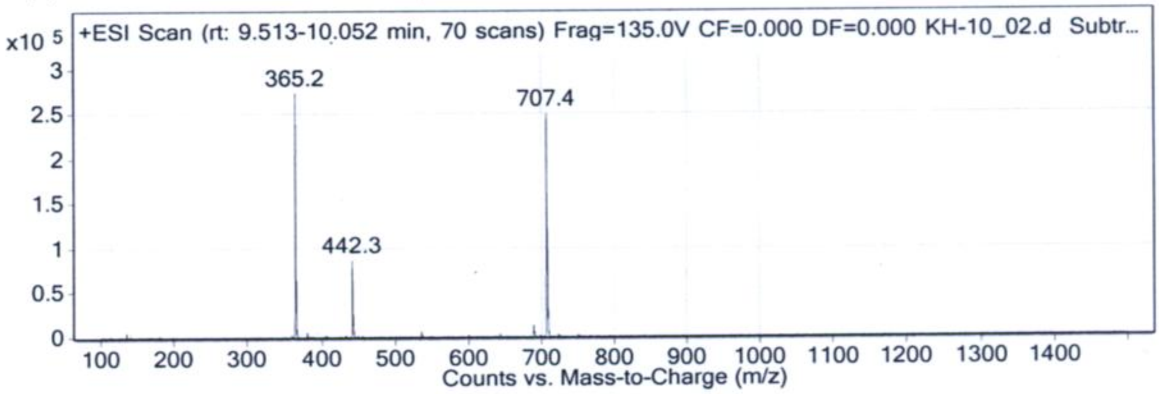

Peak List

m/z	z	Abund
365.2	1	273867.41
366.2	1	65232.78
442.3	1	87042.62
443.3	1	25555.42
707.4	1	250704.56
708.4	1	121372.04
709.4	1	31058.05

Spectrum Source
Peak (1) in "DAD1 - A:Sig=210,4 Ref=off"

Qualitative Analysis Report

2a

**Data Filename**	KH-2--Me-i_01.d	**Sample Name**	KH-2--Me-i
**Sample Type**	Sample	**Position**	Vial 2
**Instrument Name**	Instrument 1	**User Name**	
**Acq Method**	All_2021_kol 1-6.m	**Acquired Time**	7/7/2021 2:02:00 PM
**IRM Calibration Status**	Not Applicable	**DA Method**	ChromPeakSurvey-Default.m
**Comment**			

Sample Group

Stream Name LC 1

Info.

Acquisition SW Version 6400 Series Triple Quadrupole 10.0 (127)

User Chromatograms

Fragmentor Voltage 135 **Collision Energy** 0 **Ionization Mode** ESI

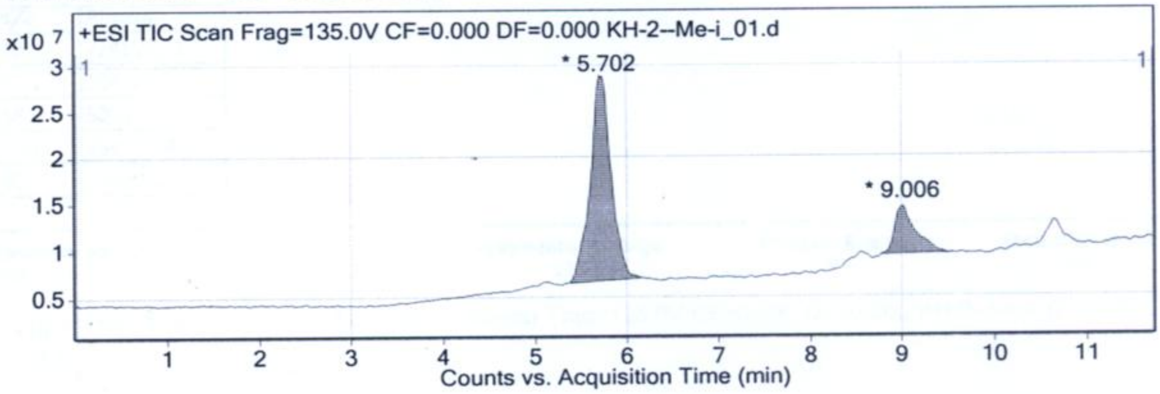

Integration Peak List

Peak	Start	RT	End	Height	Area	Area %
1	5,334	5,702	6,162	22168066	349313956	100
2	8,771	9,006	9,56	5141560	79236691	22,68

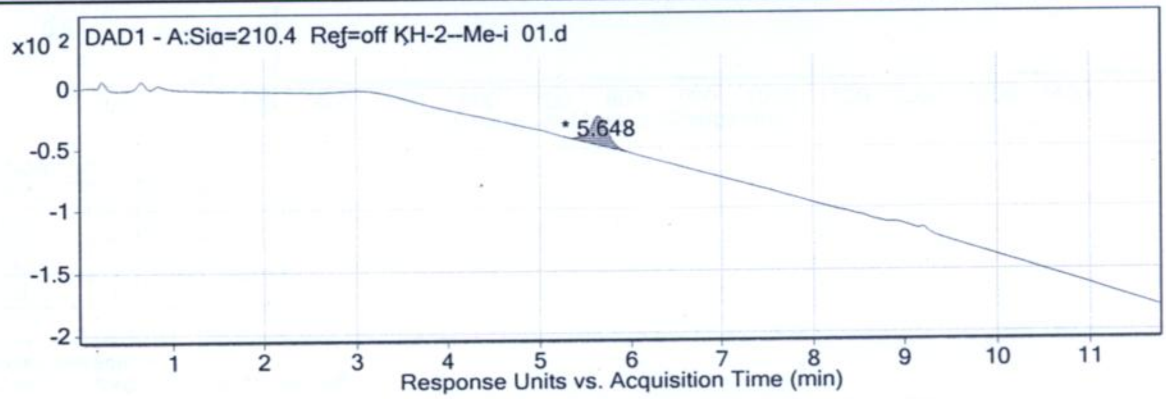

Integration Peak List

Peak	Start	RT	End	Height	Area	Area %
1	5,261	5,648	5,968	23,77	316,46	100

User Spectra

Spectrum Source	Fragmentor Voltage	Collision Energy	Ionization Mode
Peak (1) in "+ TIC Scan"	135	0	ESI

2a

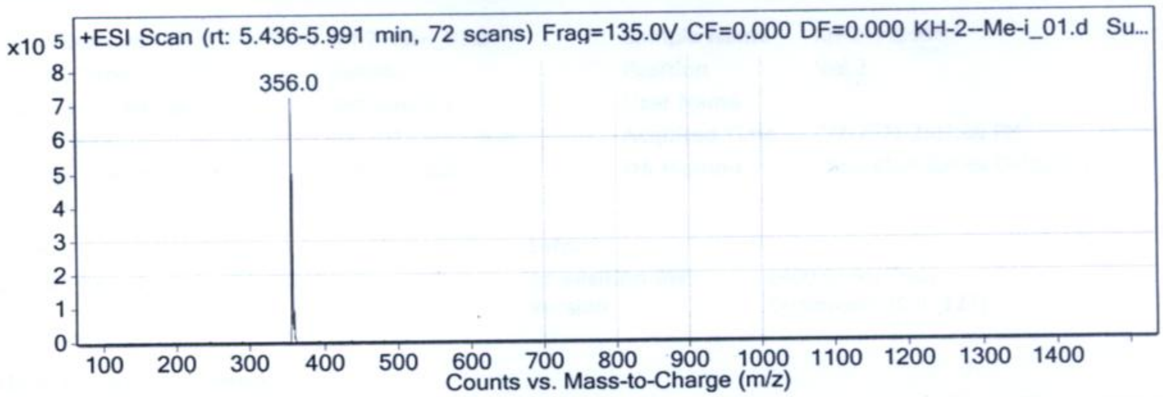

Peak List

m/z	Abund
356	724197.31
357.1	124073.7
358	500415.69
359.1	85457.09
360	96420.07

Spectrum Source	Fragmentor Voltage	Collision Energy	Ionization Mode
Peak (2) in "+ TIC Scan"	135	0	ESI

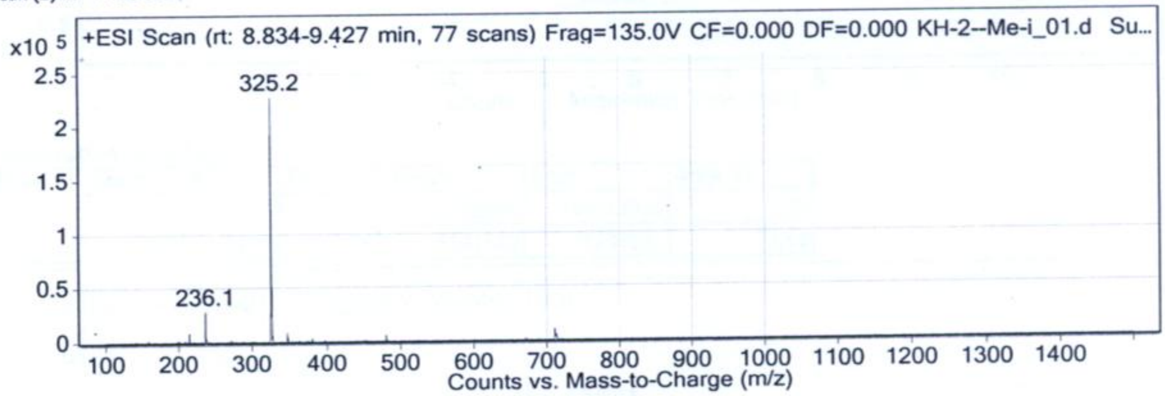

Peak List

m/z	z	Abund
236.1		28168.8
325.2	1	228032.73
326.2	1	51696.23

Spectrum Source
Peak (1) in "DAD1 - A:Sig=210,4 Ref=off"

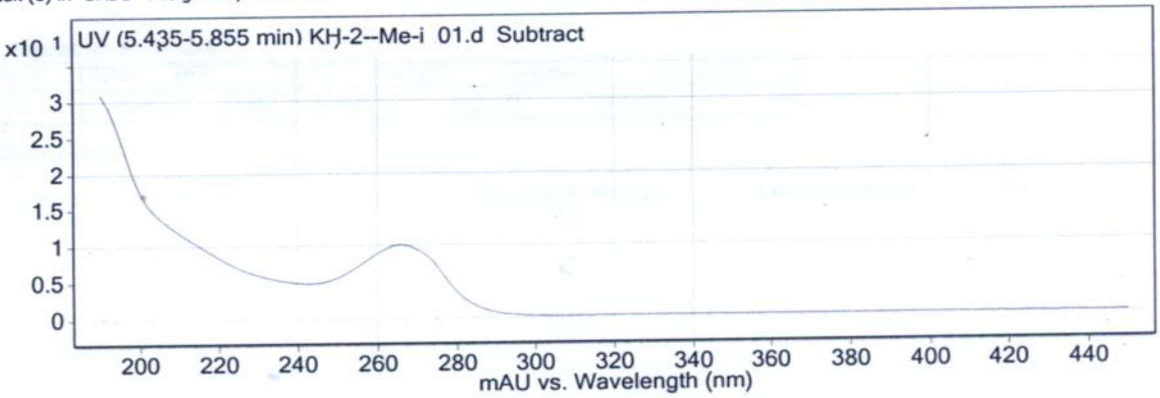

--- End Of Report ---

Qualitative Analysis Report

2b

**Data Filename**	KH-7--Me-i_01.d	**Sample Name**	KH-7-Me-i
**Sample Type**	Sample	**Position**	Vial 2
**Instrument Name**	Instrument 1	**User Name**	
**Acq Method**	All_2021_kol 1-6.m	**Acquired Time**	7/7/2021 2:33:54 PM
**IRM Calibration Status**	Not Applicable	**DA Method**	ChromPeakSurvey-Default.m
**Comment**			

**Sample Group**		**Info.**	
**Stream Name**	LC 1	**Acquisition SW Version**	6400 Series Triple Quadrupole 10.0 (127)

User Chromatograms

Fragmentor Voltage 135 **Collision Energy** 0 **Ionization Mode** ESI

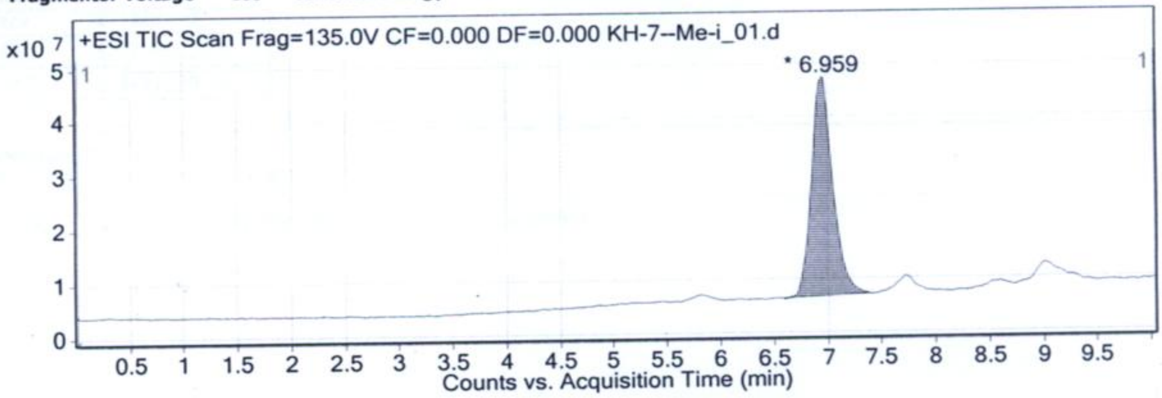

Integration Peak List

Peak	Start	RT	End	Height	Area	Area %
1	6,576	6,959	7,389	40689006	555865563	100

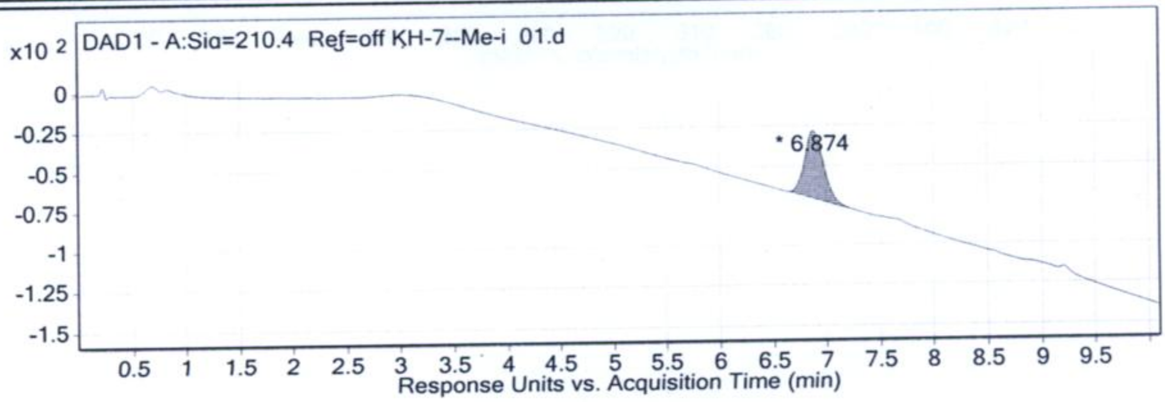

Integration Peak List

Peak	Start	RT	End	Height	Area	Area %
1	6,668	6,874	7,234	42,42	513,74	100

User Spectra

Spectrum Source	Fragmentor Voltage	Collision Energy	Ionization Mode
Peak (1) in "+ TIC Scan"	135	0	ESI

Qualitative Analysis Report

2b

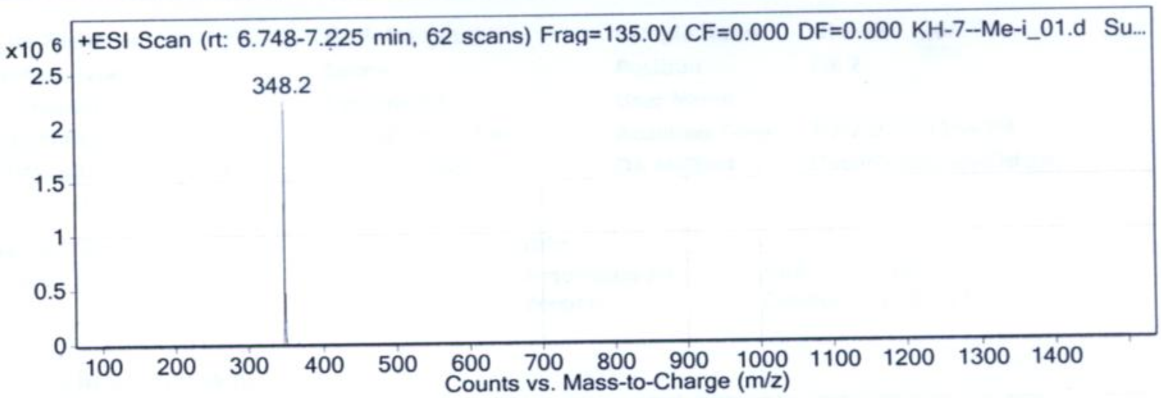

Peak List

m/z	z	Abund
348.2	1	2260211.5
349.2	1	472039.06

Spectrum Source
Peak (1) in "DAD1 - A:Sig=210,4 Ref=off"

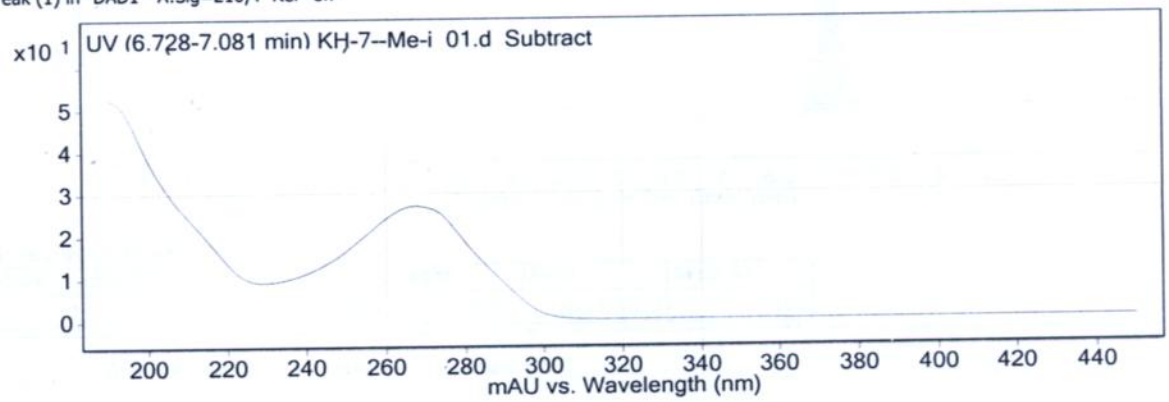

--- End Of Report ---

Qualitative Analysis Report

2c

**Data Filename**	KH-3--Me-i_01.d	**Sample Name**	KH-3-Me-i
**Sample Type**	Sample	**Position**	Vial 2
**Instrument Name**	Instrument 1	**User Name**	
**Acq Method**	All_2021_kol 1-6.m	**Acquired Time**	7/7/2021 2:18:29 PM
**IRM Calibration Status**	Not Applicable	**DA Method**	ChromPeakSurvey-Default.m
**Comment**			

Sample Group

Stream Name LC 1

Info.

Acquisition SW Version 6400 Series Triple Quadrupole 10.0 (127)

User Chromatograms

Fragmentor Voltage 135 **Collision Energy** 0 **Ionization Mode** ESI

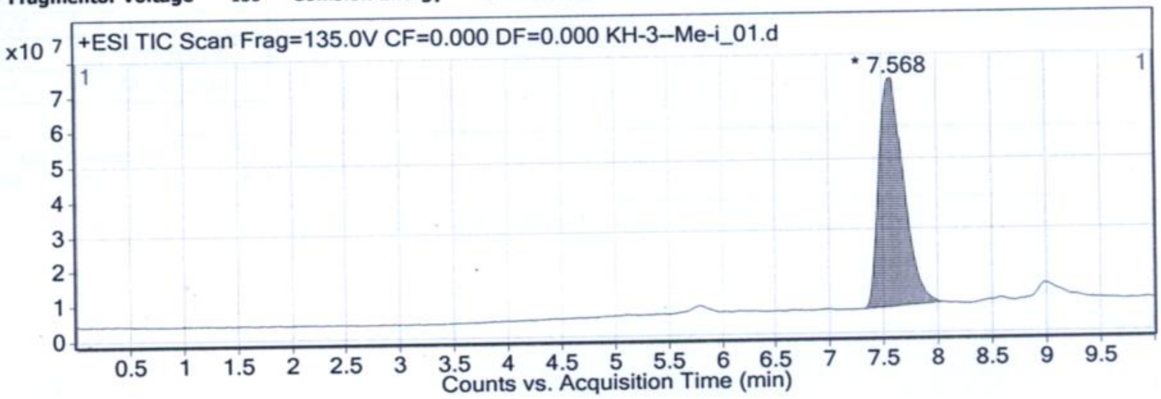

Integration Peak List

Peak	Start	RT	End	Height	Area	Area %
1	7,318	7,568	8,045	65846696	1043858244	100

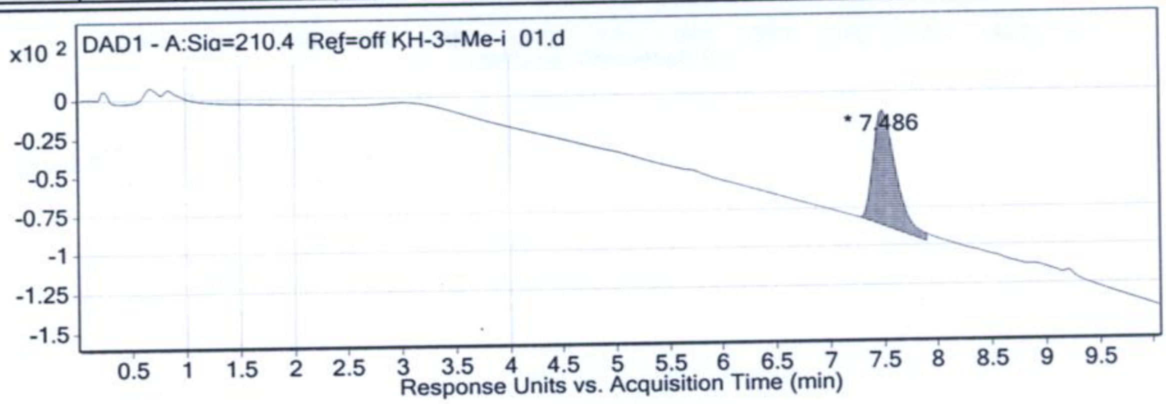

Integration Peak List

Peak	Start	RT	End	Height	Area	Area %
1	7,273	7,486	7,893	74,09	1073,79	100

User Spectra

Spectrum Source	Fragmentor Voltage	Collision Energy	Ionization Mode
Peak (1) in "+ TIC Scan"	135	0	ESI

Qualitative Analysis Report

2c

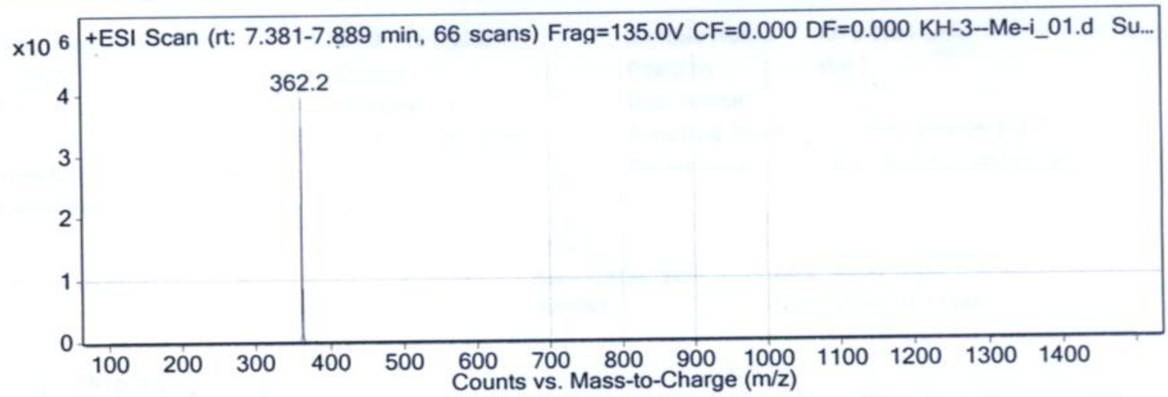

Peak List

m/z	z	Abund
362.2	1	3955921.5
363.2	1	870050.88

Spectrum Source
Peak (1) in "DAD1 - A:Sig=210,4 Ref=off"

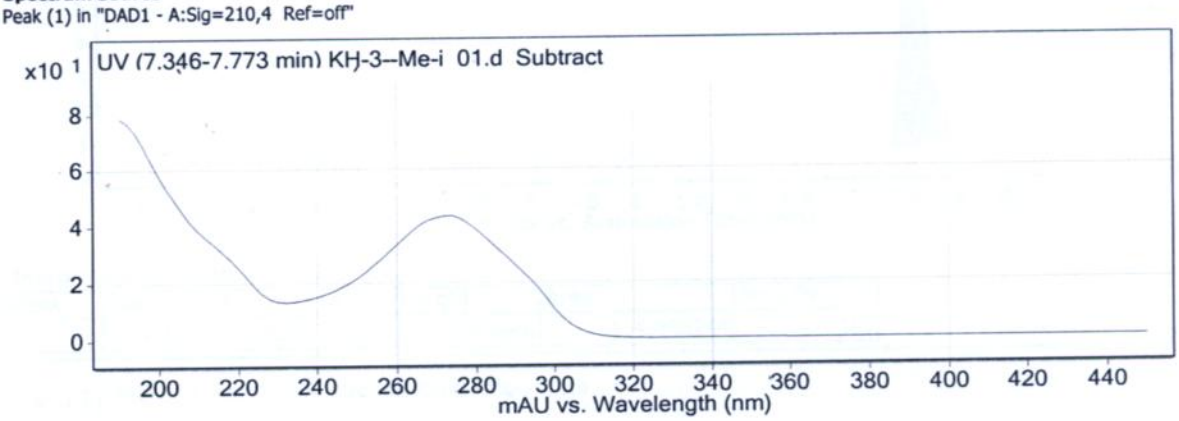

--- End Of Report ---

Qualitative Analysis Report

**Data Filename**	KH-15 MeI_01.d	**Sample Name**	KH-15 MeI
**Sample Type**	Sample	**Position**	Vial 3
**Instrument Name**	Instrument 1	**User Name**	
**Acq Method**	All_2021_kol 1-2.m	**Acquired Time**	12/9/2021 11:28:57 AM
**IRM Calibration Status**	Not Applicable	**DA Method**	ChromPeakSurvey-Default.m
**Comment**			

2d

**Sample Group**		**Info.**	
**Stream Name**	LC 1	**Acquisition SW Version**	6400 Series Triple Quadrupole 10.0 (127)

User Chromatograms

Fragmentor Voltage 135 **Collision Energy** 0 **Ionization Mode** ESI

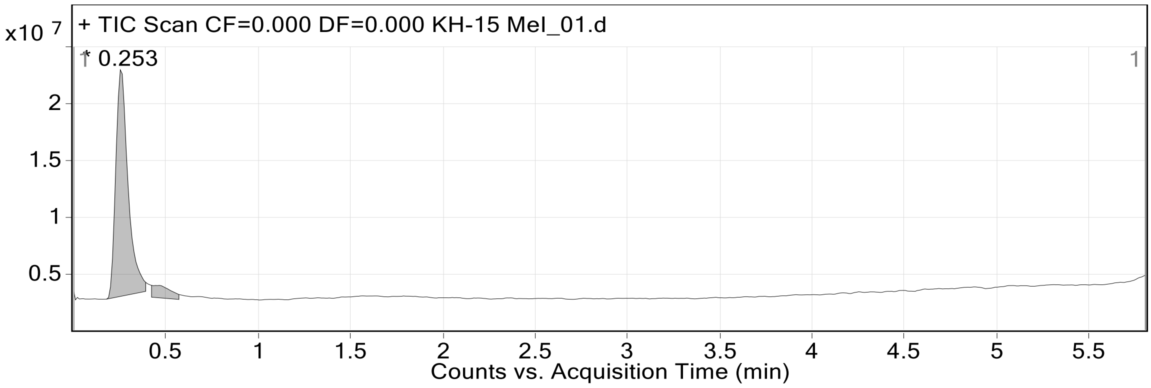

Integration Peak List

Peak	Start	RT	End	Height	Area	Area %
1	0,179	0,253	0,391	19910677	93371174	100
2	0,423	0,423	0,571	1037298	7379516	7,9

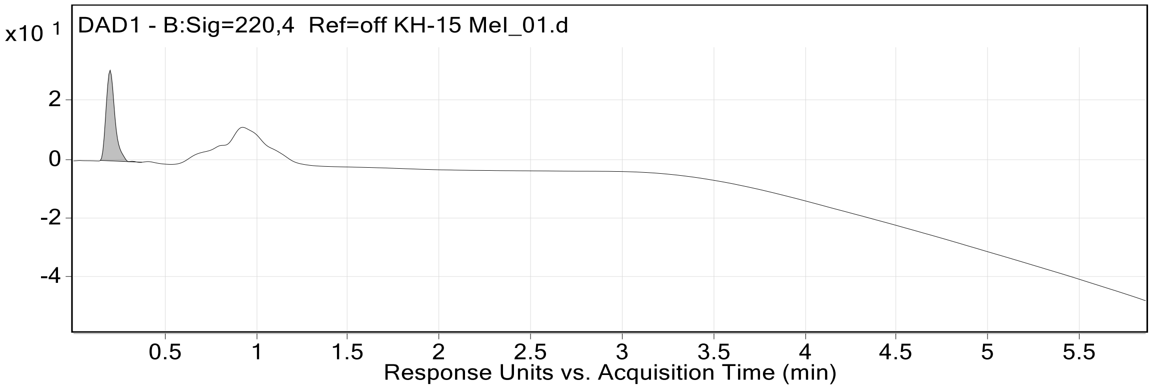

Integration Peak List

Peak	Start	RT	End	Height	Area	Area %
1	0,148	0,202	0,375	30,82	103,38	100

User Spectra

Spectrum Source	Fragmentor Voltage	Collision Energy	Ionization Mode
Peak (1) in "+ TIC Scan"	135	0	ESI

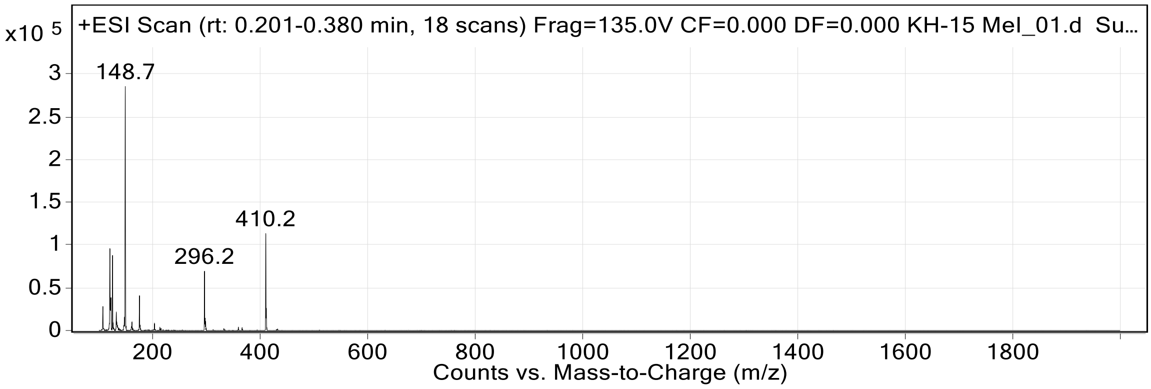

2d

Peak List

m/z	z	Abund
107.1		28257.09
120.1		95891.42
121		30142.12
122.1		38542.47
125.1		88000.17
148.7	2	285469.94
175.2		40868.9
296.2	1	69469.74
410.2		113481.53
411.3		25604.78

Spectrum Source	Fragmentor Voltage	Collision Energy	Ionization Mode
Peak (2) in "+ TIC Scan"	135	0	ESI

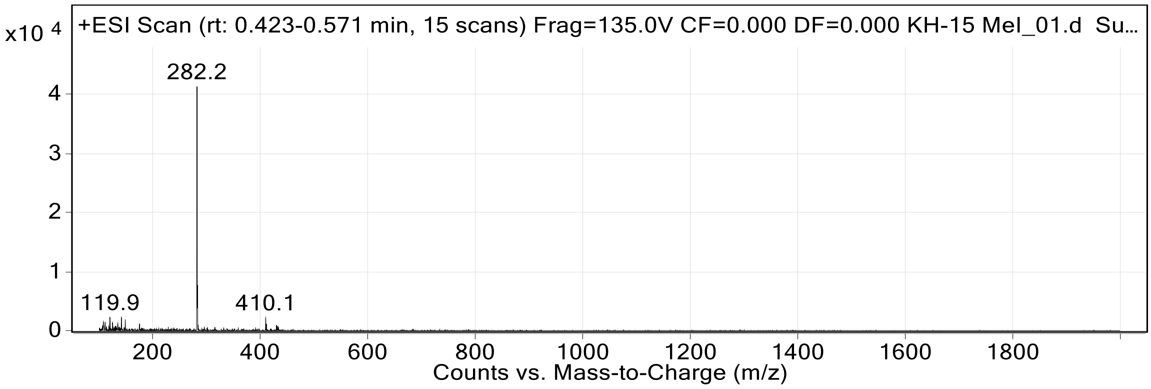

Peak List

m/z	z	Abund
119.9		2238.09
120.3		2179.19
141.5	2	2191.59
282.2	1	41304.45
283.2	1	7687.95
410.1		2190.64

Spectrum Source
Peak (1) in "DAD1 - B:Sig=220,4 Ref=off"

Qualitative Analysis Report

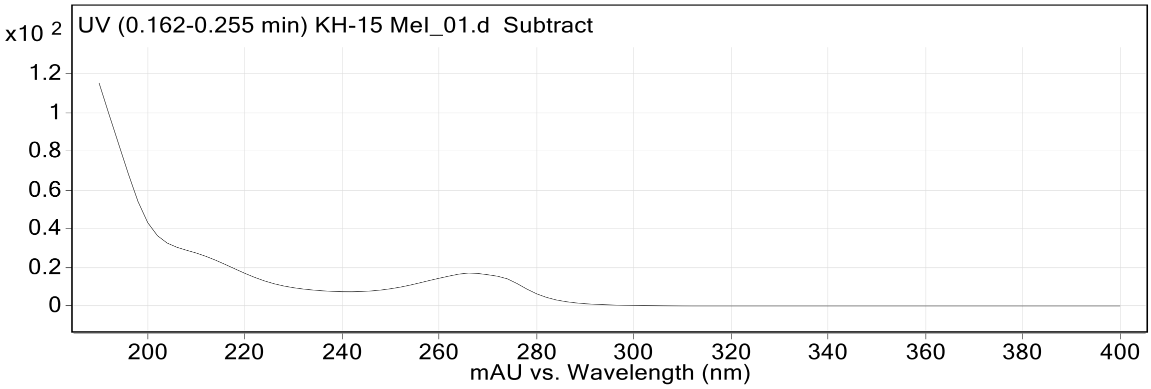

2d

--- End Of Report ---

Qualitative Analysis Report

2e

**Data Filename**	KH-14 MeI_02.d	**Sample Name**	KH-14 MeI
**Sample Type**	Sample	**Position**	Vial 3
**Instrument Name**	Instrument 1	**User Name**	
**Acq Method**	All_2021_kol 1-2.m	**Acquired Time**	12/9/2021 11:07:47 AM
**IRM Calibration Status**	Not Applicable	**DA Method**	ChromPeakSurvey-Default.m
**Comment**			

**Sample Group**		**Info.**	
**Stream Name**	LC 1	**Acquisition SW Version**	6400 Series Triple Quadrupole 10.0 (127)

User Chromatograms

Fragmentor Voltage 135 **Collision Energy** 0 **Ionization Mode** ESI

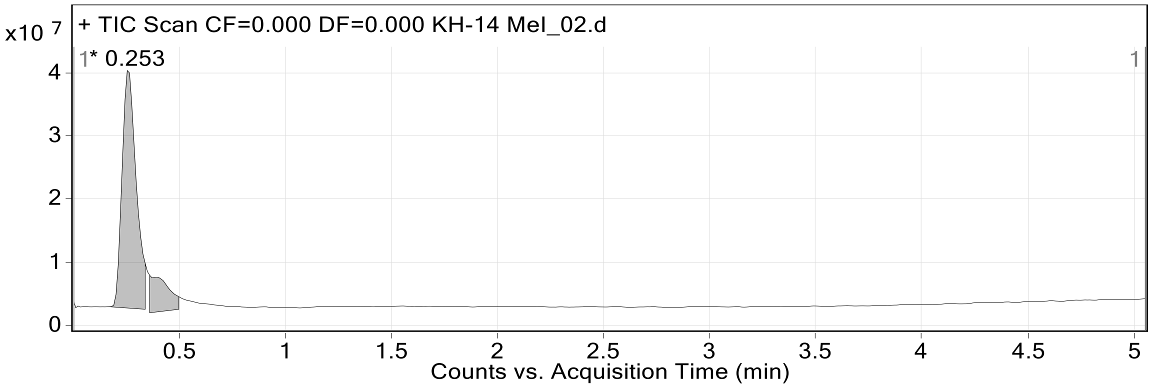

Integration Peak List

Peak	Start	RT	End	Height	Area	Area %
1	0,169	0,253	0,338	37608860	176184297	100
2	0,359	0,359	0,497	5942944	33679653	19,12

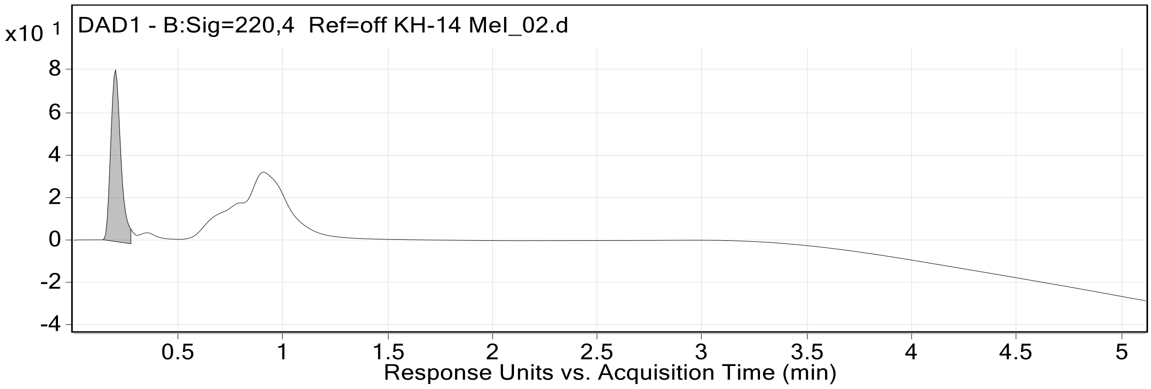

Integration Peak List

Peak	Start	RT	End	Height	Area	Area %
1	0,142	0,202	0,276	80,44	270,81	100

User Spectra

Spectrum Source	Fragmentor Voltage	Collision Energy	Ionization Mode
Peak (1) in "+ TIC Scan"	135	0	ESI

2e

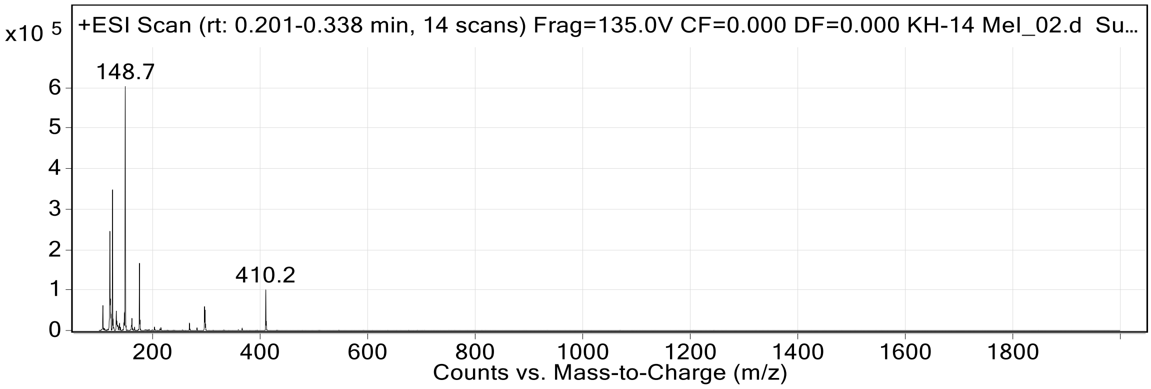

Peak List

m/z	z	Abund
107.1		62145.61
120.1		245466.17
121.1		78604.02
125.1		348038.06
132.1		48494.52
148.7	2	603012.88
175.2		166433.56
296.2		59401.49
297.3		50998.73
410.2		100753.29

Spectrum Source	Fragmentor Voltage	Collision Energy	Ionization Mode
Peak (2) in "+ TIC Scan"	135	0	ESI

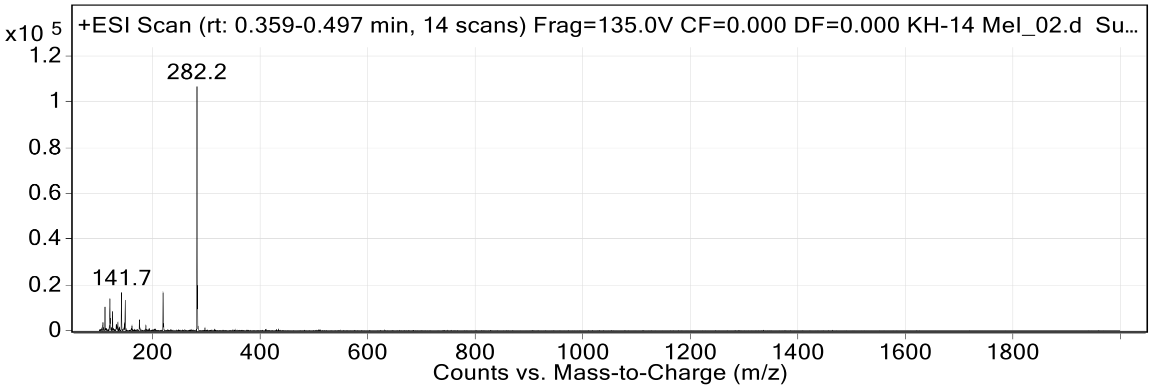

Peak List

m/z	z	Abund
111		10374.39
120.1		13932.16
121.1		5476.77
125.1		8341.93
141.7	2	16616.88
148.7	2	13239.06
219.1		16406.04
282.2	1	106663.66
283.2	1	19729.4

Spectrum Source
Peak (1) in "DAD1 - B:Sig=220,4 Ref=off"

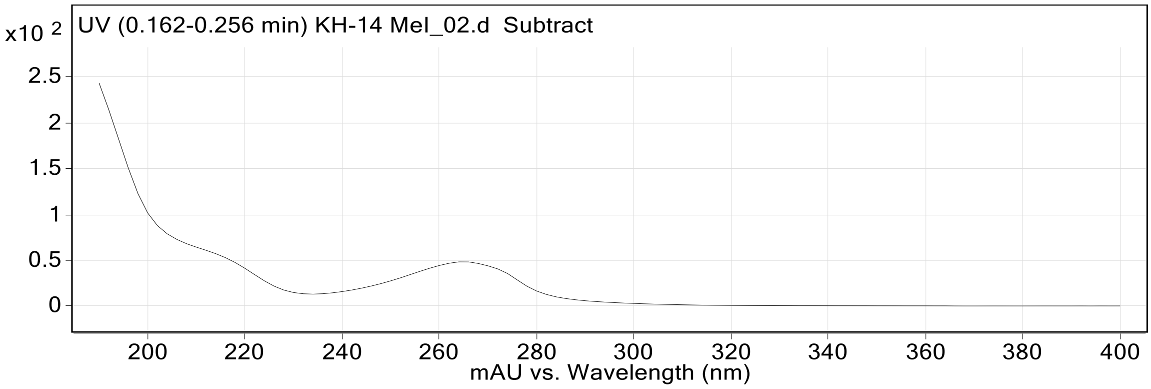

2e

--- End Of Report ---

Qualitative Analysis Report

3a

**Data Filename**	KH-4_01.d	**Sample Name**	KH-4
**Sample Type**	Sample	**Position**	Vial 2
**Instrument Name**	Instrument 1	**User Name**	
**Acq Method**	All_2021_kol 1-6.m	**Acquired Time**	7/7/2021 11:16:21 AM
**IRM Calibration Status**	Not Applicable	**DA Method**	ChromPeakSurvey-Default.m
**Comment**			

Sample Group
Stream Name LC 1

Info.
Acquisition SW Version 6400 Series Triple Quadrupole 10.0 (127)

User Chromatograms

Fragmentor Voltage 135 **Collision Energy** 0 **Ionization Mode** ESI

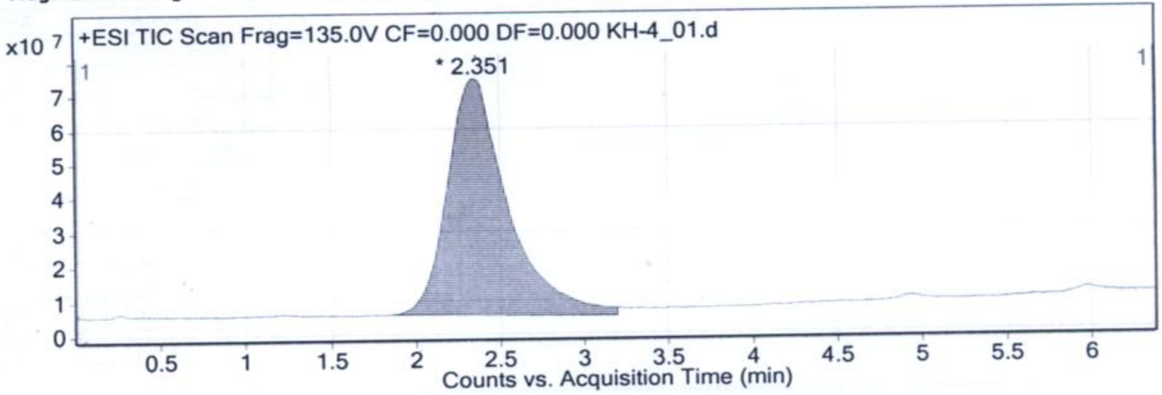

Integration Peak List

Peak	Start	RT	End	Height	Area	Area %
1	1,851	2,351	3,194	69205467	1716905206	100

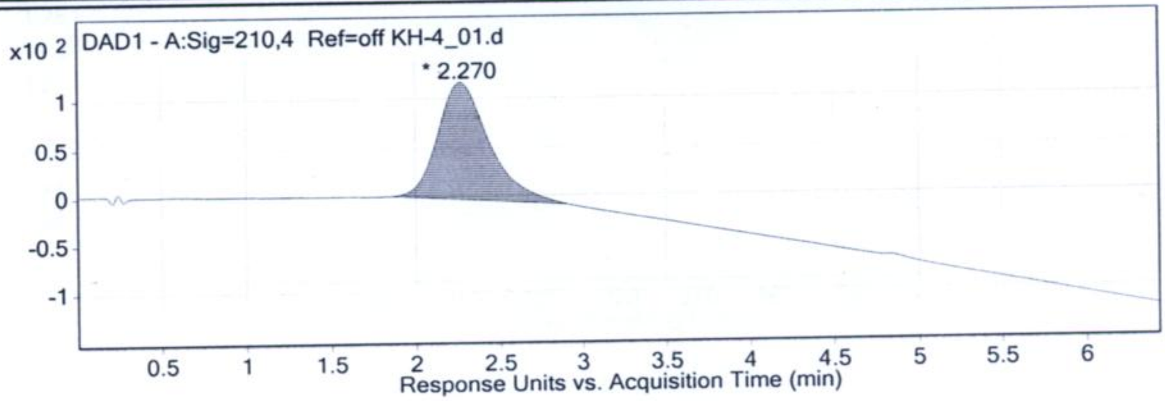

Integration Peak List

Peak	Start	RT	End	Height	Area	Area %
1	1,877	2,27	2,904	121,22	2578,78	100

User Spectra

**Spectrum Source**	**Fragmentor Voltage**	**Collision Energy**	**Ionization Mode**
Peak (1) in "+ TIC Scan"	135	0	ESI

Qualitative Analysis Report

3a

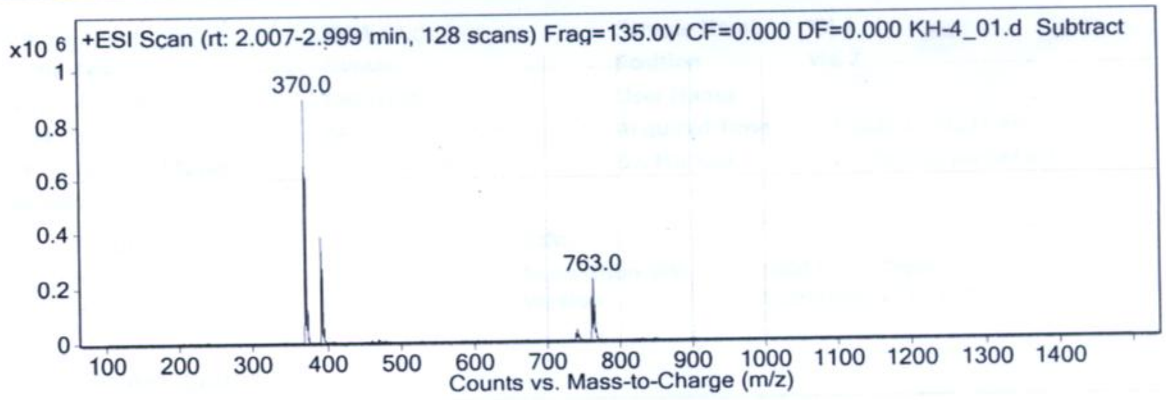

Peak List

m/z	z	Abund
370	1	896106.25
371	1	148190.73
372	1	609470.75
373	1	103675.24
374	1	118506.73
392	1	386740.41
394	1	270099.34
761		154753.8
763	1	224958.2
765	1	127922.04

Spectrum Source

Peak (1) in "DAD1 - A:Sig=210,4 Ref=off"

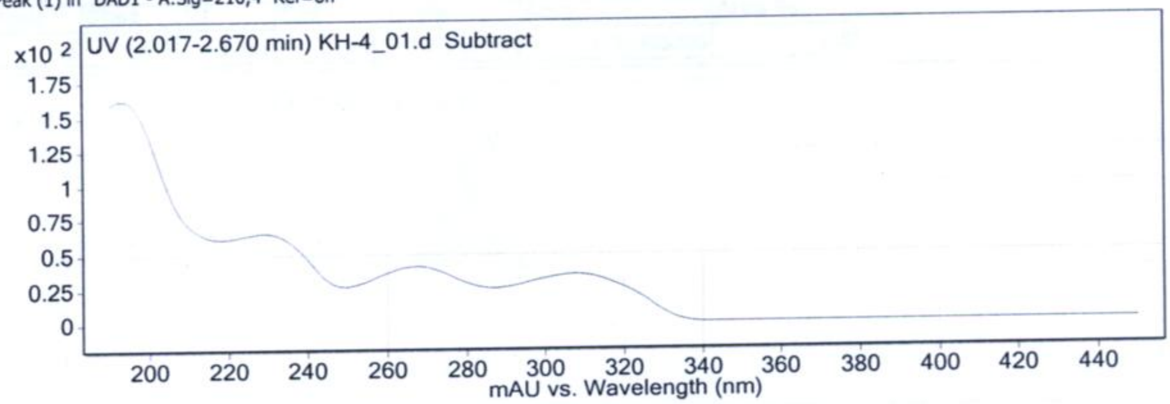

--- End Of Report ---

Qualitative Analysis Report

3b

**Data Filename**	KH-6_01.d	**Sample Name**	KH-6
**Sample Type**	Sample	**Position**	Vial 2
**Instrument Name**	Instrument 1	**User Name**	
**Acq Method**	All_2021_kol 1-6.m	**Acquired Time**	7/7/2021 11:39:16 AM
**IRM Calibration Status**	Not Applicable	**DA Method**	ChromPeakSurvey-Default.m
**Comment**			

**Sample Group**		**Info.**	
**Stream Name**	LC 1	**Acquisition SW Version**	6400 Series Triple Quadrupole 10.0 (127)

User Chromatograms

Fragmentor Voltage 135 **Collision Energy** 0 **Ionization Mode** ESI

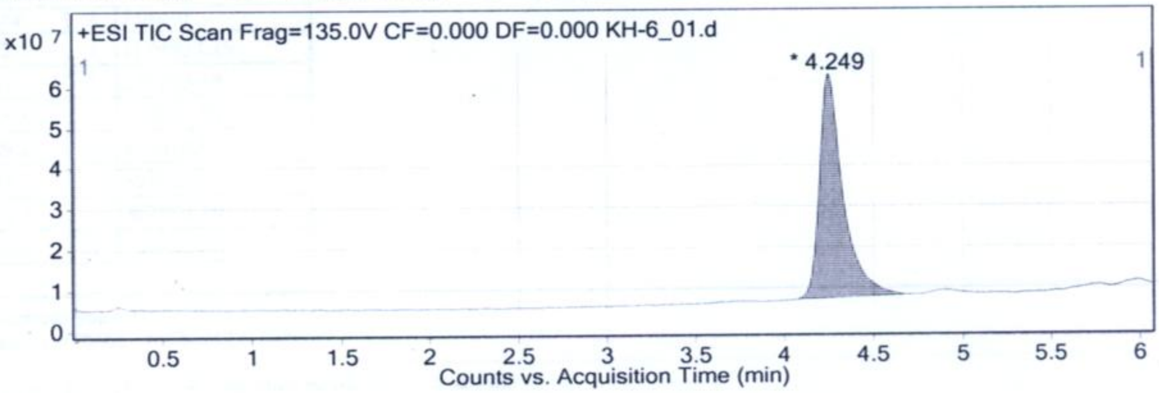

Integration Peak List

Peak	Start	RT	End	Height	Area	Area %
1	4,077	4,249	4,694	55170028	503867286	100

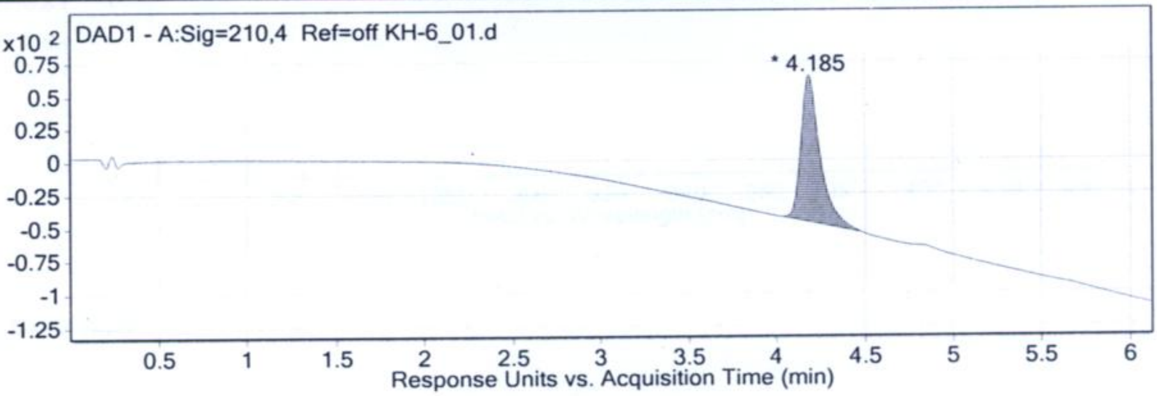

Integration Peak List

Peak	Start	RT	End	Height	Area	Area %
1	4,045	4,185	4,471	111,12	819,41	100

User Spectra

Spectrum Source	Fragmentor Voltage	Collision Energy	Ionization Mode
Peak (1) in "+ TIC Scan"	135	0	ESI

Qualitative Analysis Report

3b

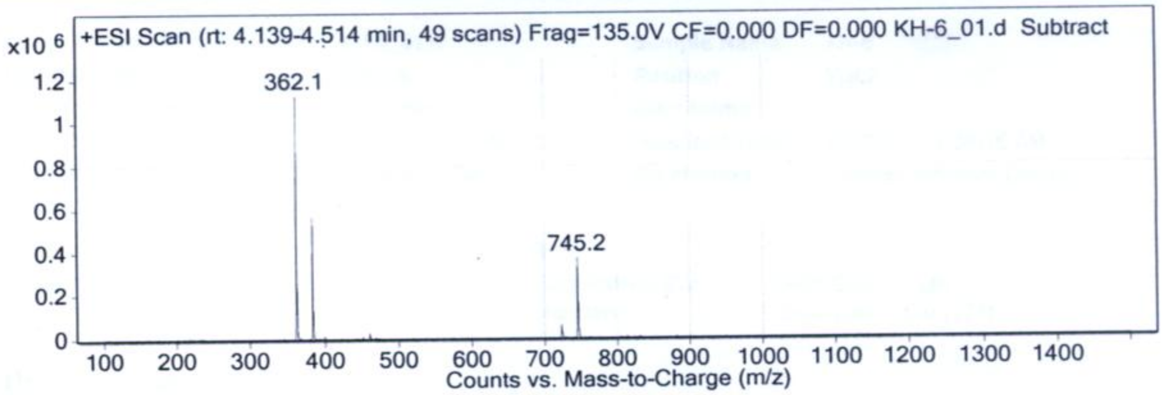

Peak List

m/z	z	Abund
362.1	1	1124933.25
363.1	1	237955.14
384.1	1	564864.88
385.1	1	130870.91
723.3		59793.97
745.2	1	367083.19
746.2	1	166852.56

Spectrum Source

Peak (1) in "DAD1 - A:Sig=210,4 Ref=off"

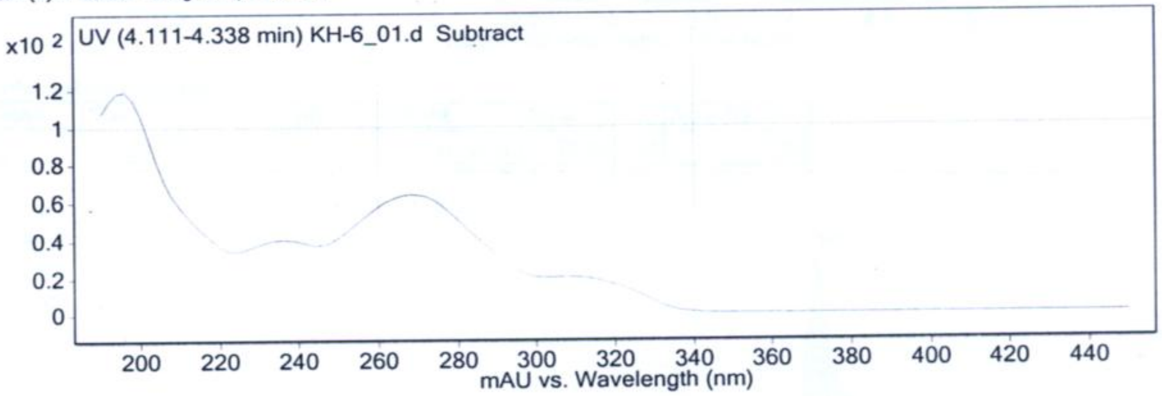

--- End Of Report ---

Qualitative Analysis Report

3c

**Data Filename**	KH-5_01.d	**Sample Name**	KH-5
**Sample Type**	Sample	**Position**	Vial 2
**Instrument Name**	Instrument 1	**User Name**	
**Acq Method**	All_2021_kol 1-6.m	**Acquired Time**	7/7/2021 11:27:00 AM
**IRM Calibration Status**	Not Applicable	**DA Method**	ChromPeakSurvey-Default.m
**Comment**			

**Sample Group**		**Info.**	
**Stream Name**	LC 1	**Acquisition SW Version**	6400 Series Triple Quadrupole 10.0 (127)

User Chromatograms

Fragmentor Voltage 135 **Collision Energy** 0 **Ionization Mode** ESI

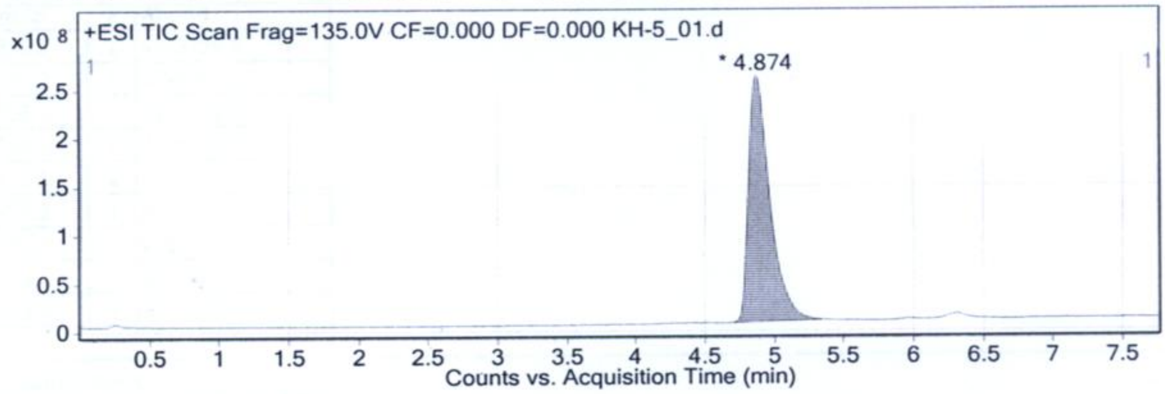

Integration Peak List

Peak	Start	RT	End	Height	Area	Area %
1	4,702	4,874	5,35	254886638	2721539730	100

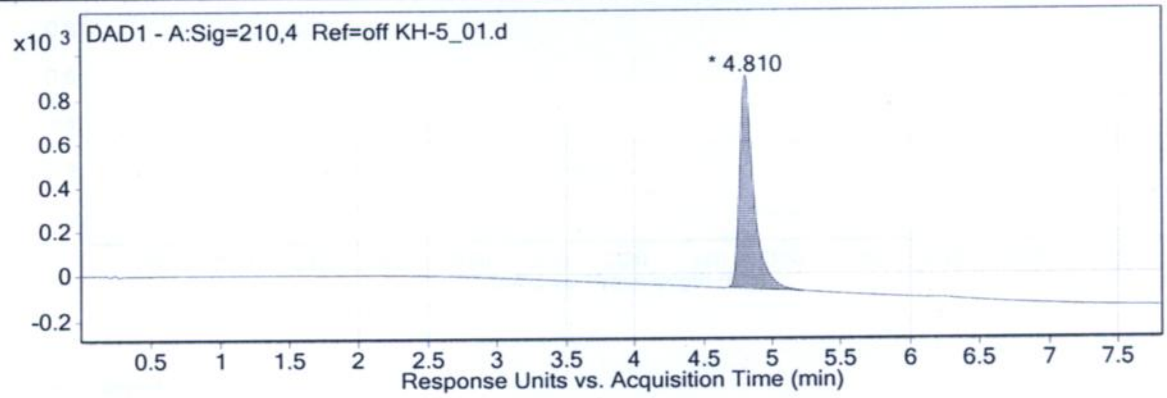

Integration Peak List

Peak	Start	RT	End	Height	Area	Area %
1	4,683	4,81	5,23	961,45	6805,64	100

User Spectra

Spectrum Source	Fragmentor Voltage	Collision Energy	Ionization Mode
Peak (1) in "+ TIC Scan"	135	0	ESI

Qualitative Analysis Report

3c

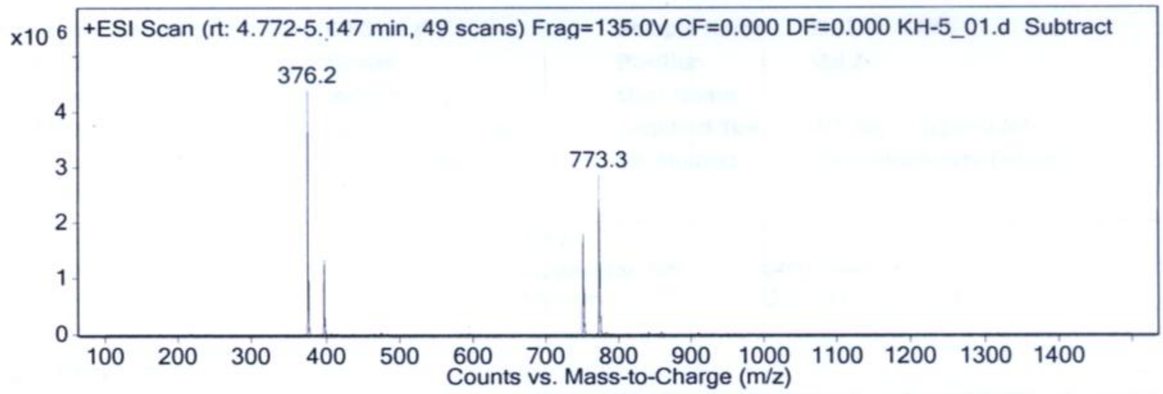

Peak List

m/z	z	Abund
376.2	1	4403386.5
377.2	1	969849.31
398.1	1	1339133.63
399.1	1	303984.28
751.3	1	1798671.63
752.3	1	817681.63
773.3	1	2867851.75
774.3	1	1301444.75
775.3	1	324454.59

Spectrum Source

Peak (1) in "DAD1 - A:Sig=210,4 Ref=off"

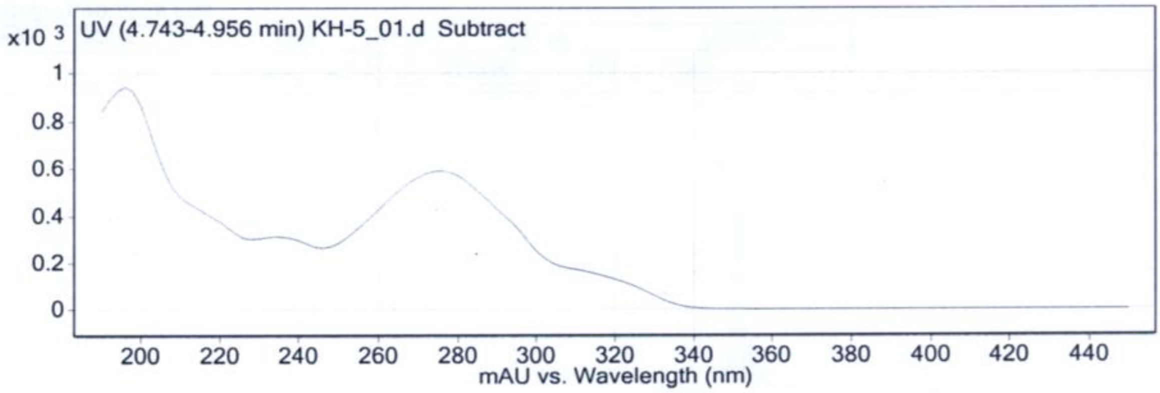

--- End Of Report ---

Qualitative Analysis Report

3f

**Data Filename**	KH-9_01.d	**Sample Name**	KH-9
**Sample Type**	Sample	**Position**	Vial 2
**Instrument Name**	Instrument 1	**User Name**	
**Acq Method**	All_2021_kol 1-6.m	**Acquired Time**	7/7/2021 12:20:00 PM
**IRM Calibration Status**	Not Applicable	**DA Method**	ChromPeakSurvey-Default.m
**Comment**			

**Sample Group**		**Info.**	
**Stream Name**	LC 1	**Acquisition SW Version**	6400 Series Triple Quadrupole 10.0 (127)

User Chromatograms

Fragmentor Voltage 135 **Collision Energy** 0 **Ionization Mode** ESI

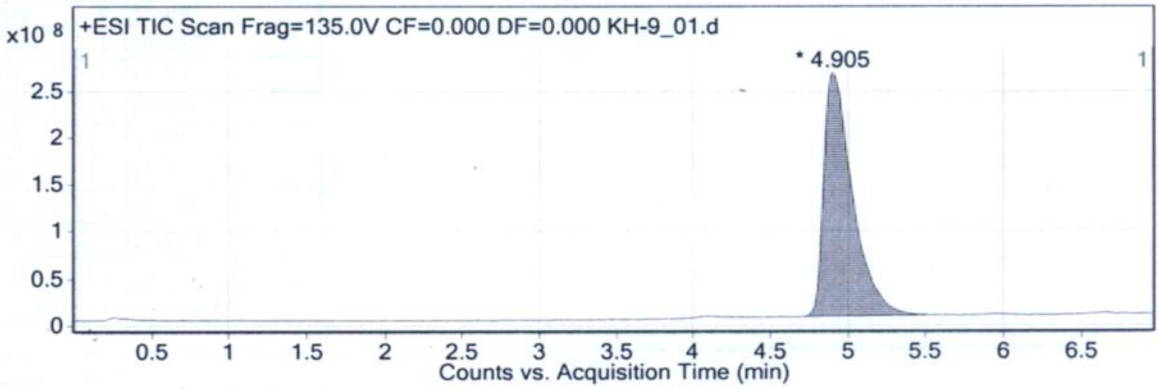

Integration Peak List

Peak	Start	RT	End	Height	Area	Area %
1	4,717	4,905	5,537	259456426	3335376360	100

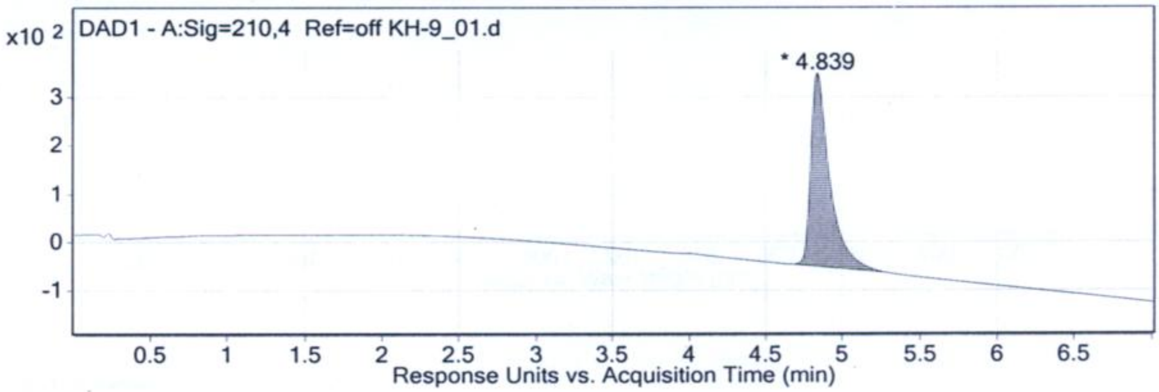

Integration Peak List

Peak	Start	RT	End	Height	Area	Area %
1	4,679	4,839	5,272	399,32	3392,55	100

User Spectra

Spectrum Source	Fragmentor Voltage	Collision Energy	Ionization Mode
Peak (1) in "+ TIC Scan"	135	0	ESI

Qualitative Analysis Report

3f

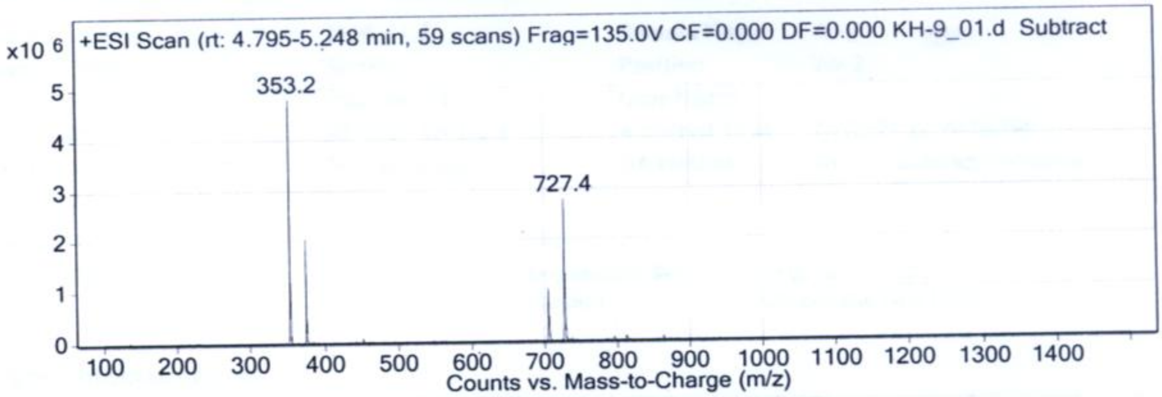

Peak List

m/z	z	Abund
353.2	1	4806573
354.2	1	1197329.5
375.2	1	2040541
376.2	1	449387.5
705.4	1	1023337.25
706.4	1	467385.91
727.4	1	2790041.25
728.4	1	1322313.88
729.4	1	298360.22

Spectrum Source

Peak (1) in "DAD1 - A:Sig=210,4 Ref=off"

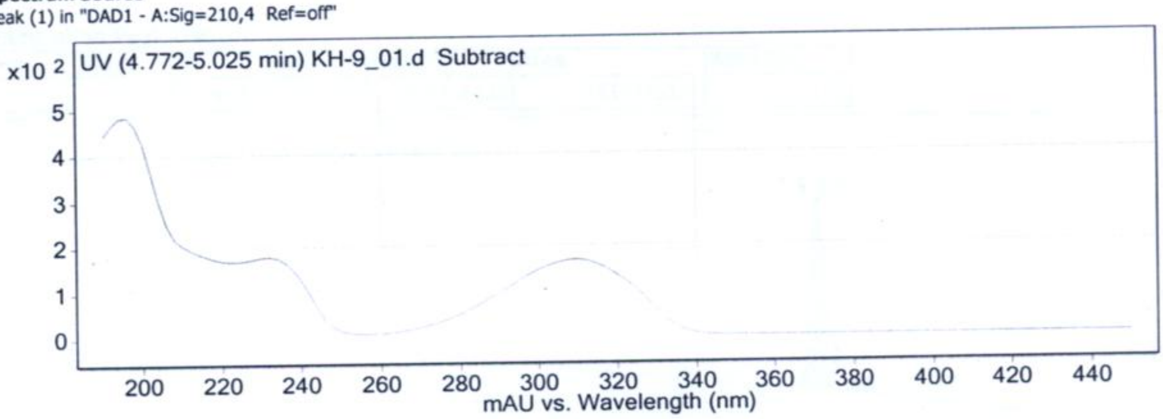

--- End Of Report ---
