# Supplementary material for: Synthesis and Biological Activity of N-acyl Anabasine and Cytisine Derivatives with Adamantane, Pyridine and 1,2-Azole Fragments
Source: Molecules. 2022 Oct 31;27(21):7387. doi: 10.3390/molecules27217387 (PMC9656753; doi:10.3390/molecules27217387)
Supplement: Supplementary file 1 [file molecules-27-07387-s001.zip › NMR.pdf]

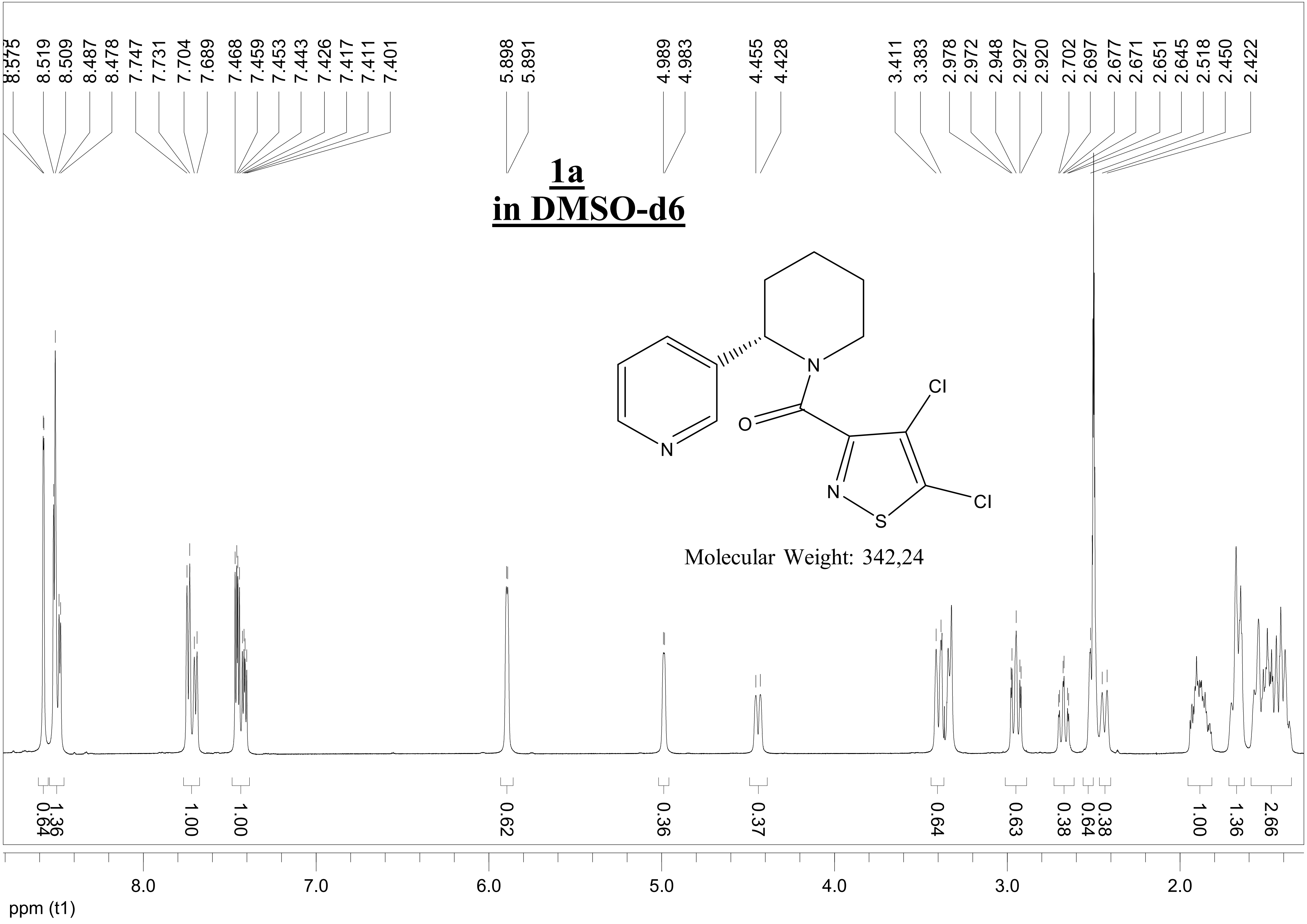
1a
in DMSO-d6
8.575
8.519
8.509
8.487
8.478
7.747
7.731
7.704
7.689
7.468
7.459
7.453
7.443
7.426
7.417
7.411
7.401
5.898
5.891
4.989
4.983
4.455
4.428
3.411
3.383
2.978
2.972
2.948
2.927
2.920
2.702
2.697
2.677
2.671
2.651
2.645
2.518
2.450
2.422
N
N
O
Cl
Cl
N
S
Molecular Weight: 342,24
0.64
1.36
1.00
1.00
0.62
0.36
0.37
0.64
0.63
0.38
0.64
0.38
1.00
1.36
2.66
8.0
7.0
6.0
5.0
4.0
3.0
2.0
ppm (t1)

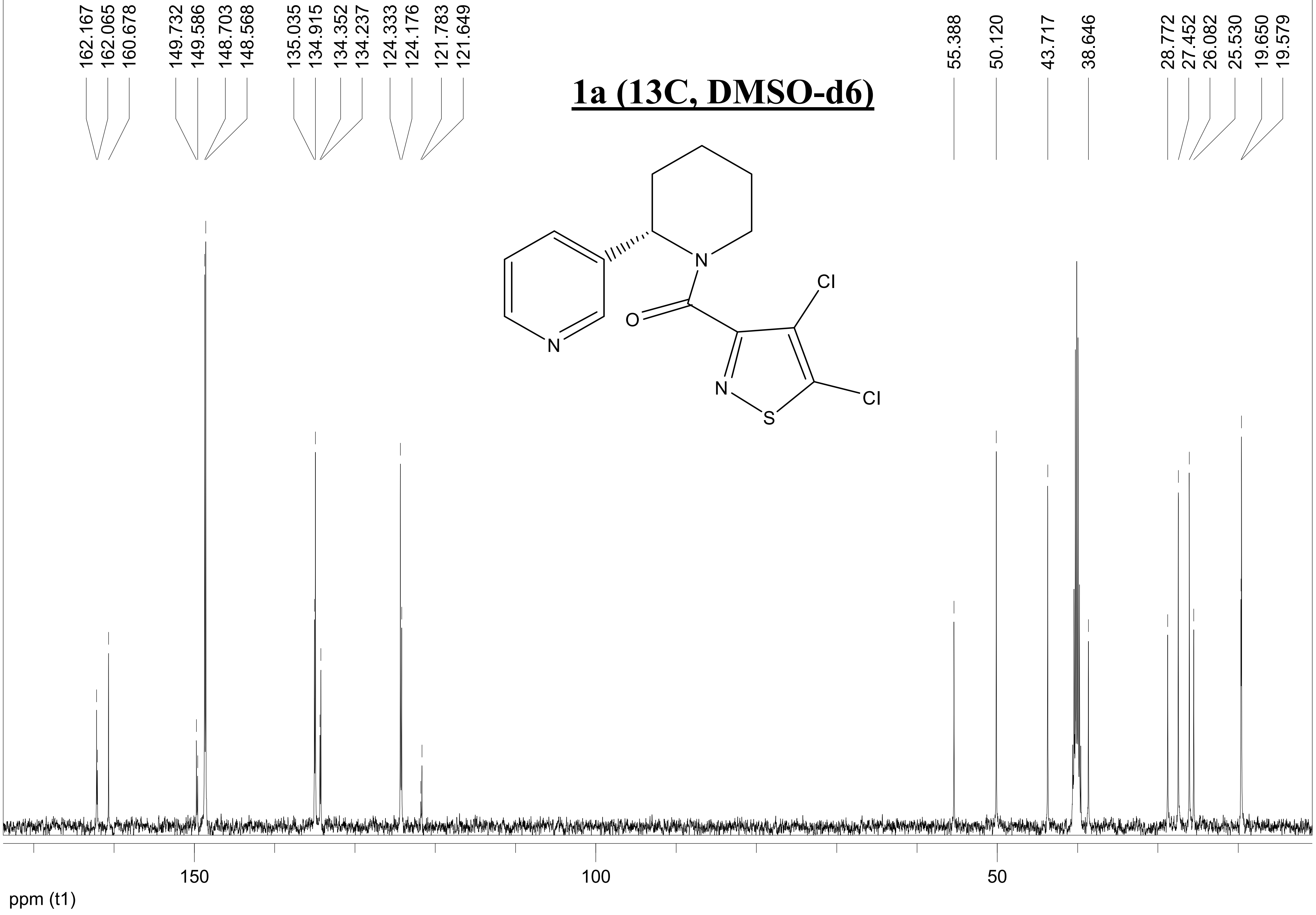
162.167
162.065
160.678
149.732
149.586
148.703
148.568
135.035
134.915
134.352
134.237
124.333
124.176
121.783
121.649
55.388
50.120
43.717
38.646
28.772
27.452
26.082
25.530
19.650
19.579
1a (13C, DMSO-d6)
N
N
O
Cl
N
S
Cl
150
100
50
ppm (t1)

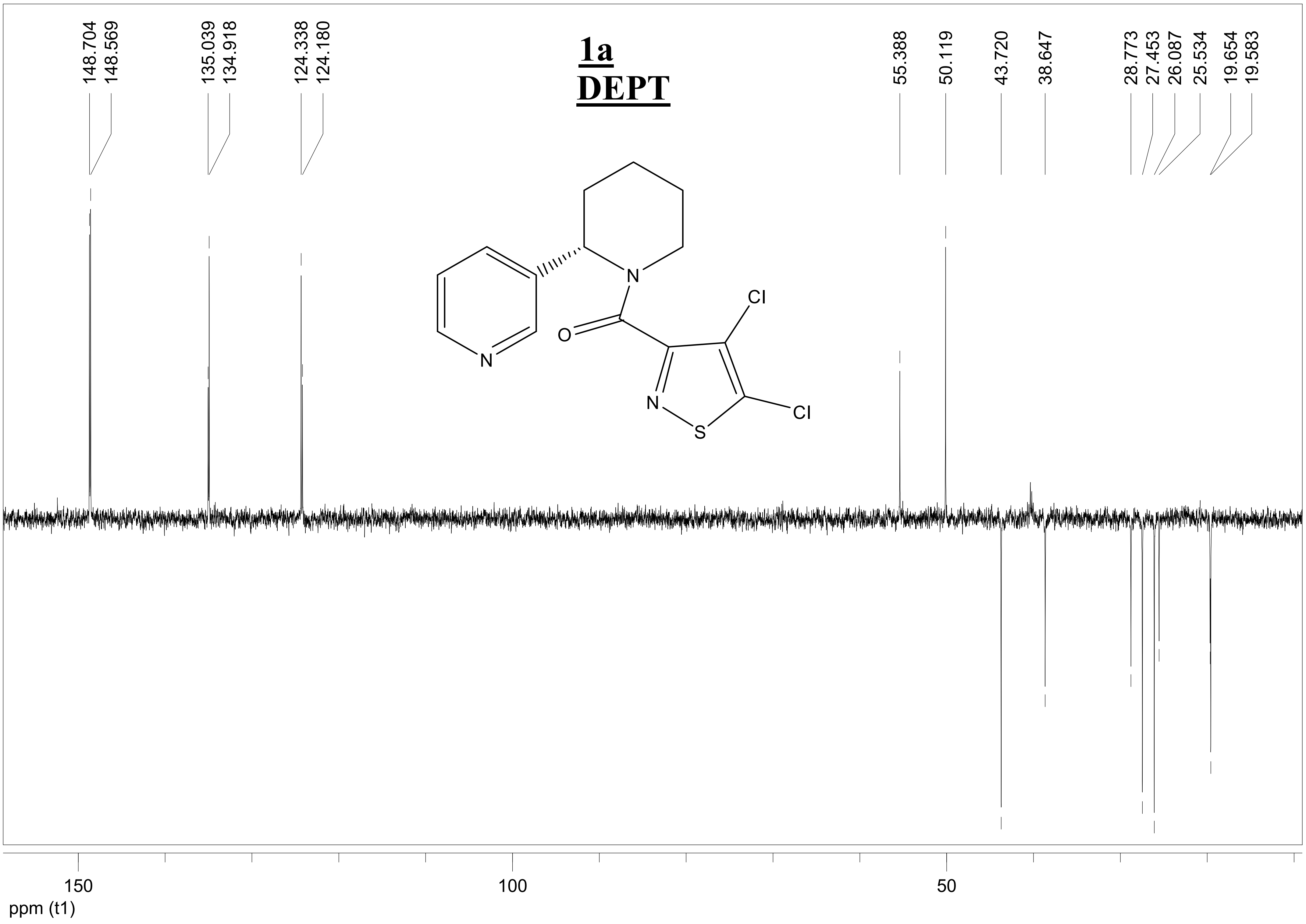
1a
DEPT
148.704
148.569
135.039
134.918
124.338
124.180
55.388
50.119
43.720
38.647
28.773
27.453
26.087
25.534
19.654
19.583
N
N
O
Cl
N
S
Cl
150
100
50
ppm (t1)

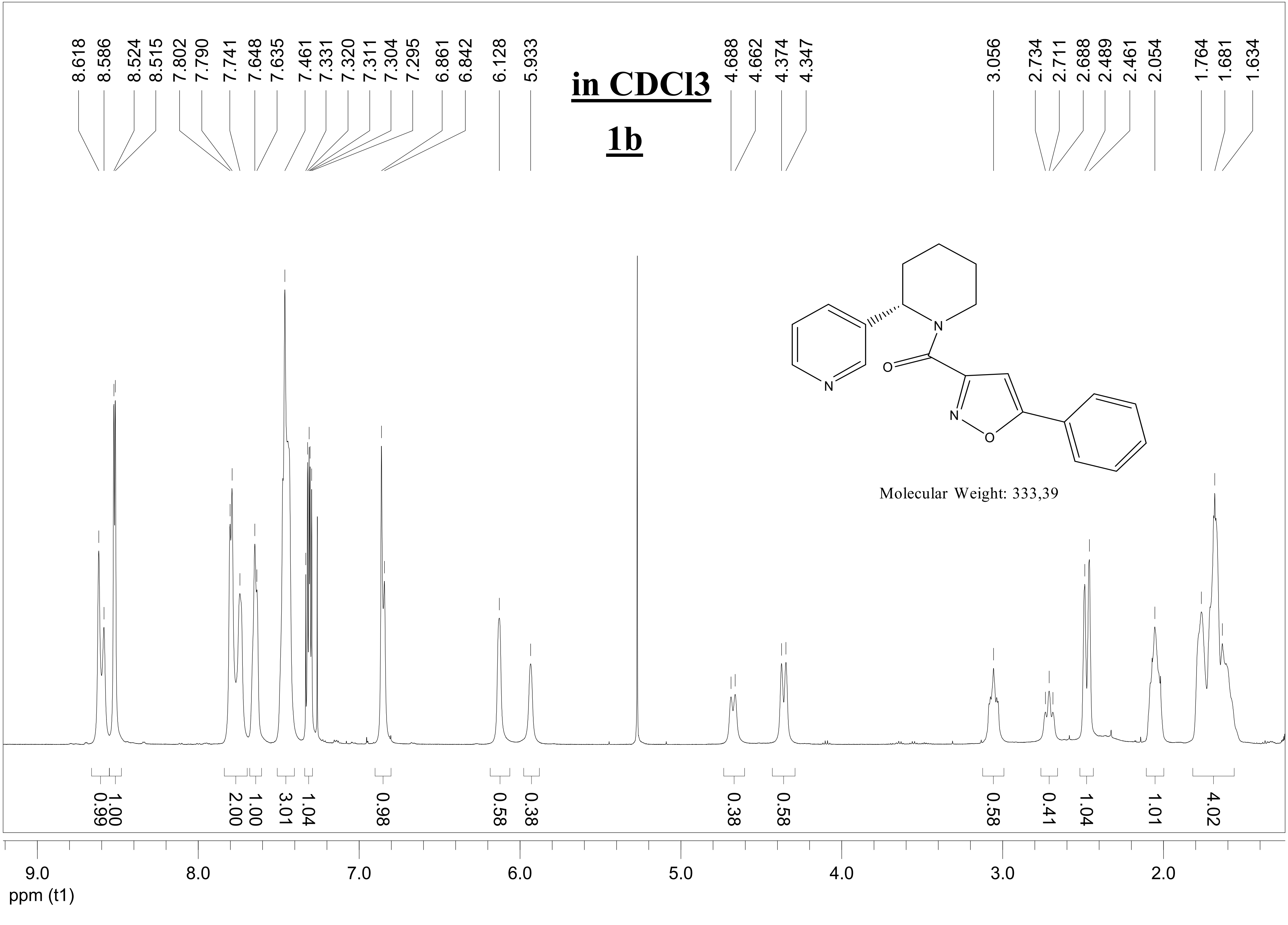

in CDCl3
1b
8.618 8.586 8.524 8.515 7.802 7.790 7.741 7.648 7.635 7.461 7.331 7.320 7.311 7.304 7.295 6.861 6.842 6.128 5.933 4.688 4.662 4.374 4.347 3.056 2.734 2.711 2.688 2.489 2.461 2.054 1.764 1.681 1.634
N
O
N
O
Molecular Weight: 333,39
0.99 1.00 2.00 1.00 3.01 1.04 0.98 0.58 0.38 0.38 0.58 0.58 0.41 1.04 1.01 4.02
9.0 8.0 7.0 6.0 5.0 4.0 3.0 2.0
ppm (t1)

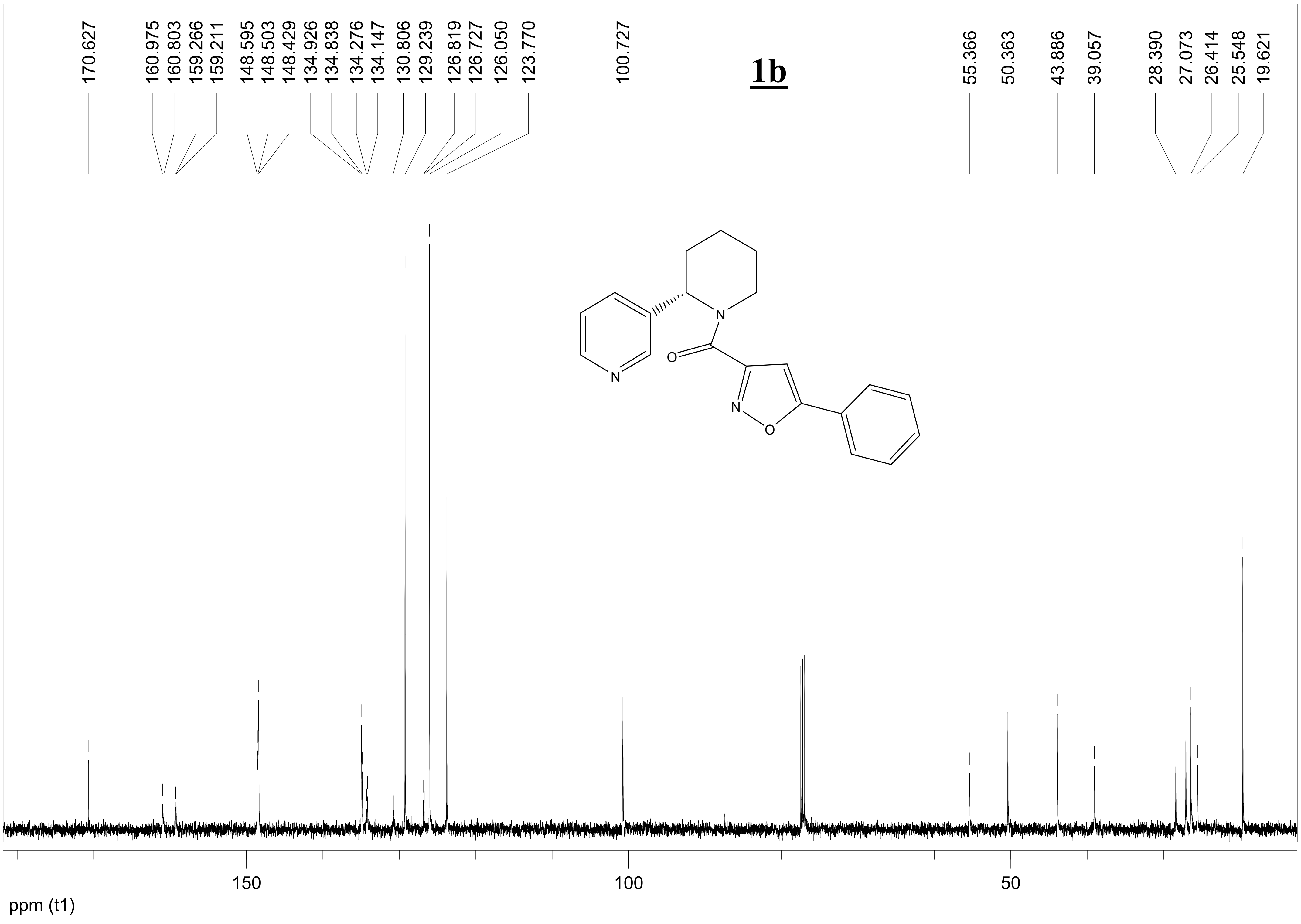
170.627
160.975
160.803
159.266
159.211
148.595
148.503
148.429
134.926
134.838
134.276
134.147
130.806
129.239
126.819
126.727
126.050
123.770
100.727
1b
55.366
50.363
43.886
39.057
28.390
27.073
26.414
25.548
19.621
N
O
N
O
N
150
100
50
ppm (t1)

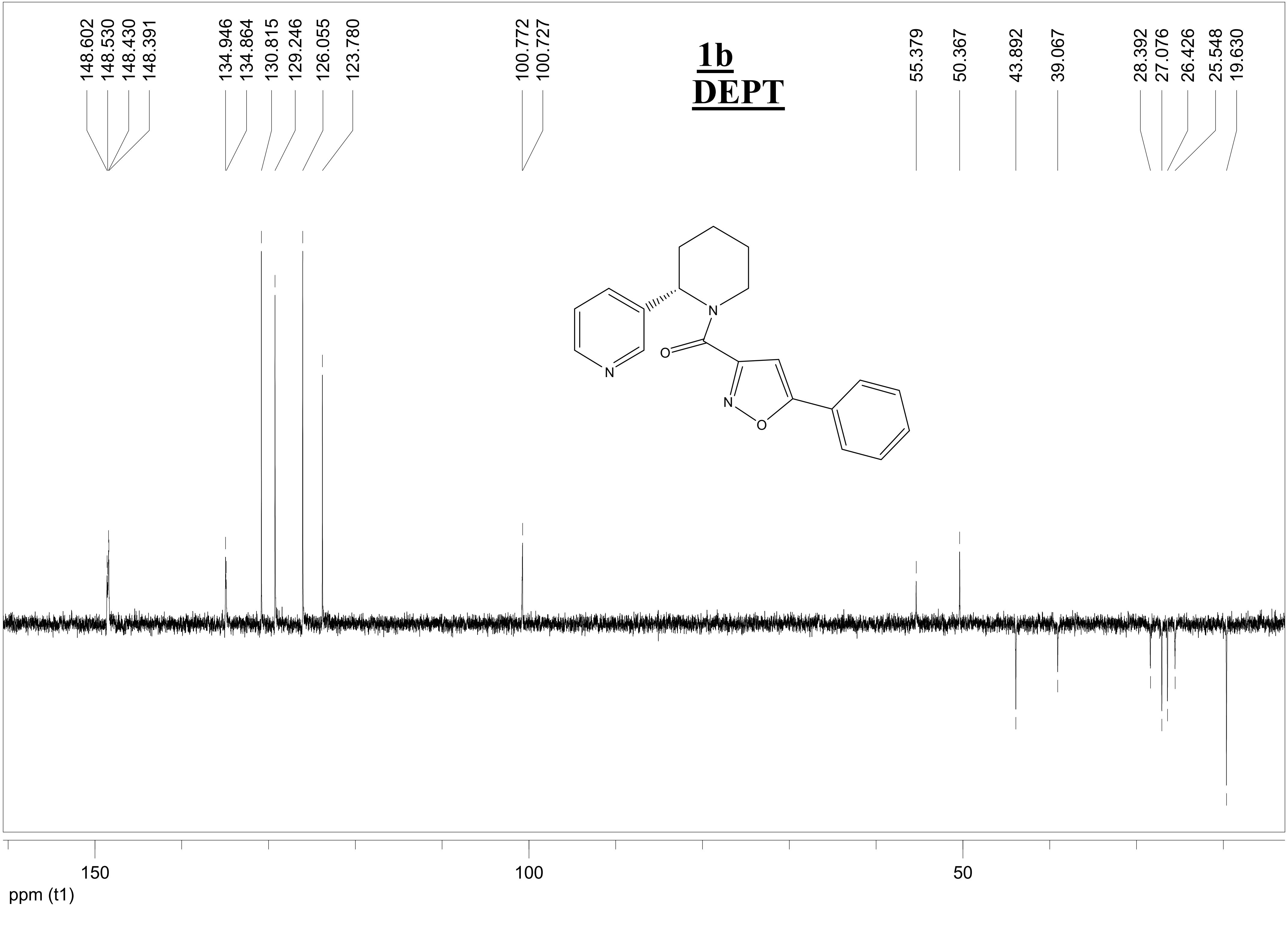
148.602
148.530
148.430
148.391
134.946
134.864
130.815
129.246
126.055
123.780
100.772
100.727
55.379
50.367
43.892
39.067
28.392
27.076
26.426
25.548
19.630
1b
DEPT
N
O
N
O
N
150
100
50
ppm (t1)

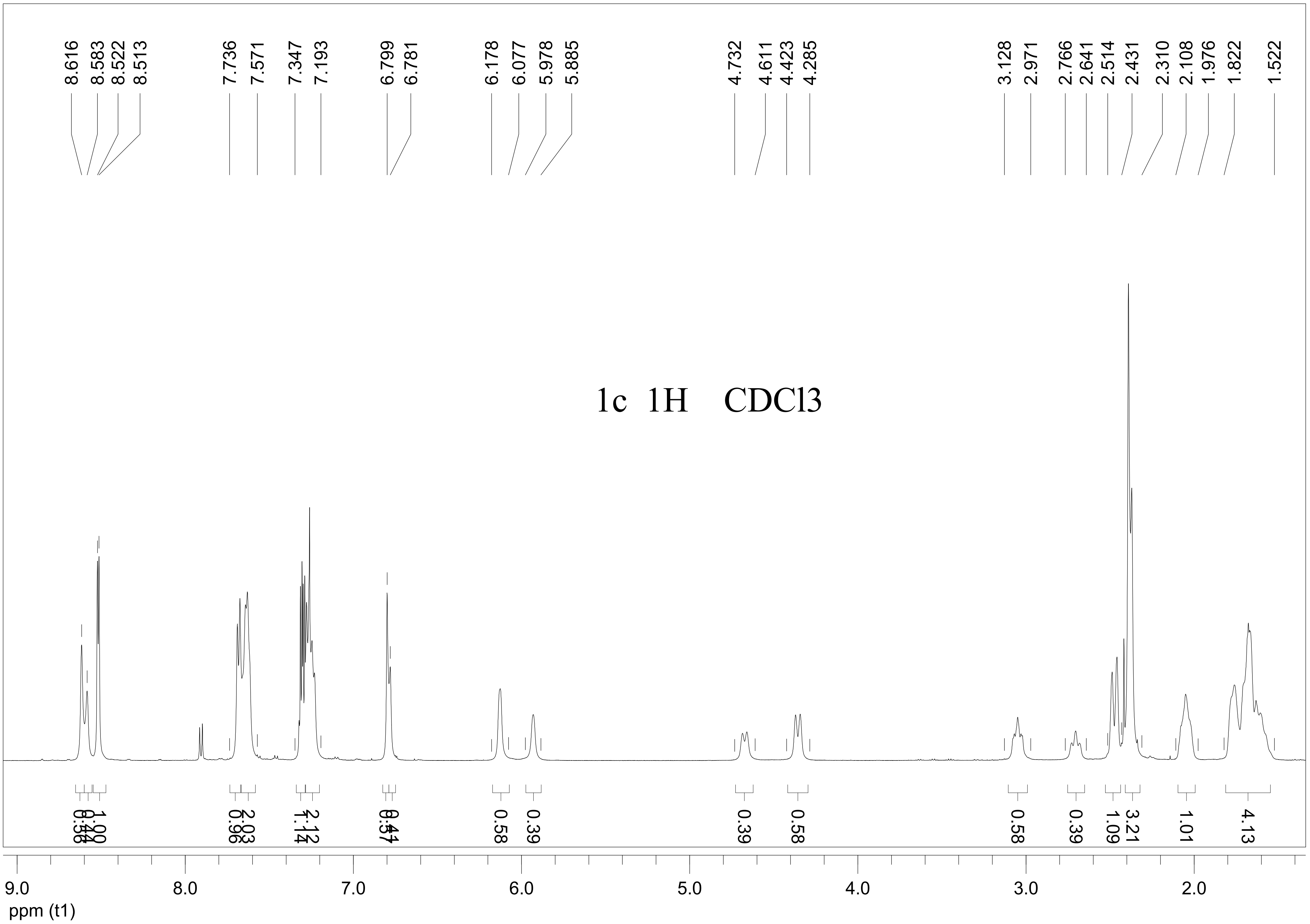

8.616 8.583 8.522 8.513
7.736 7.571 7.347 7.193
6.799 6.781
6.178 6.077 5.978 5.885
4.732 4.611 4.423 4.285
3.128 2.971 2.766 2.641 2.514 2.431 2.310 2.108 1.976 1.822 1.522
1c 1H CDCl3
1.00 2.03 2.12 0.58 0.39 0.39 0.58 0.58 0.39 1.09 3.21 1.01 4.13
9.0 8.0 7.0 6.0 5.0 4.0 3.0 2.0
ppm (t1)

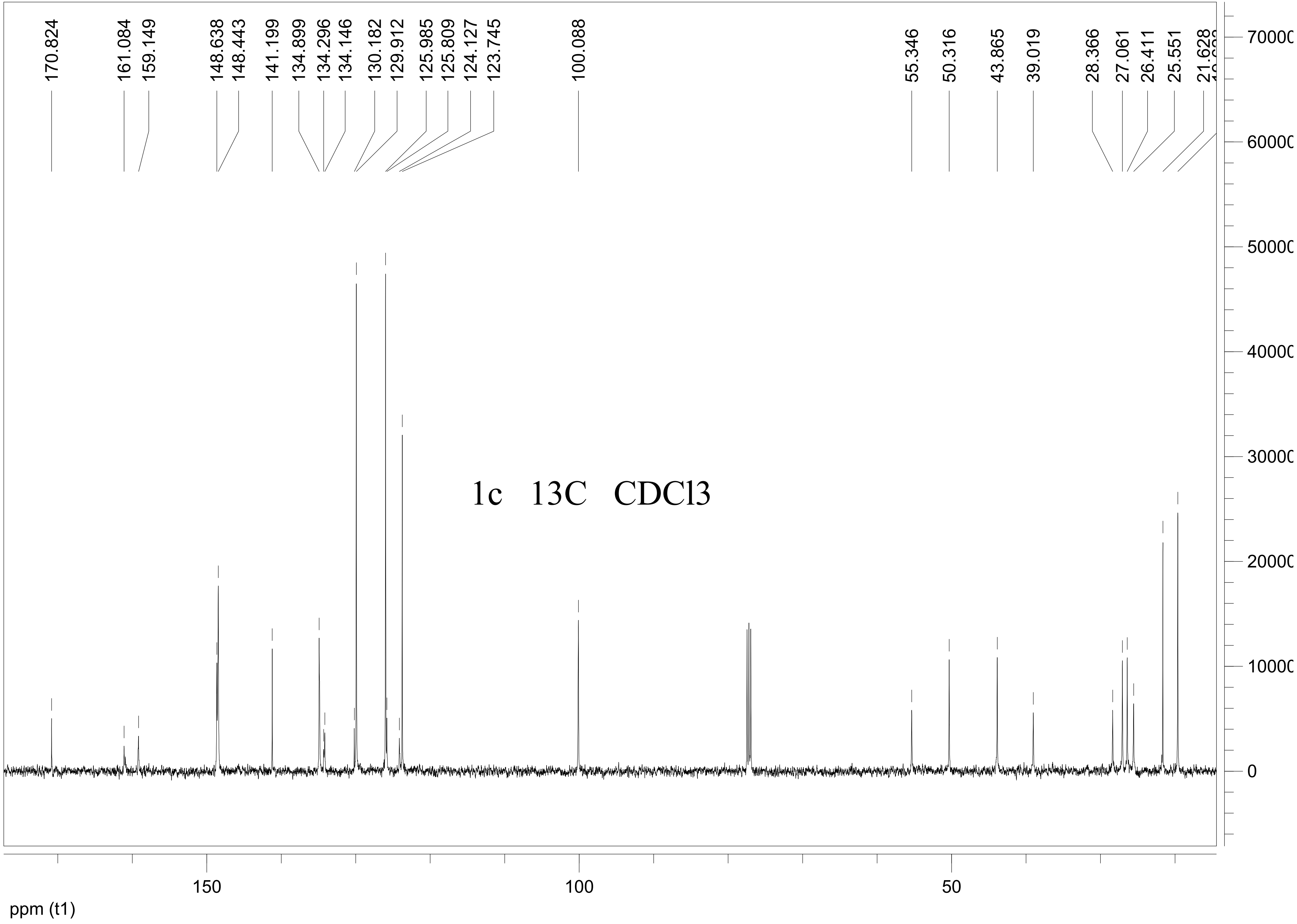
170.824
161.084
159.149
148.638
148.443
141.199
134.899
134.296
134.146
130.182
129.912
125.985
125.809
124.127
123.745
100.088
55.346
50.316
43.865
39.019
28.366
27.061
26.411
25.551
21.628
1c 13C CDCl3
150
100
50
ppm (t1)
0
10000
20000
30000
40000
50000
60000
70000

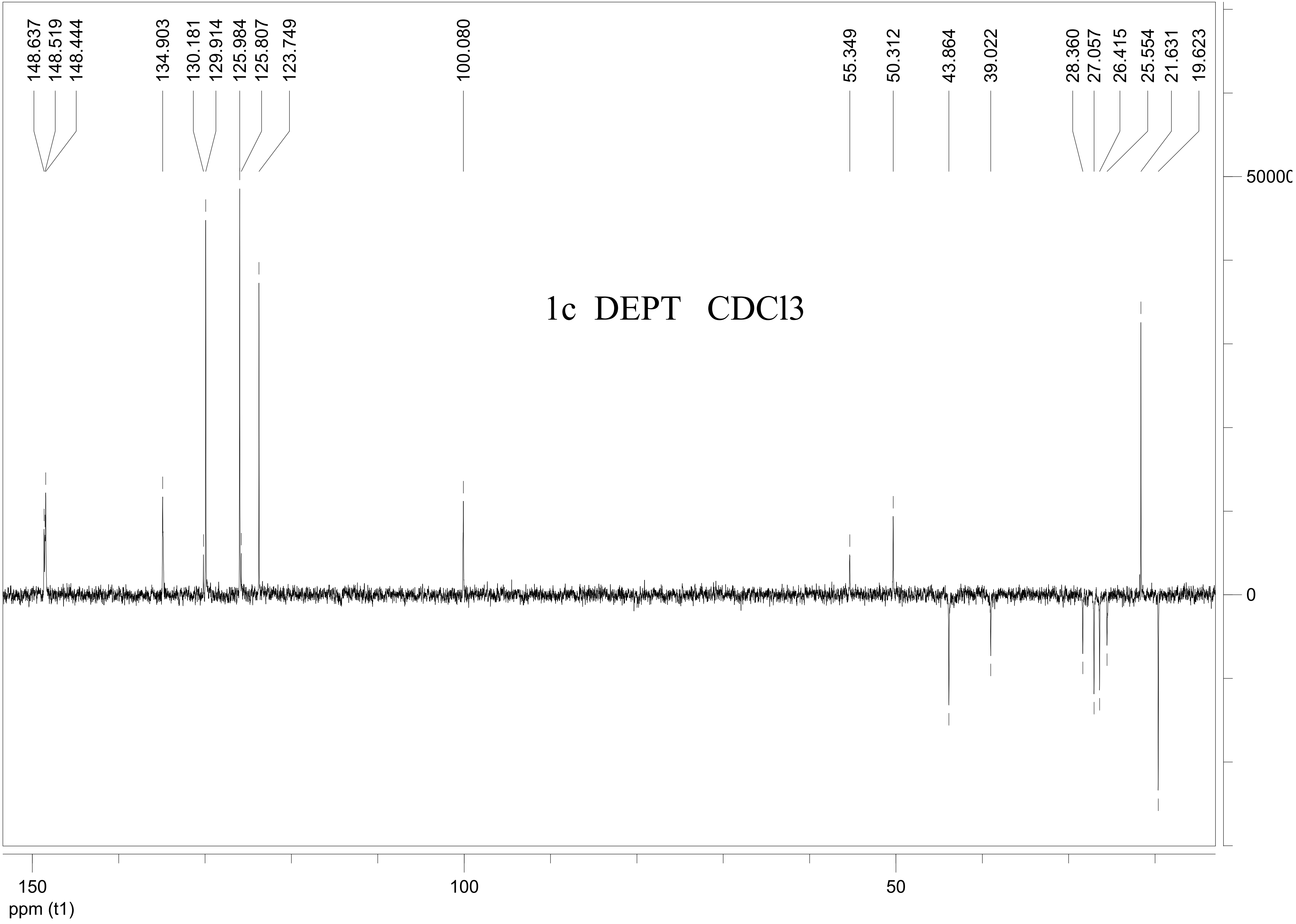
148.637
148.519
148.444
134.903
130.181
129.914
125.984
125.807
123.749
100.080
55.349
50.312
43.864
39.022
28.360
27.057
26.415
25.554
21.631
19.623
1c DEPT CDCl3
50000
0
150
100
50
ppm (t1)

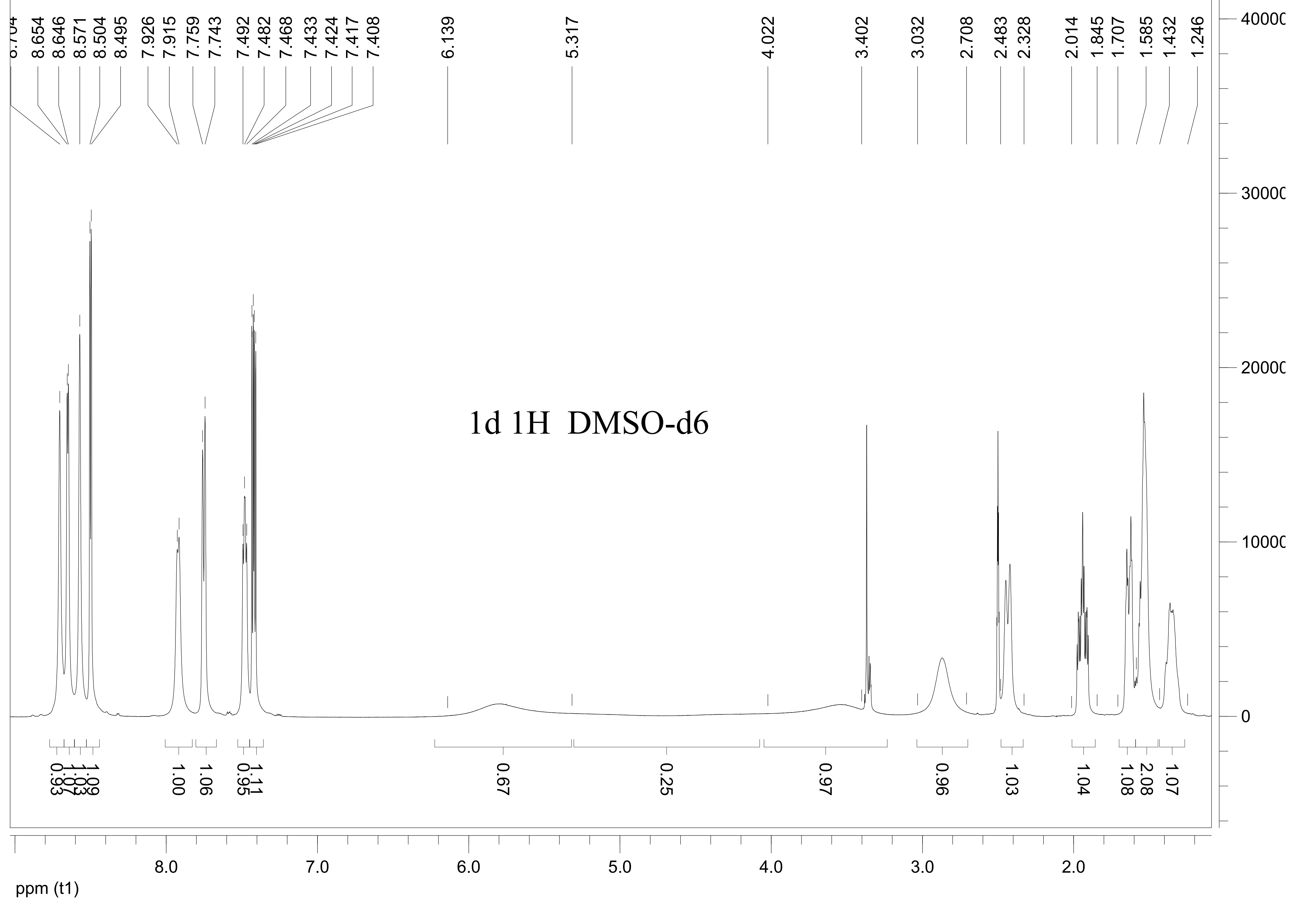

8.704
8.654
8.646
8.571
8.504
8.495
7.926
7.915
7.759
7.743
7.492
7.482
7.468
7.433
7.424
7.417
7.408
6.139
5.317
4.022
3.402
3.032
2.708
2.483
2.328
2.014
1.845
1.707
1.585
1.432
1.246
1d 1H DMSO-d6
0.93
1.07
1.03
1.09
1.00
1.06
0.95
1.11
0.67
0.25
0.97
0.96
1.03
1.04
1.08
2.08
1.07
40000
30000
20000
10000
0
8.0
7.0
6.0
5.0
4.0
3.0
2.0
ppm (t1)

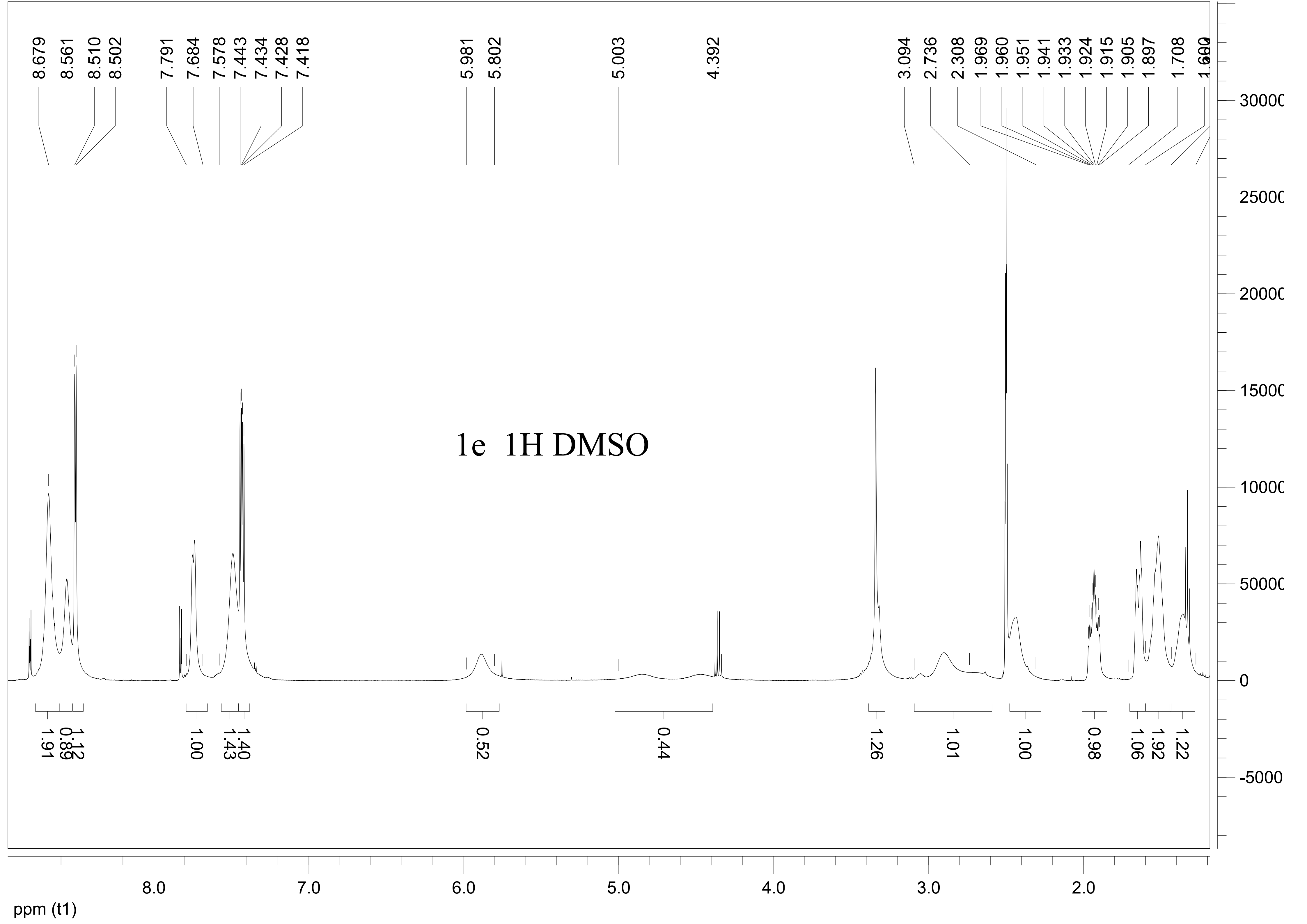

8.679
8.561
8.510
8.502
7.791
7.684
7.578
7.443
7.434
7.428
7.418
5.981
5.802
5.003
4.392
3.094
2.736
2.308
1.969
1.960
1.951
1.941
1.933
1.924
1.915
1.905
1.897
1.708
1e 1H DMSO
1.91
0.89
1.12
1.00
1.43
1.40
0.52
0.44
1.26
1.01
1.00
0.98
1.06
1.92
1.22
8.0
7.0
6.0
5.0
4.0
3.0
2.0
ppm (t1)

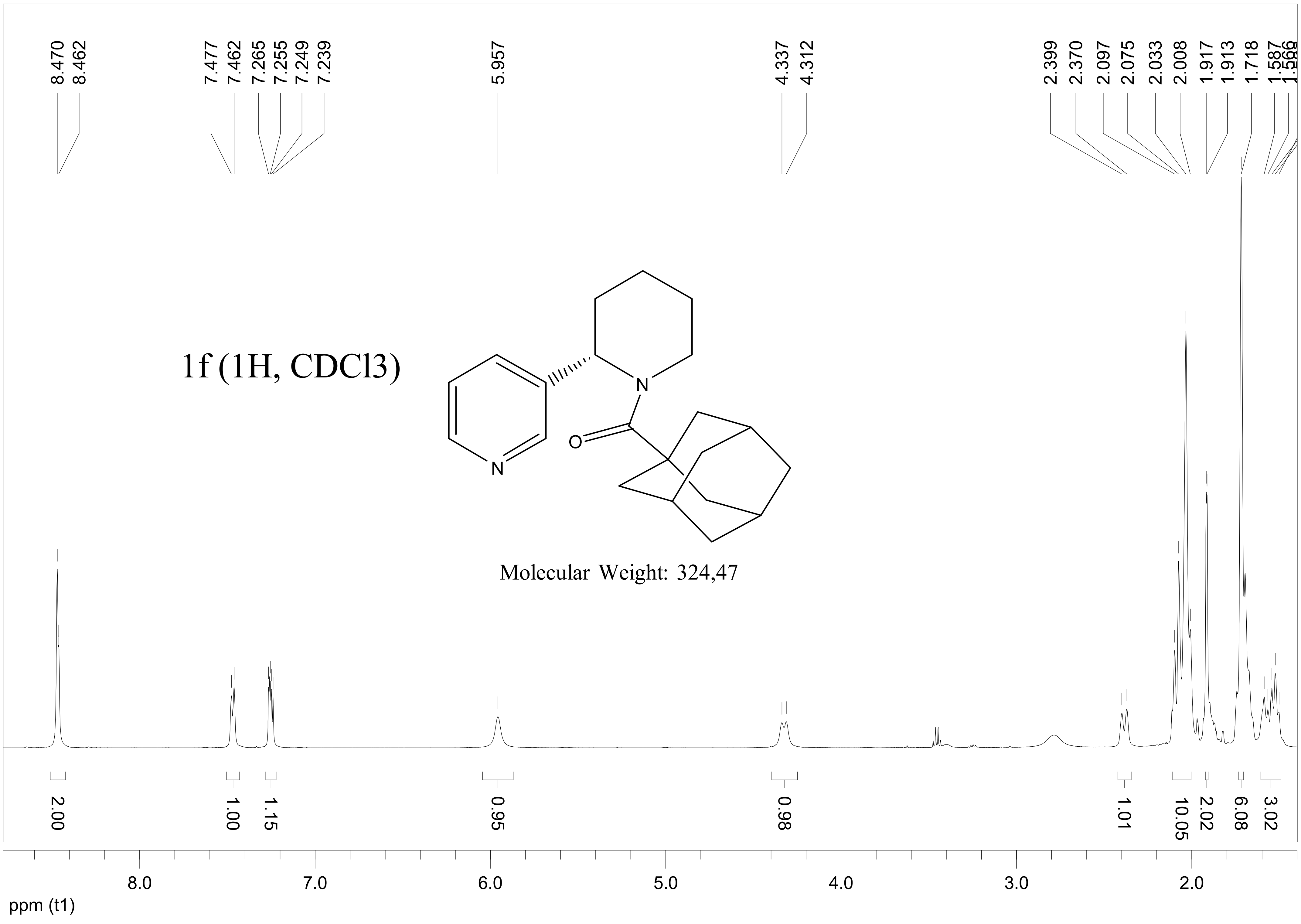

1f (1H, CDCl3)
Molecular Weight: 324,47
N
N
O
8.470
8.462
7.477
7.462
7.265
7.255
7.249
7.239
5.957
4.337
4.312
2.399
2.370
2.097
2.075
2.033
2.008
1.917
1.913
1.718
1.587
2.00
1.00
1.15
0.95
0.98
1.01
10.05
2.02
6.08
3.02
8.0
7.0
6.0
5.0
4.0
3.0
2.0
ppm (t1)

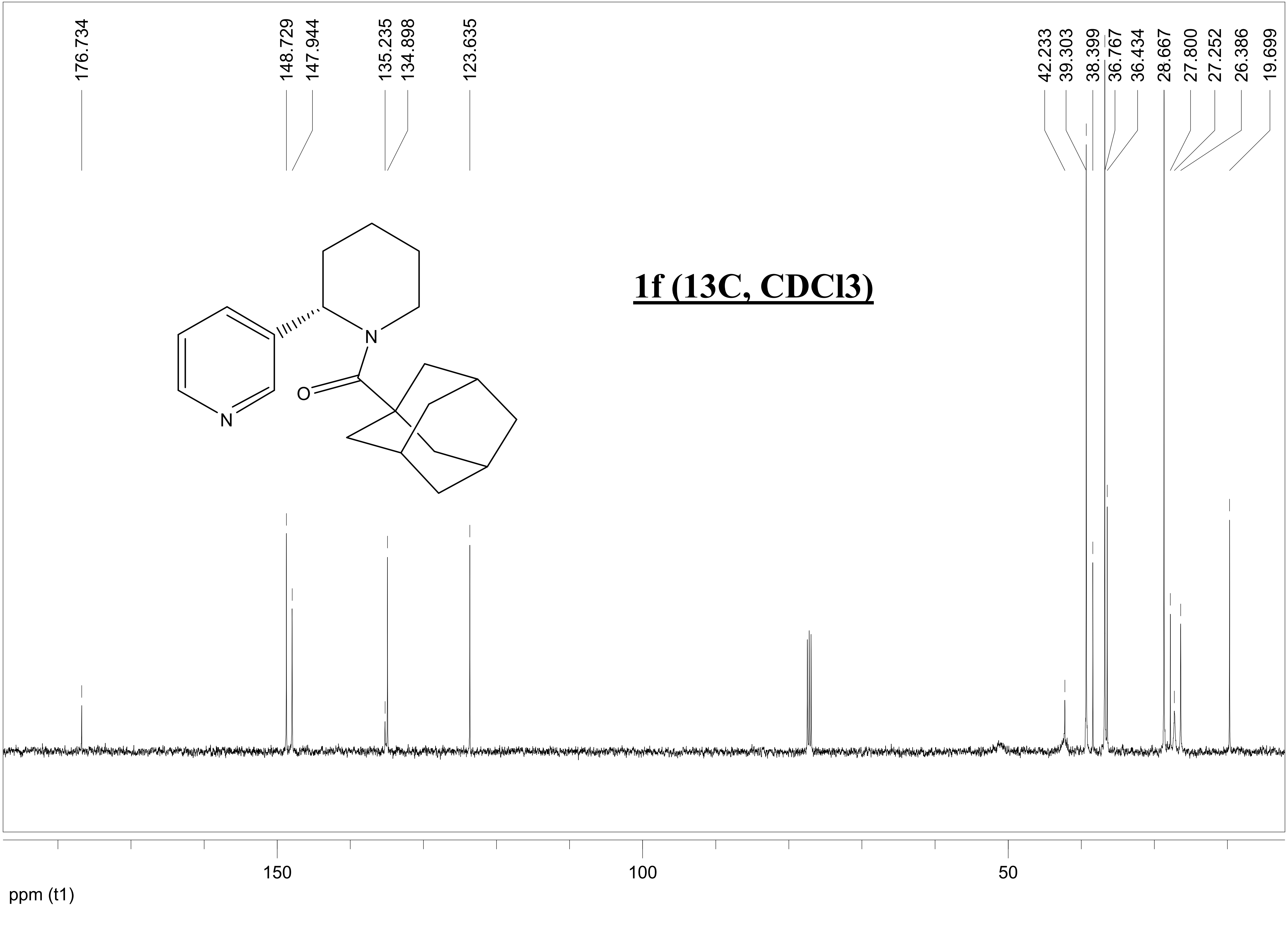
176.734
148.729
147.944
135.235
134.898
123.635
42.233
39.303
38.399
36.767
36.434
28.667
27.800
27.252
26.386
19.699
1f (13C, CDCl3)
N
N
O
150
100
50
ppm (t1)

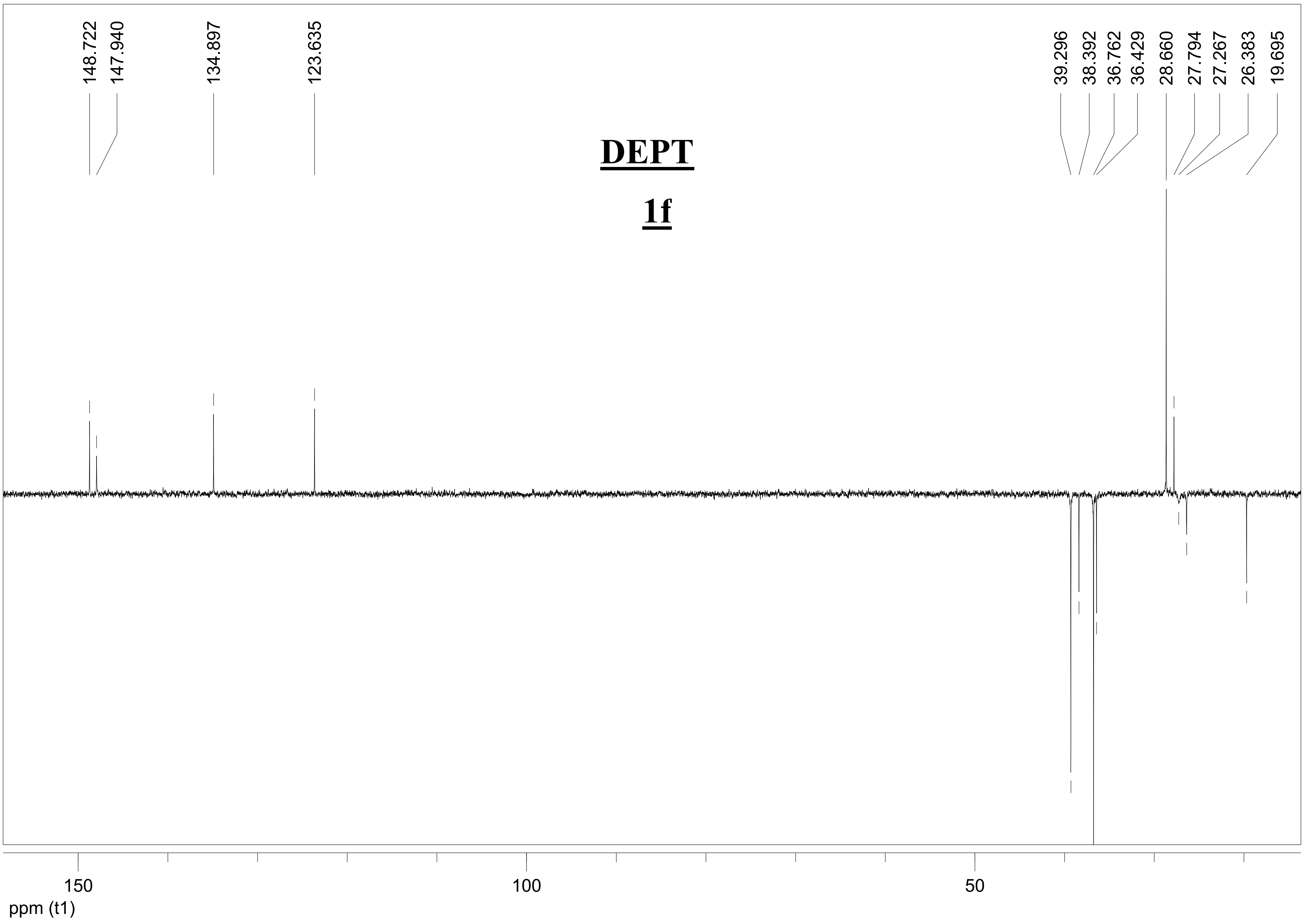
148.722
147.940
134.897
123.635
39.296
38.392
36.762
36.429
28.660
27.794
27.267
26.383
19.695
DEPT
1f
150
100
50
ppm (t1)

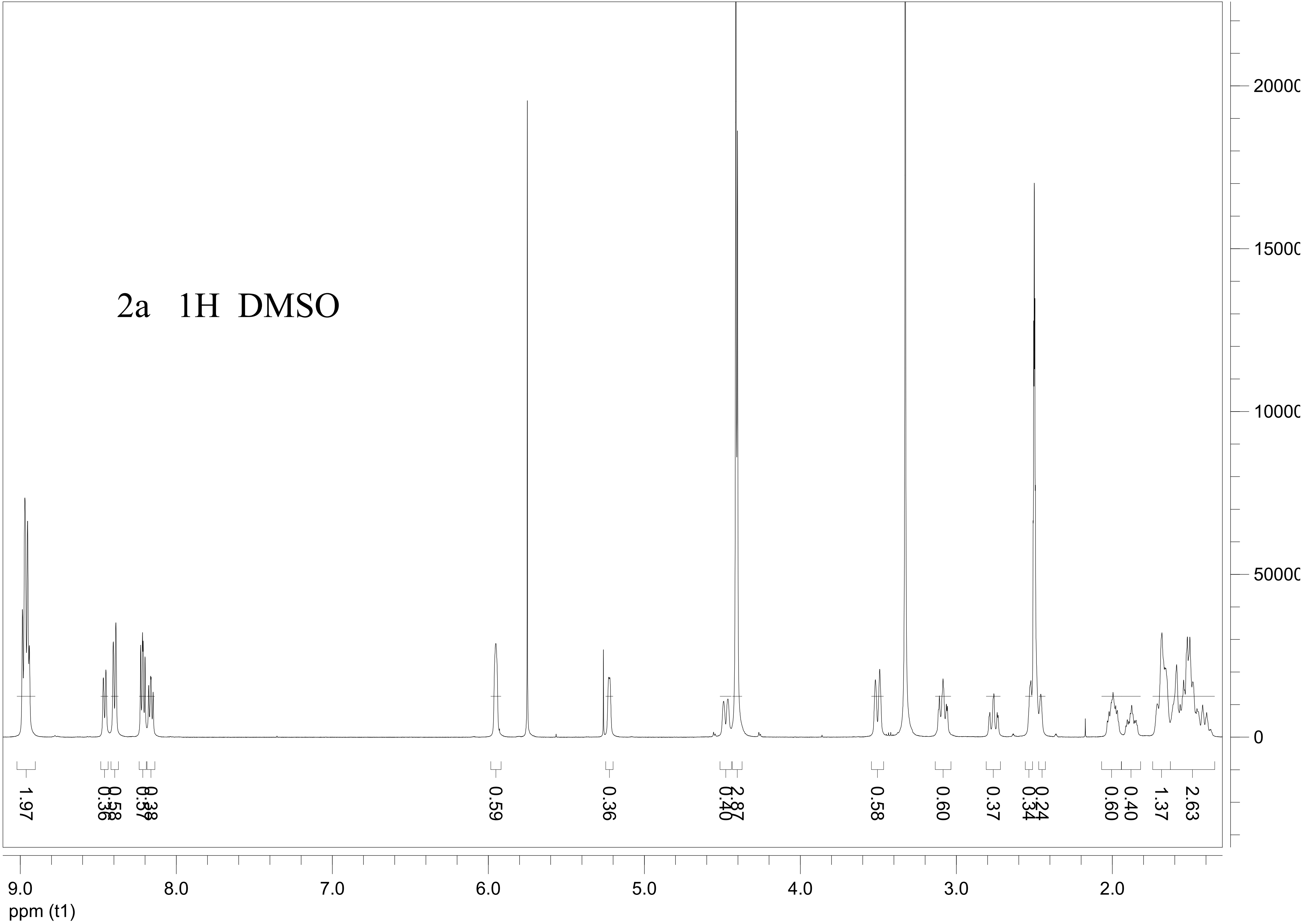

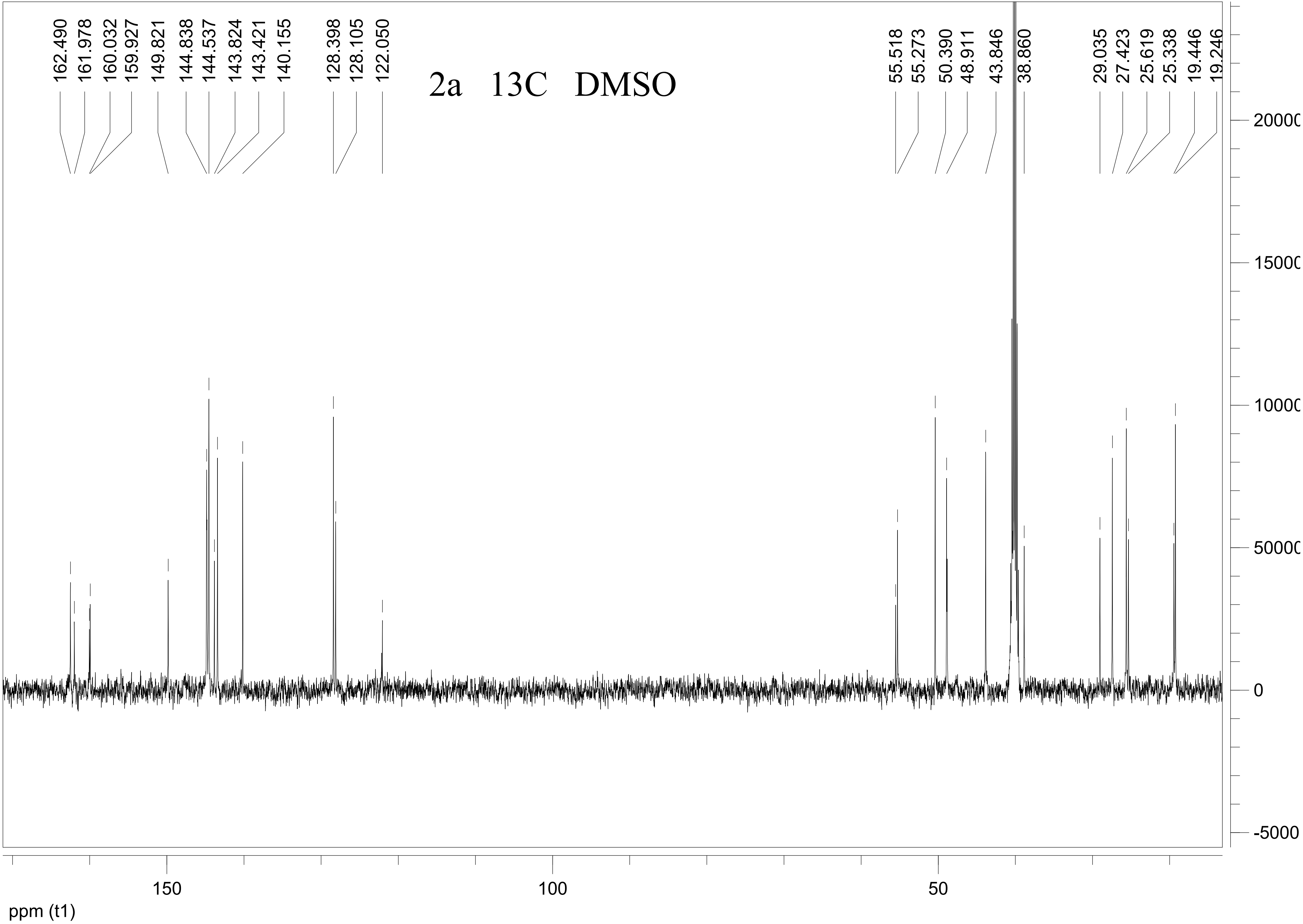
2a 13C DMSO
162.490
161.978
160.032
159.927
149.821
144.838
144.537
143.824
143.421
140.155
128.398
128.105
122.050
55.518
55.273
50.390
48.911
43.846
38.860
29.035
27.423
25.619
25.338
19.446
19.246
20000
15000
10000
50000
0
-5000
150
100
50
ppm (t1)

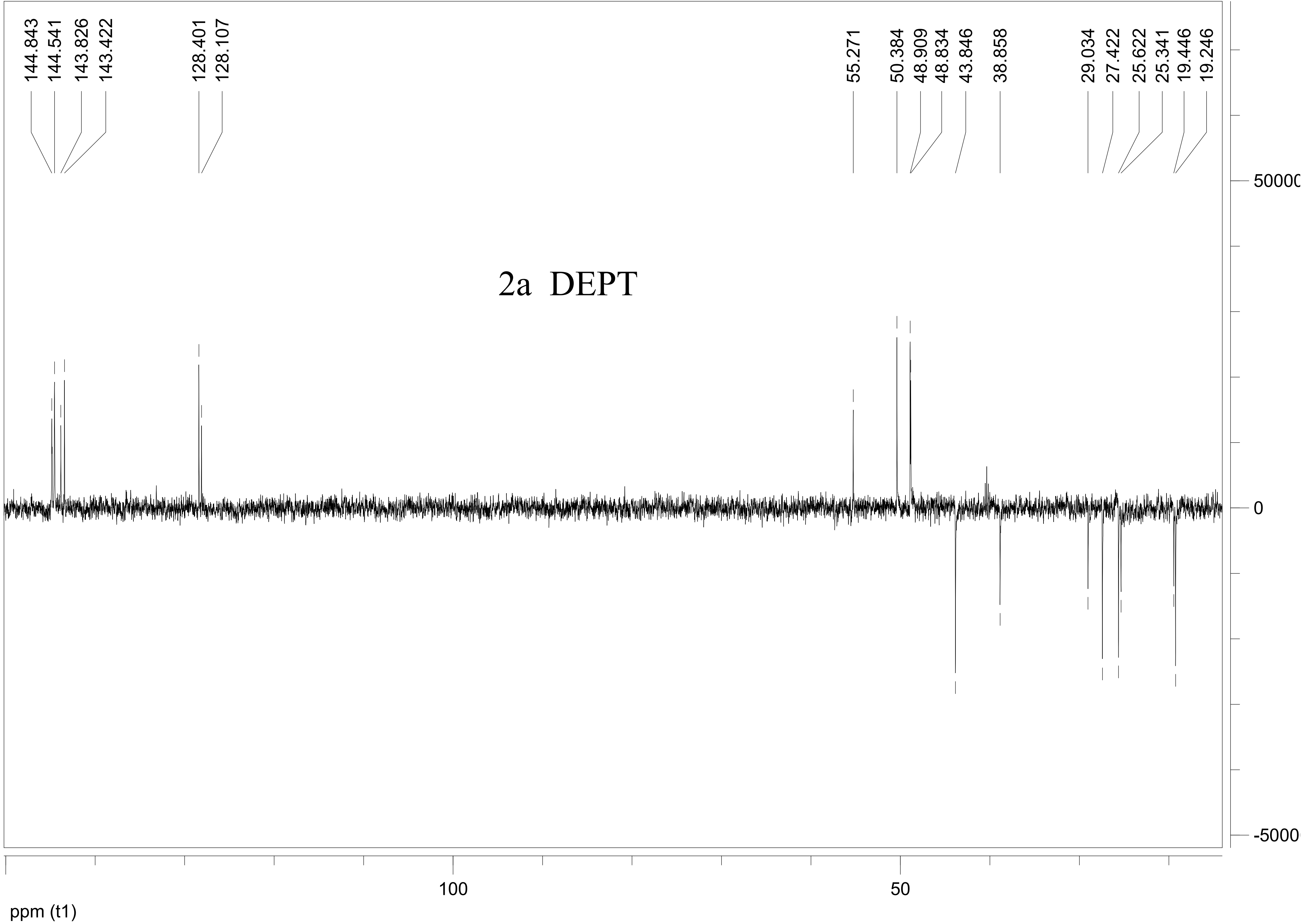
144.843
144.541
143.826
143.422
128.401
128.107
55.271
50.384
48.909
48.834
43.846
38.858
29.034
27.422
25.622
25.341
19.446
19.246
2a DEPT
50000
0
-5000
100
50
ppm (t1)

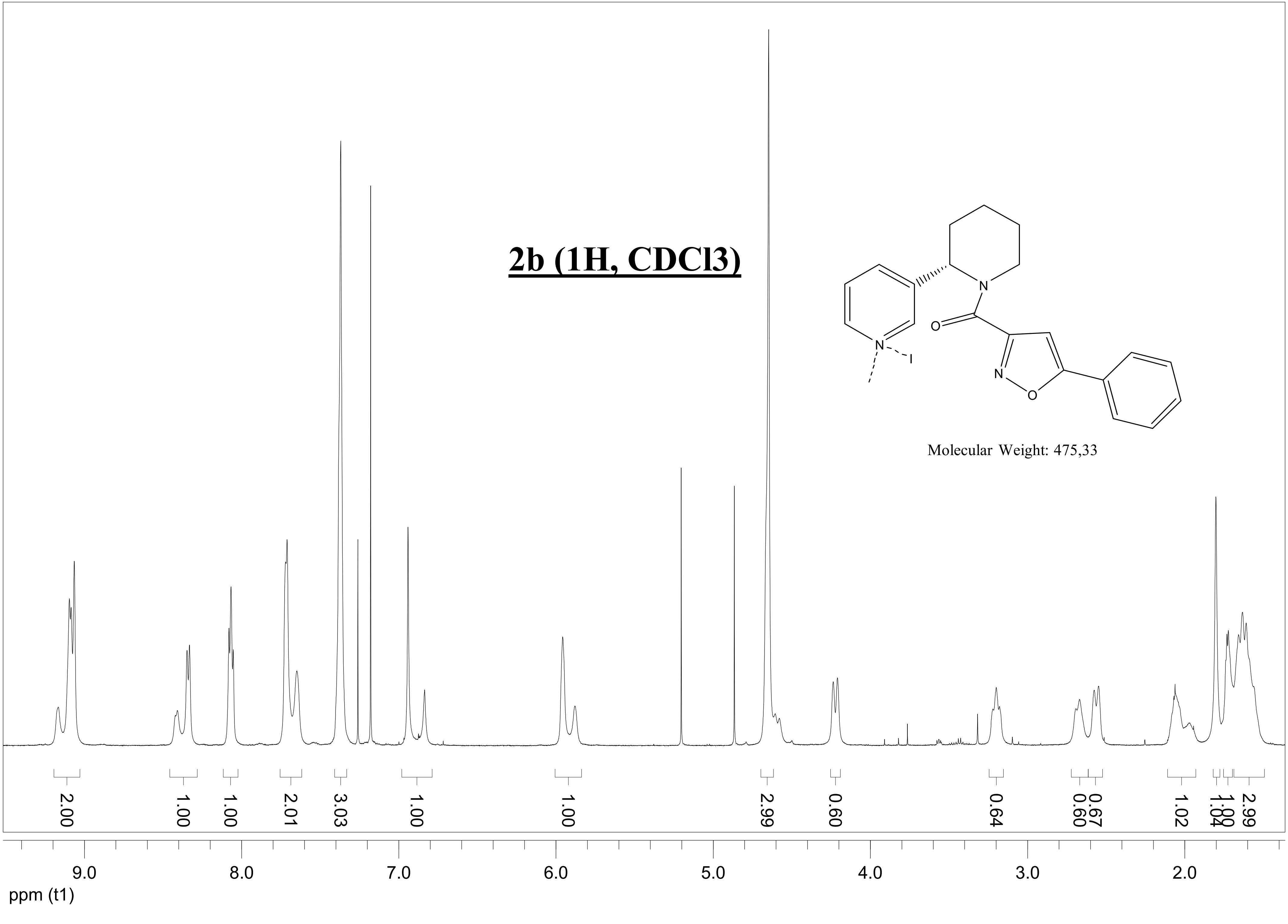
2b (1H, CDCl3)
N
I
O
N
O
Molecular Weight: 475,33
2.00
1.00
1.00
2.01
3.03
1.00
1.00
2.99
0.60
0.64
0.60
0.67
1.02
1.04
1.00
2.99
9.0
8.0
7.0
6.0
5.0
4.0
3.0
2.0
ppm (t1)

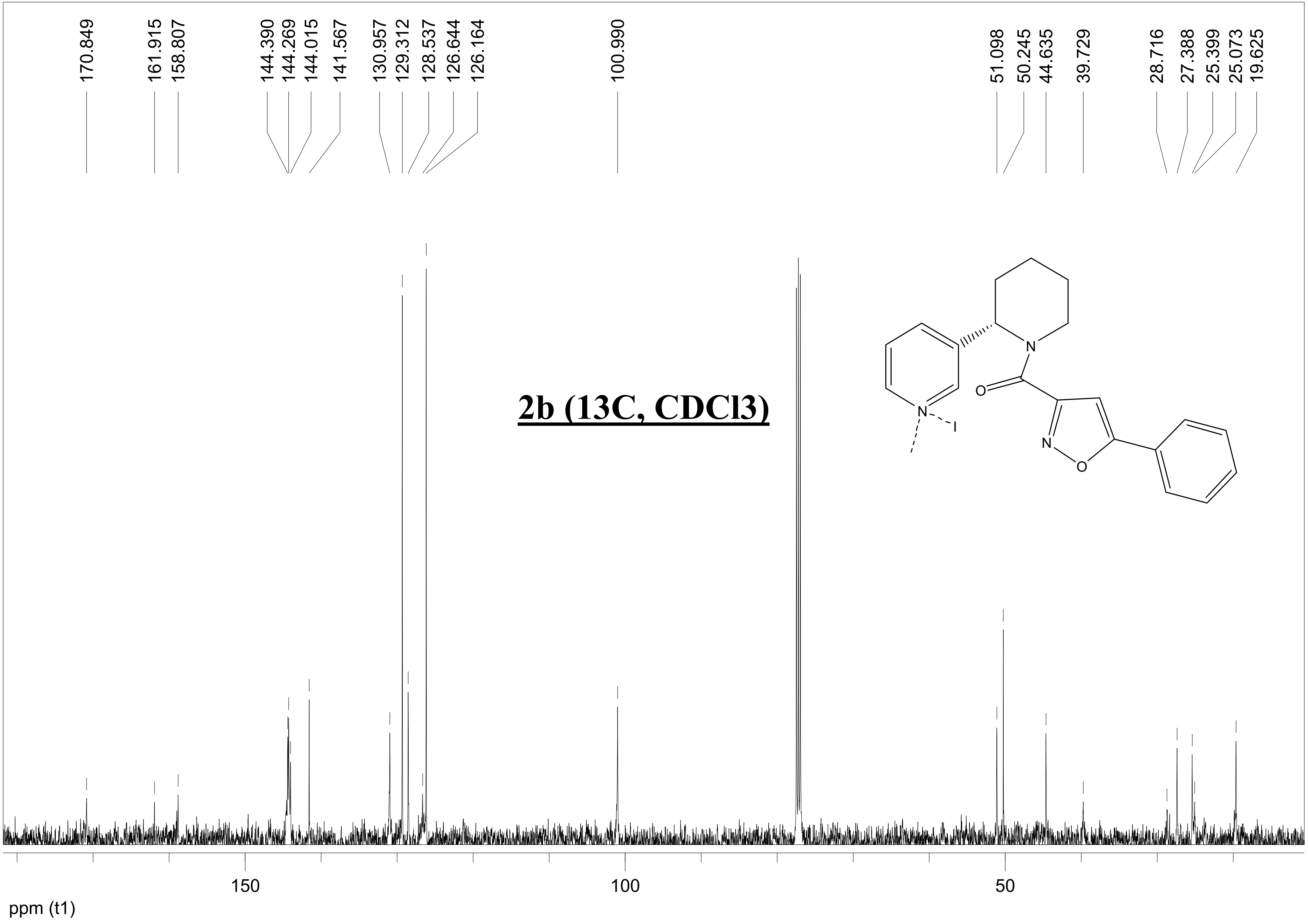
170.849
161.915
158.807
144.390
144.269
144.015
141.567
130.957
129.312
128.537
126.644
126.164
100.990
51.098
50.245
44.635
39.729
28.716
27.388
25.399
25.073
19.625
2b (13C, CDCl3)
N
I
O
N
O
N
150
100
50
ppm (t1)

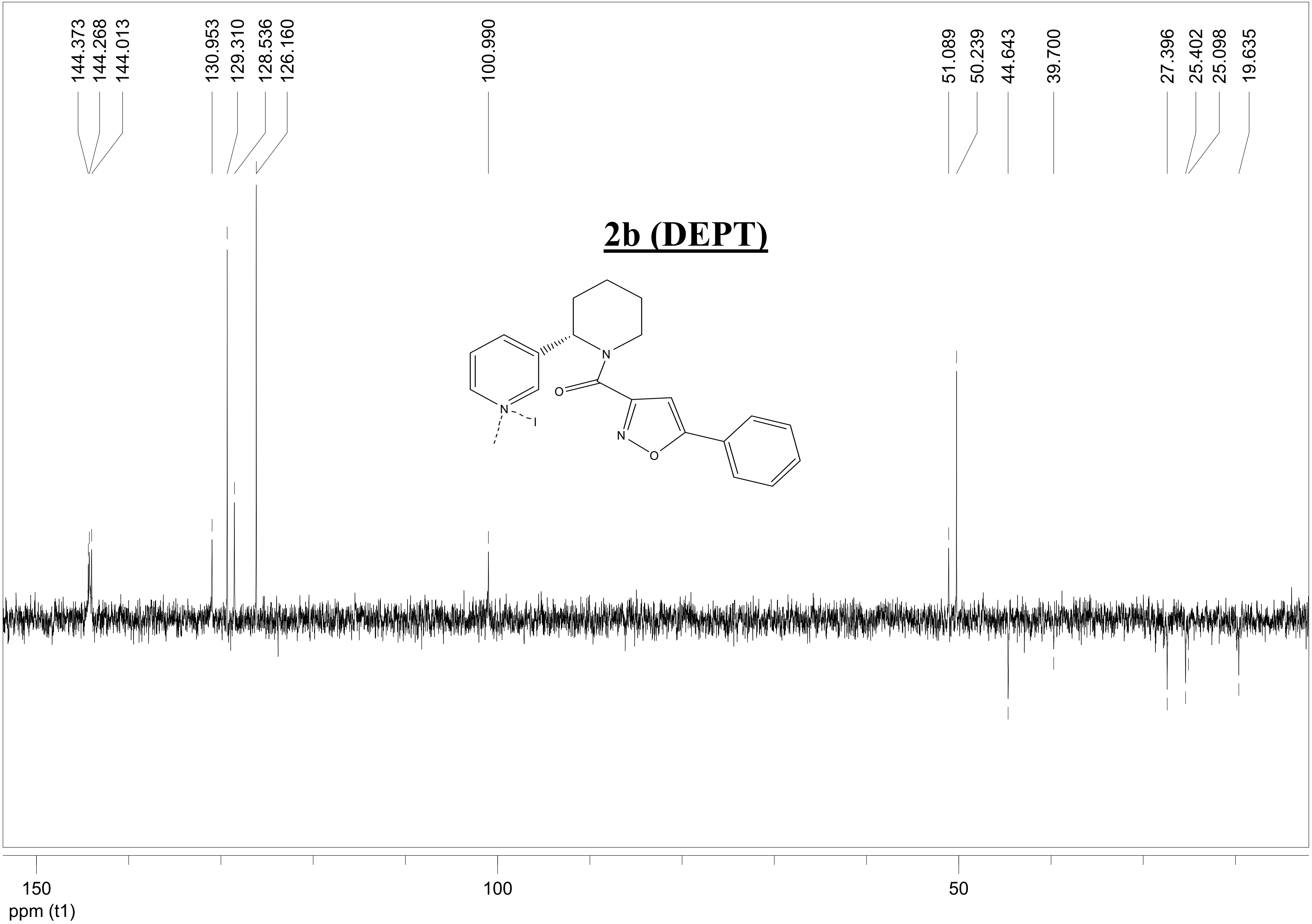
144.373
144.268
144.013
130.953
129.310
128.536
126.160
100.990
51.089
50.239
44.643
39.700
27.396
25.402
25.098
19.635
2b (DEPT)
N
O
N
O
N
I
150
100
50
ppm (t1)

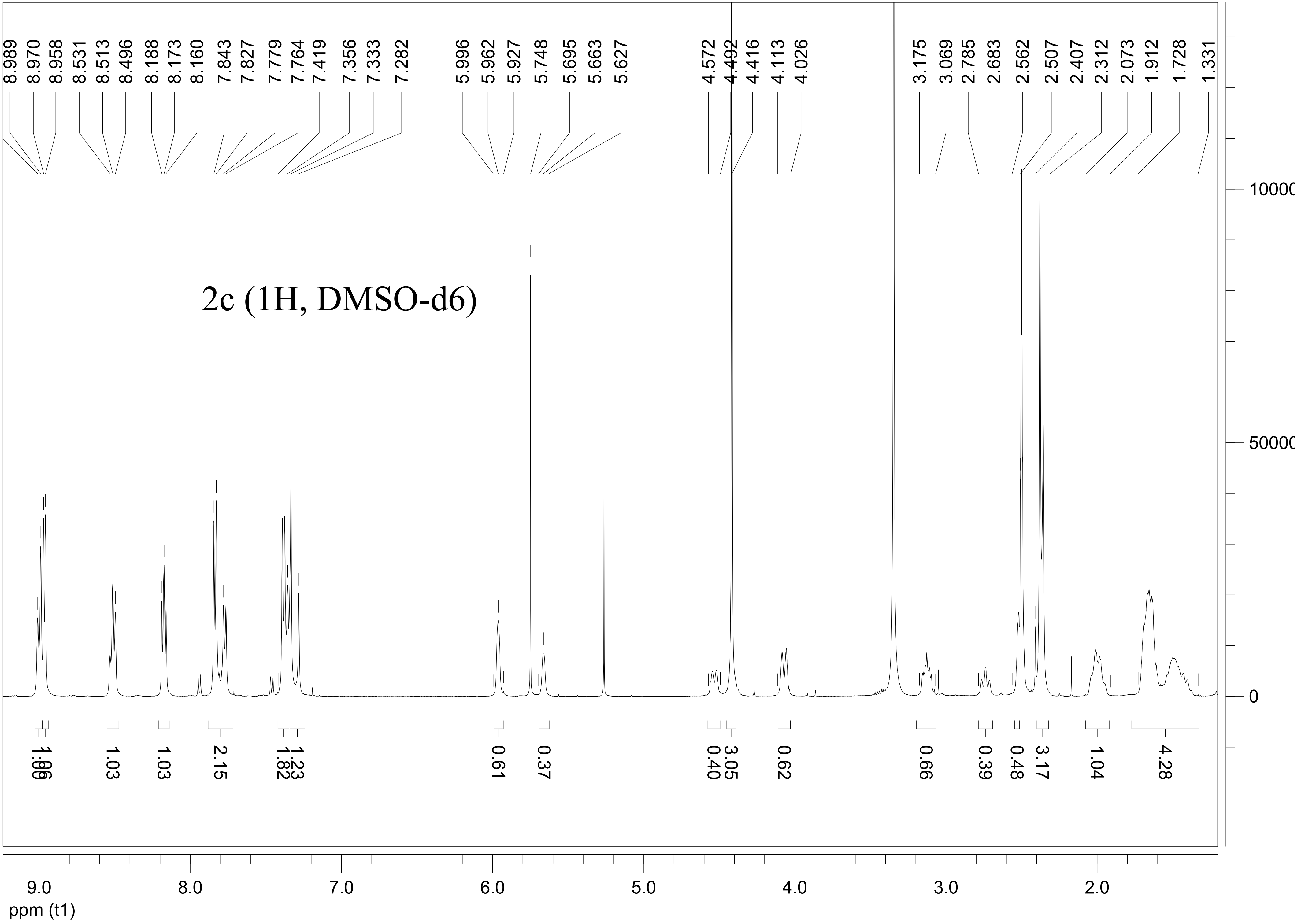
2c (1H, DMSO-d6)
8.989 8.970 8.958 8.531 8.513 8.496 8.188 8.173 8.160 7.843 7.827 7.779 7.764 7.419 7.356 7.333 7.282 5.996 5.962 5.927 5.748 5.695 5.663 5.627 4.572 4.492 4.416 4.113 4.026 3.175 3.069 2.785 2.683 2.562 2.507 2.407 2.312 2.073 1.912 1.728 1.331
1.00 1.06 1.03 1.03 2.15 1.82 1.23 0.61 0.37 0.40 3.05 0.62 0.66 0.39 0.48 3.17 1.04 4.28
9.0 8.0 7.0 6.0 5.0 4.0 3.0 2.0
ppm (t1)
0
5000C
1000C

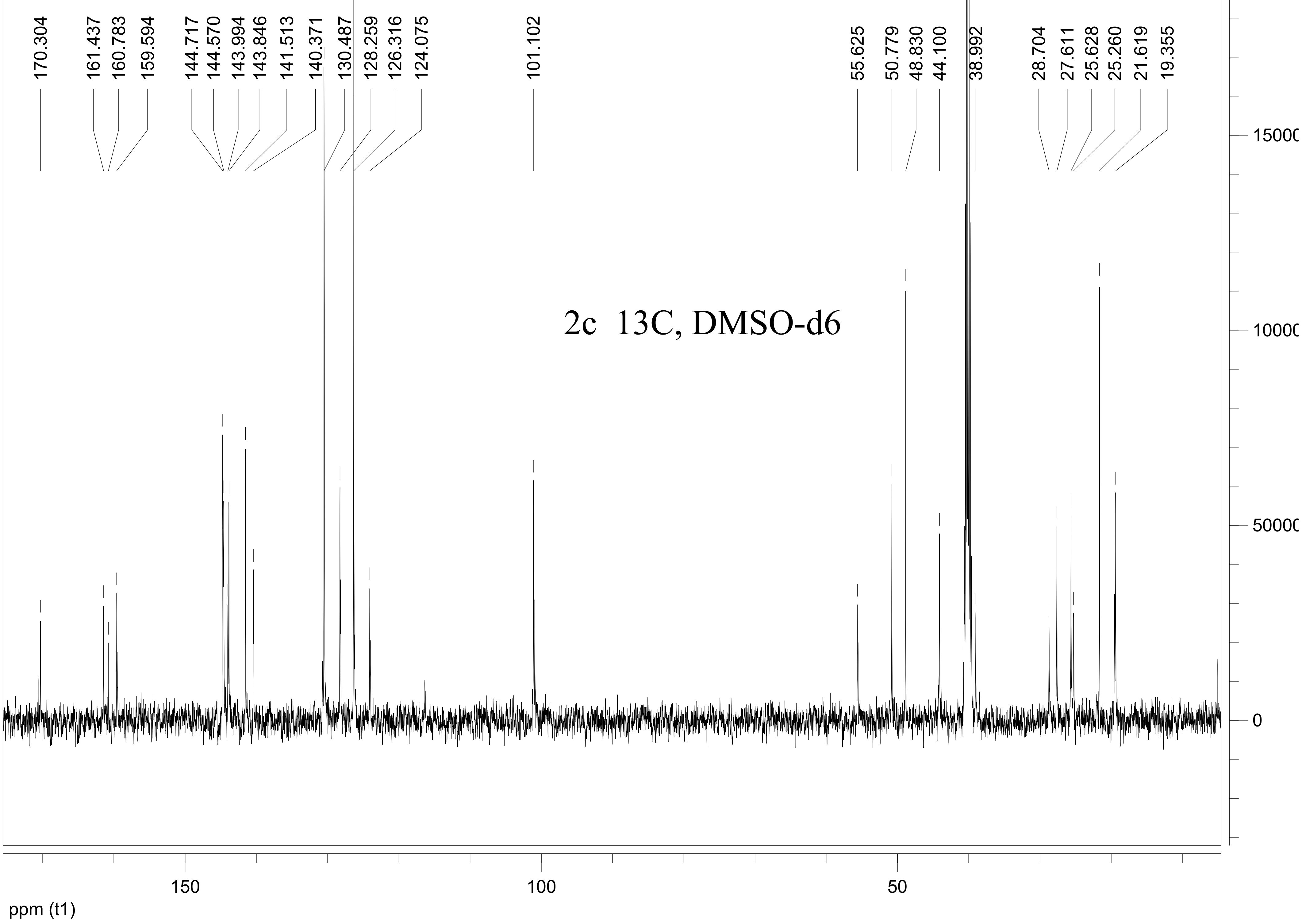
170.304
161.437
160.783
159.594
144.717
144.570
143.994
143.846
141.513
140.371
130.487
128.259
126.316
124.075
101.102
55.625
50.779
48.830
44.100
38.992
28.704
27.611
25.628
25.260
21.619
19.355
2c 13C, DMSO-d6
150000
100000
50000
0
150
100
50
ppm (t1)

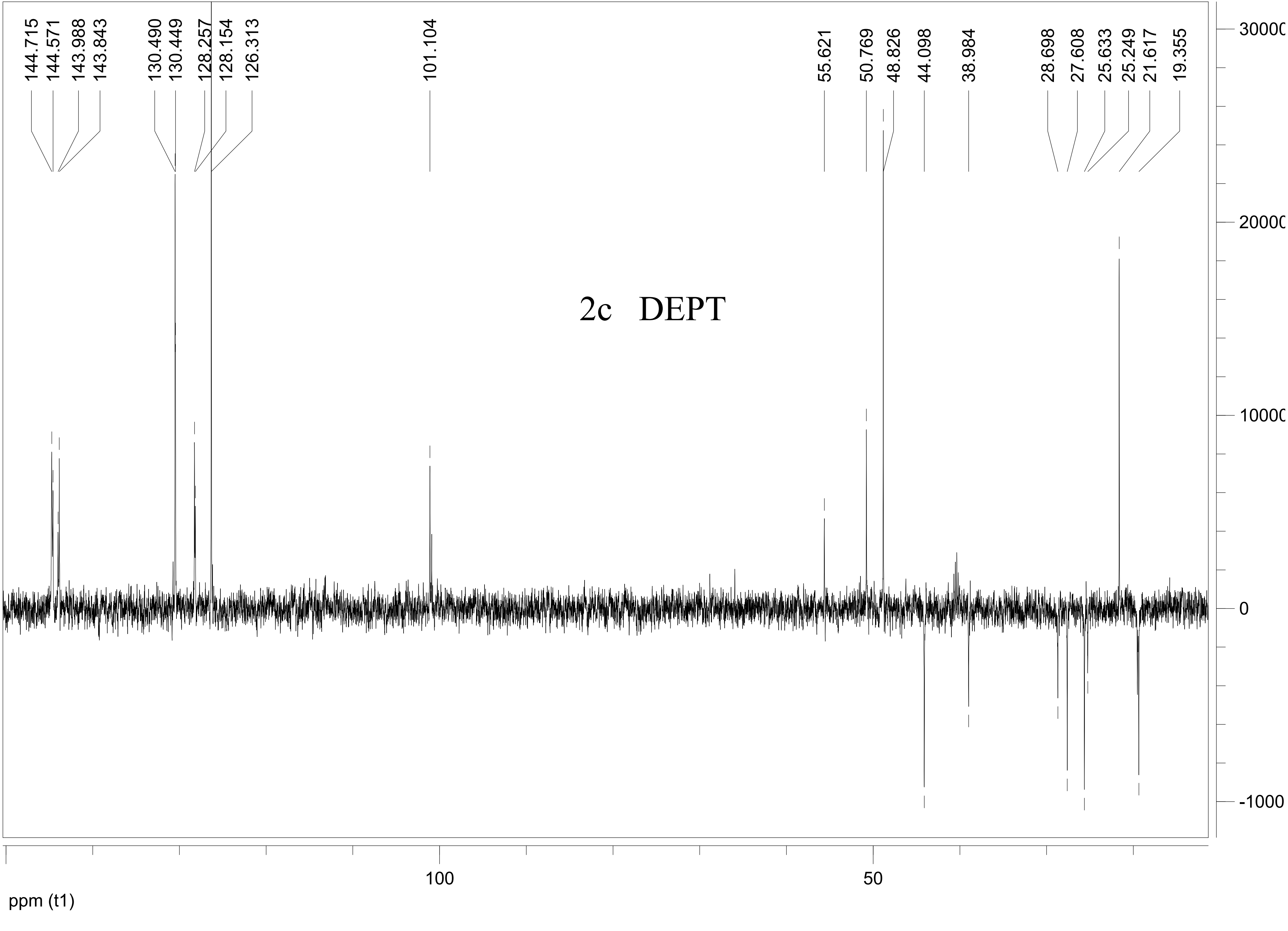

144.715
144.571
143.988
143.843
130.490
130.449
128.257
128.154
126.313
101.104
55.621
50.769
48.826
44.098
38.984
28.698
27.608
25.633
25.249
21.617
19.355
2c DEPT
30000
20000
10000
0
-1000
100
50
ppm (t1)

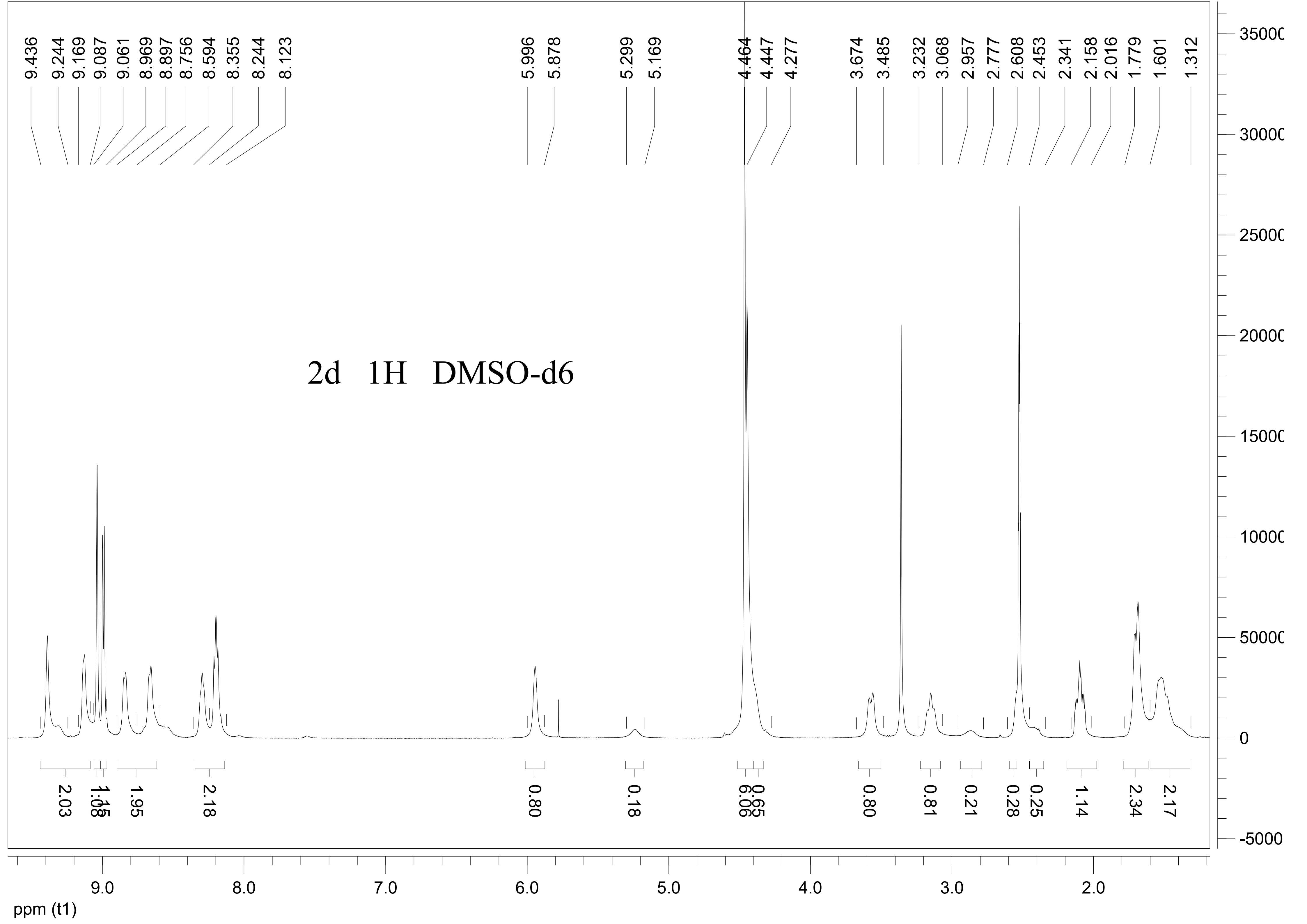
2d 1H DMSO-d6
9.436 9.244 9.169 9.087 9.061 8.969 8.897 8.756 8.594 8.355 8.244 8.123 5.996 5.878 5.299 5.169 4.464 4.447 4.277 3.674 3.485 3.232 3.068 2.957 2.777 2.608 2.453 2.341 2.158 2.016 1.779 1.601 1.312
2.03 1.08 1.15 1.95 2.18 0.80 0.18 6.06 0.65 0.80 0.81 0.21 0.28 0.25 1.14 2.34 2.17
ppm (t1)

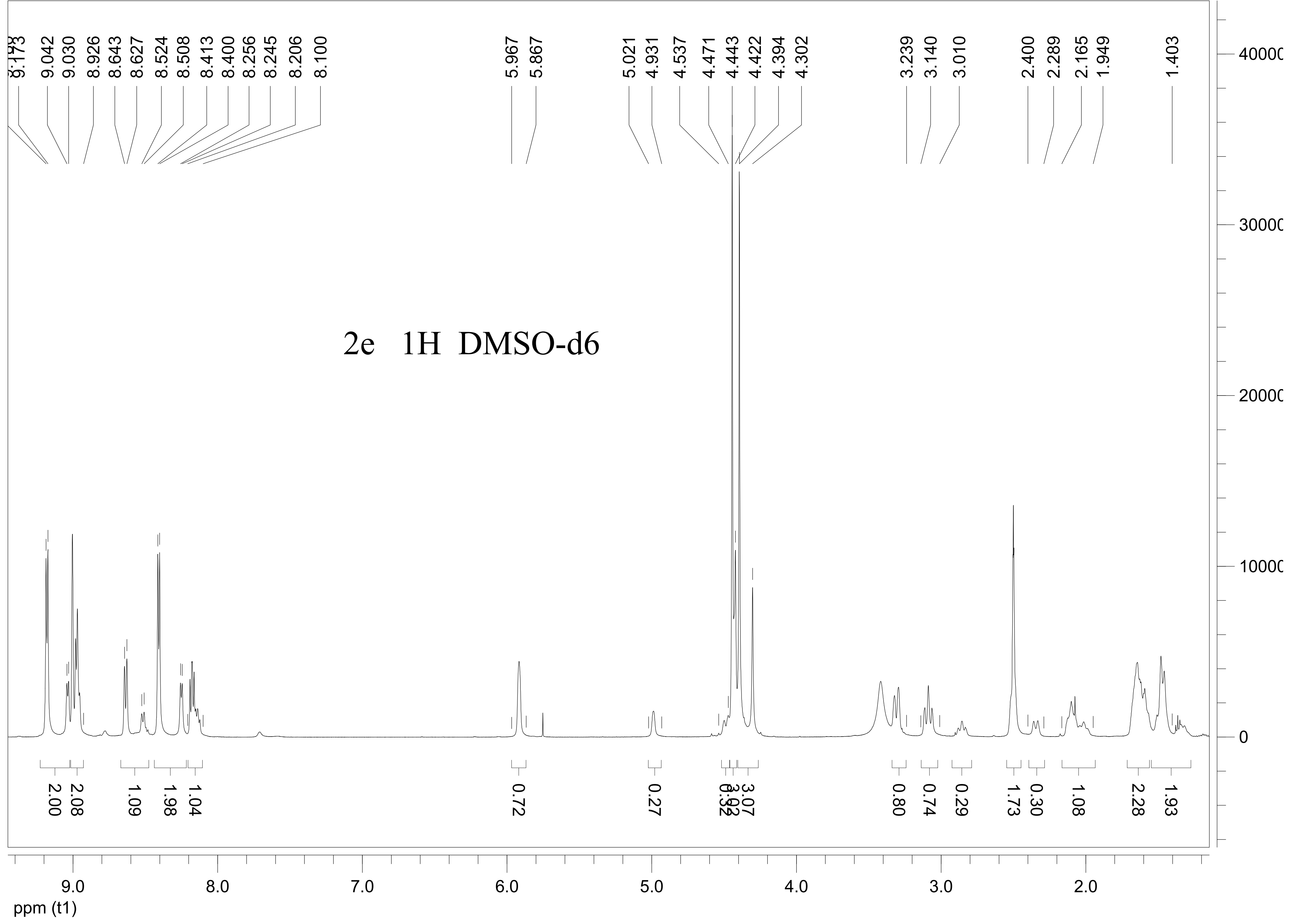
2e 1H DMSO-d6
9.173
9.042
9.030
8.926
8.643
8.627
8.524
8.508
8.413
8.400
8.256
8.245
8.206
8.100
5.967
5.867
5.021
4.931
4.537
4.471
4.443
4.422
4.394
4.302
3.239
3.140
3.010
2.400
2.289
2.165
1.949
1.403
2.00
2.08
1.09
1.98
1.04
0.72
0.27
3.07
0.80
0.74
0.29
1.73
0.30
1.08
2.28
1.93
9.0
8.0
7.0
6.0
5.0
4.0
3.0
2.0
ppm (t1)

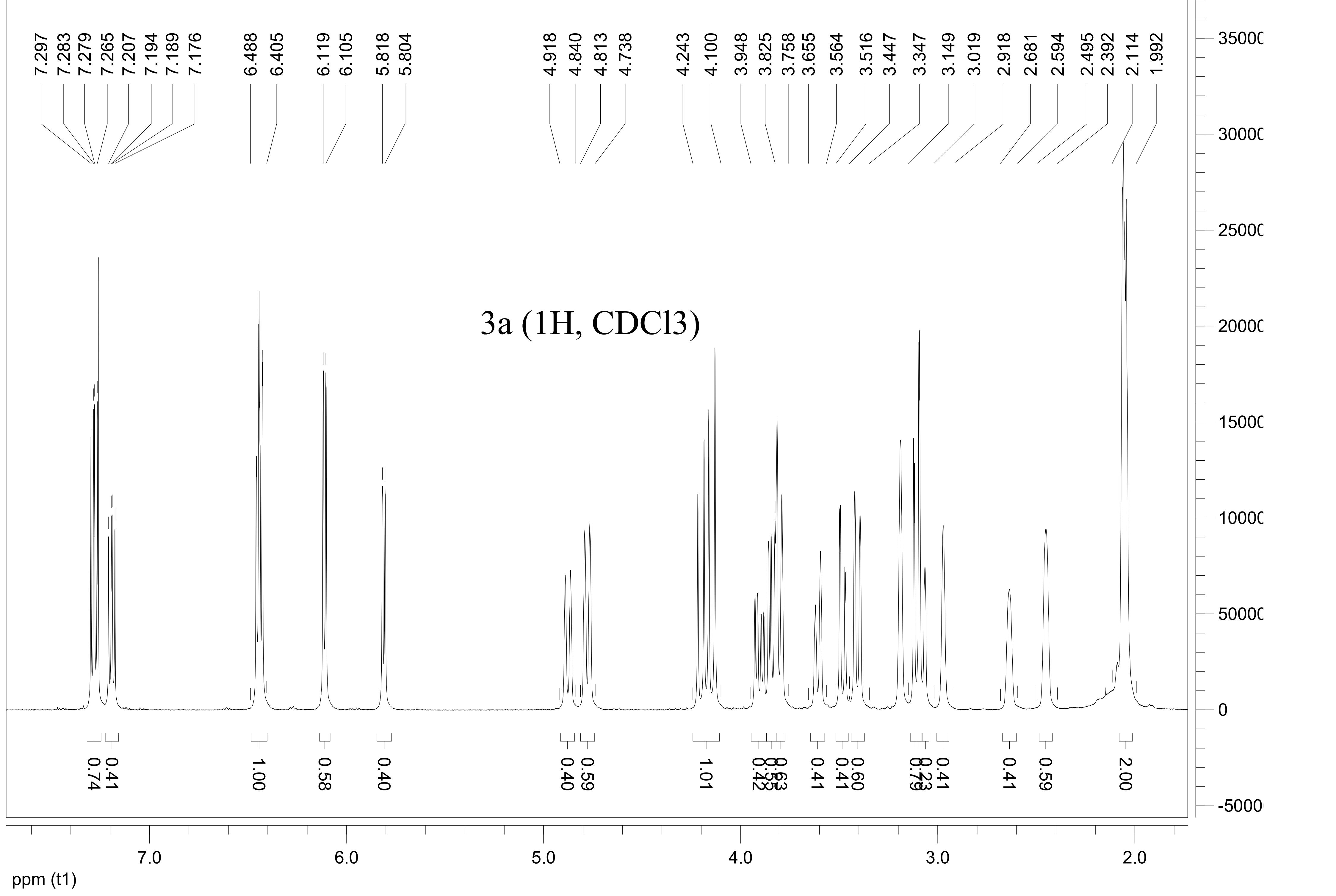

3a (1H, CDCl3)
ppm (t1)

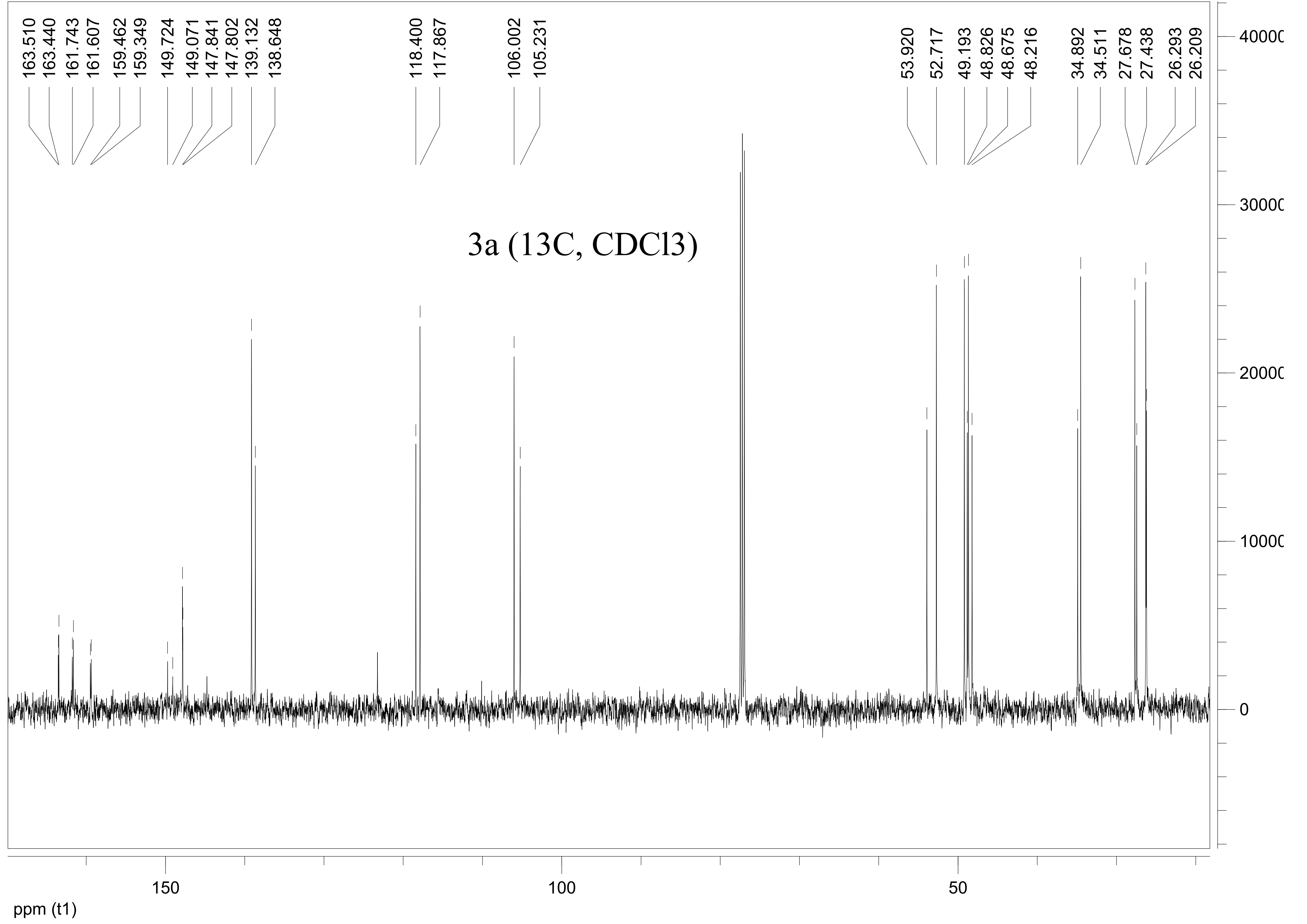

163.510
163.440
161.743
161.607
159.462
159.349
149.724
149.071
147.841
147.802
139.132
138.648
118.400
117.867
106.002
105.231
53.920
52.717
49.193
48.826
48.675
48.216
34.892
34.511
27.678
27.438
26.293
26.209
3a (13C, CDCl3)
40000
30000
20000
10000
0
150
100
50
ppm (t1)

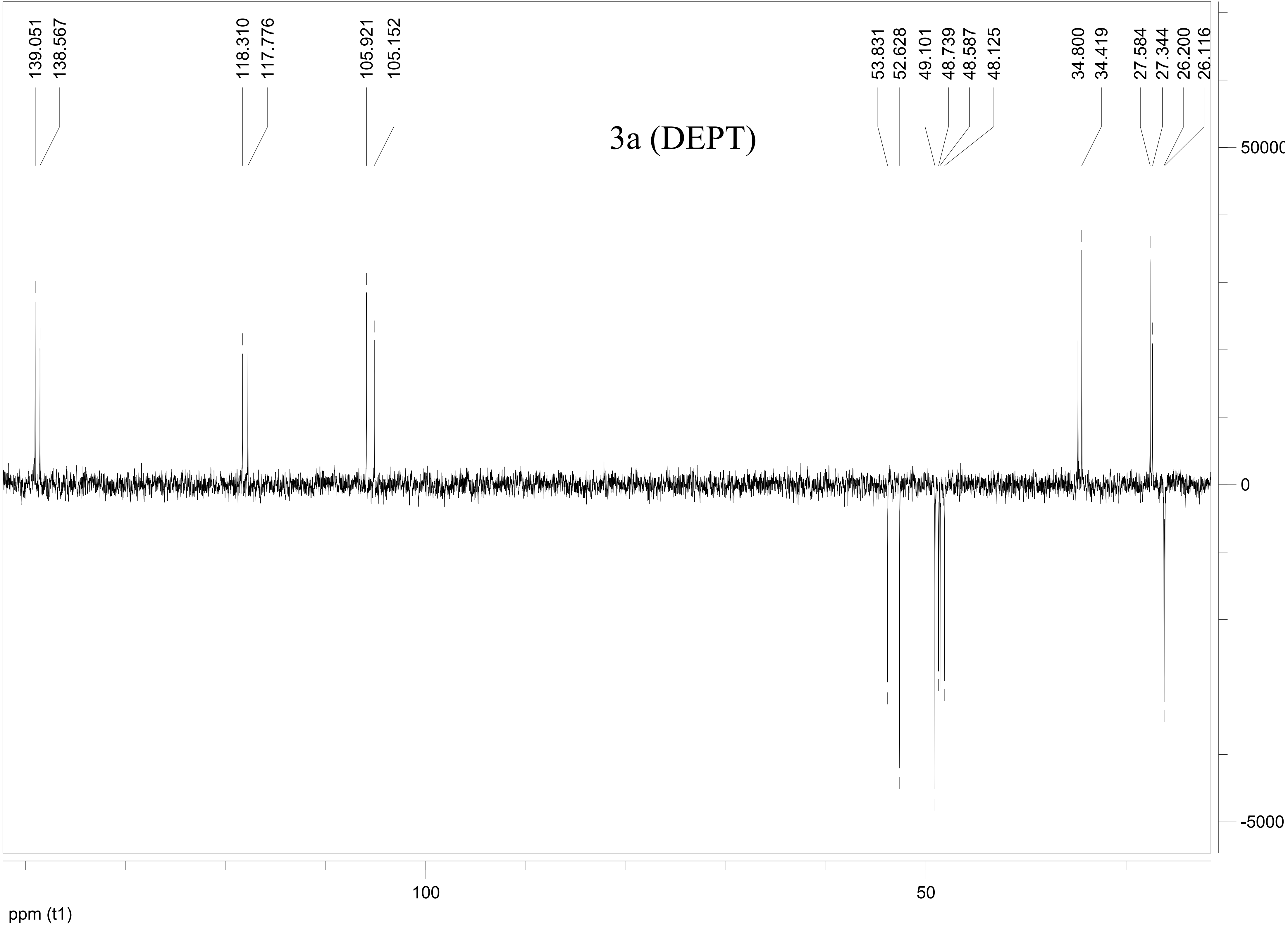
139.051
138.567
118.310
117.776
105.921
105.152
53.831
52.628
49.101
48.739
48.587
48.125
34.800
34.419
27.584
27.344
26.200
26.116
3a (DEPT)
50000
0
-5000
100
50
ppm (t1)

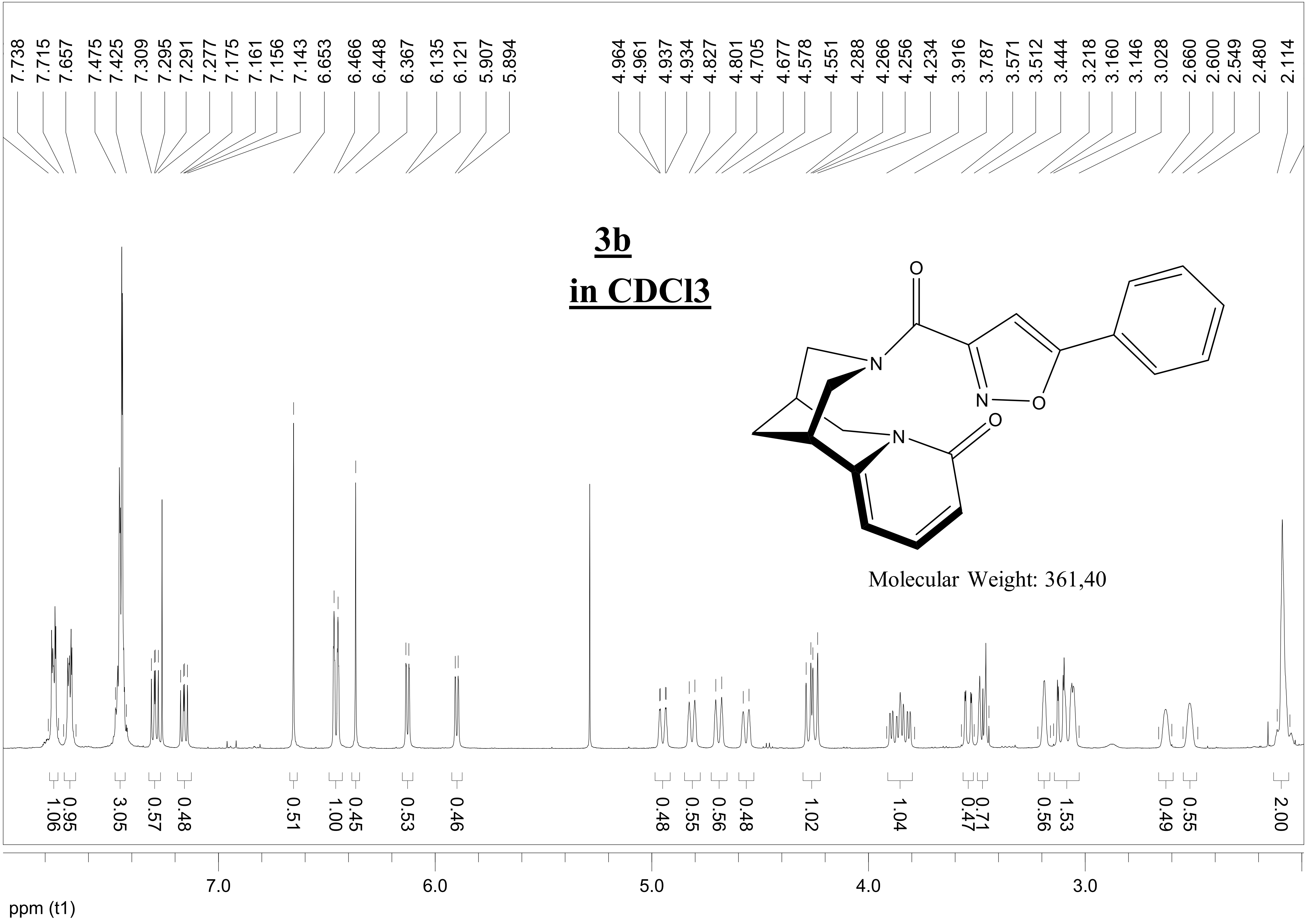
3b
in CDCl3
Molecular Weight: 361,40
O
N
N
O
O
N
7.738 7.715 7.657 7.475 7.425 7.309 7.295 7.291 7.277 7.175 7.161 7.156 7.143 6.653 6.466 6.448 6.367 6.135 6.121 5.907 5.894
4.964 4.961 4.937 4.934 4.827 4.801 4.705 4.677 4.578 4.551 4.288 4.266 4.256 4.234 3.916 3.787 3.571 3.512 3.444 3.218 3.160 3.146 3.028 2.660 2.600 2.549 2.480 2.114
1.06 0.95 3.05 0.57 0.48 0.51 1.00 0.45 0.53 0.46 0.48 0.55 0.56 0.48 1.02 1.04 0.47 0.71 0.56 1.53 0.49 0.55 2.00
7.0
6.0
5.0
4.0
3.0
ppm (t1)

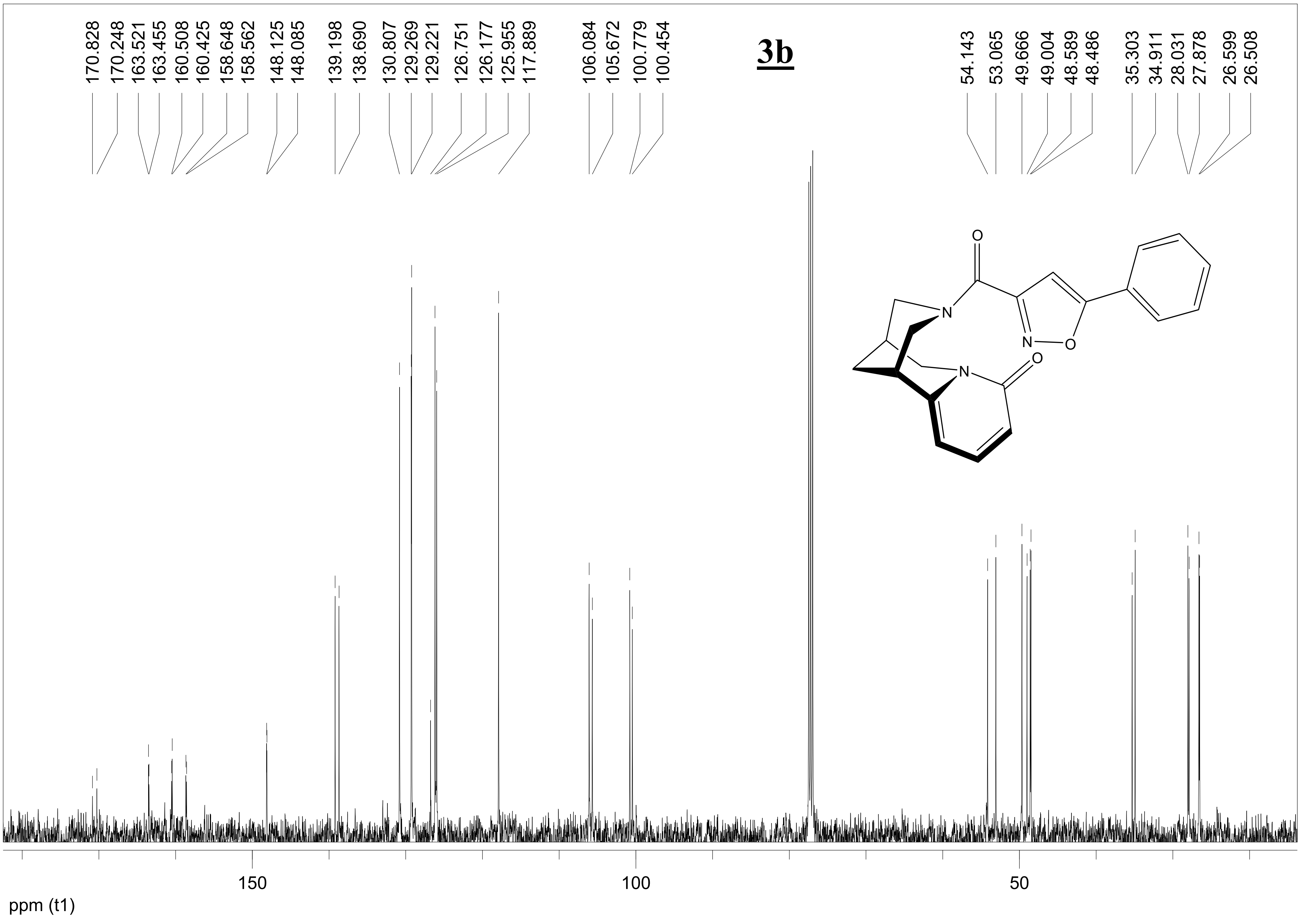
170.828
170.248
163.521
163.455
160.508
160.425
158.648
158.562
148.125
148.085
139.198
138.690
130.807
129.269
129.221
126.751
126.177
125.955
117.889
106.084
105.672
100.779
100.454
3b
54.143
53.065
49.666
49.004
48.589
48.486
35.303
34.911
28.031
27.878
26.599
26.508
O
N
N
O
N
O
150
100
50
ppm (t1)

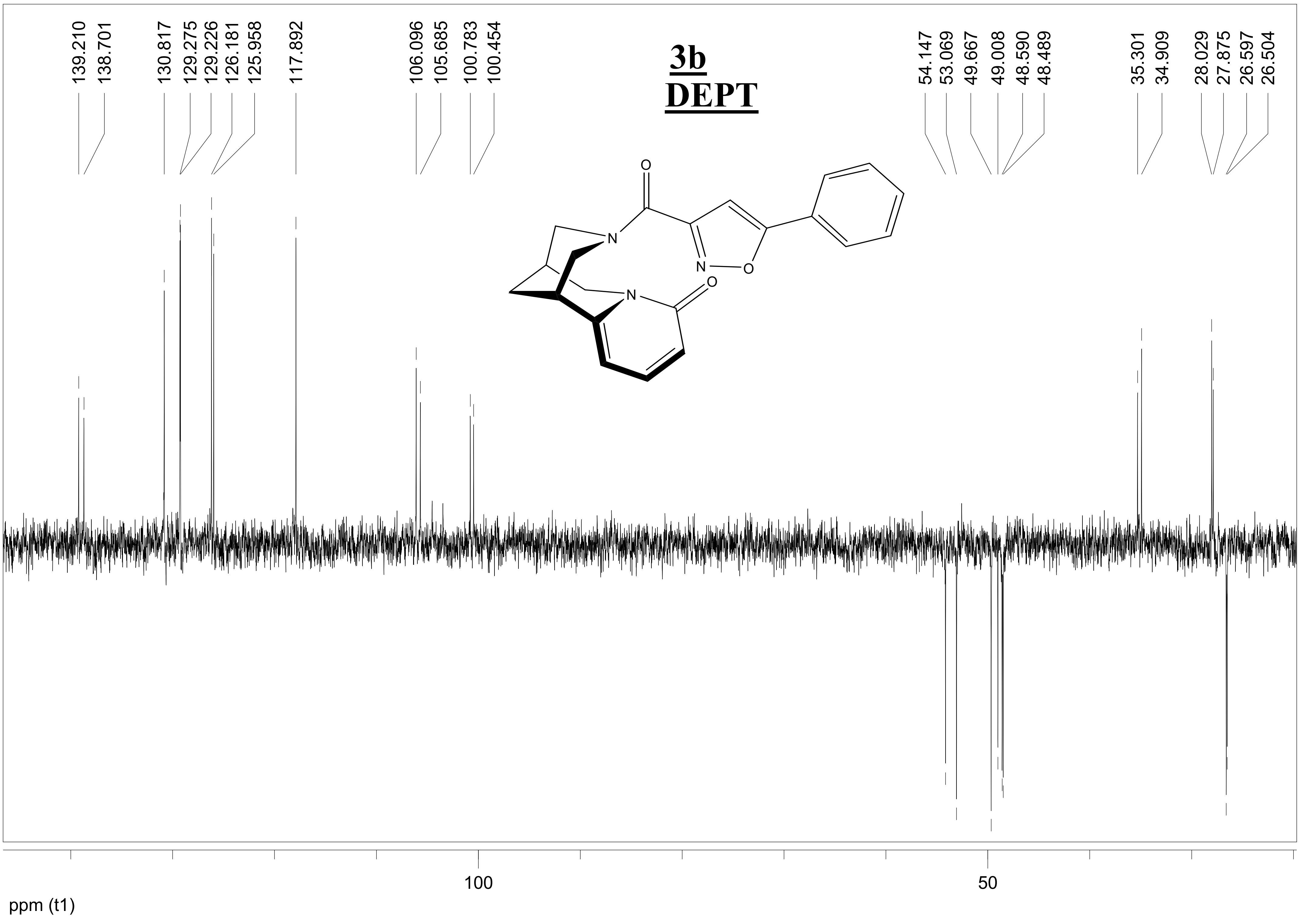
139.210
138.701
130.817
129.275
129.226
126.181
125.958
117.892
106.096
105.685
100.783
100.454
3b
DEPT
54.147
53.069
49.667
49.008
48.590
48.489
35.301
34.909
28.029
27.875
26.597
26.504
O
N
N
O
N
O
100
50
ppm (t1)

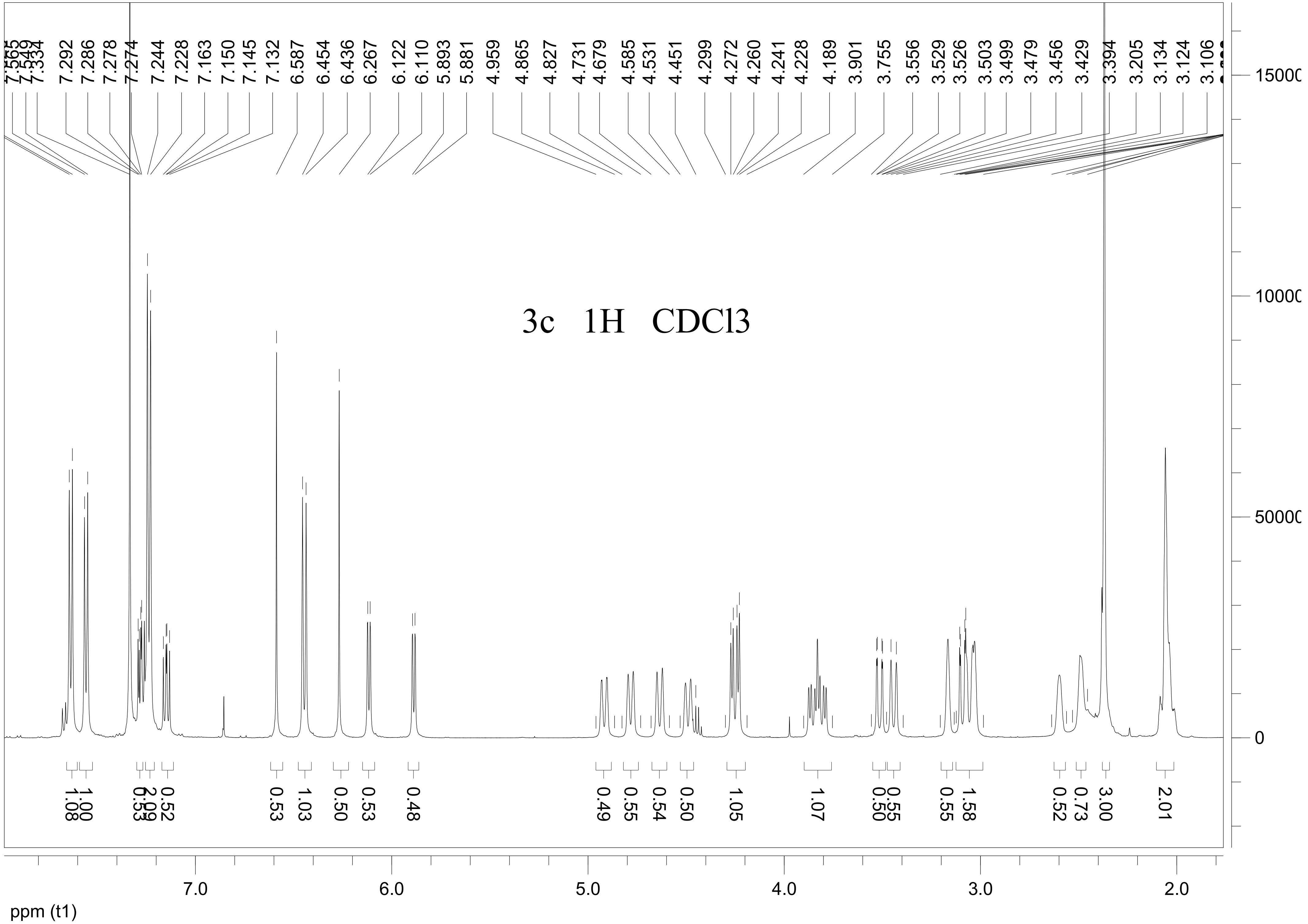
3c 1H CDCl3
ppm (t1)
7.0
6.0
5.0
4.0
3.0
2.0
1.08 1.00 0.53 2.09 0.52 0.53 1.03 0.50 0.53 0.48 0.49 0.55 0.54 0.50 1.05 1.07 0.50 0.55 0.55 1.58 0.52 0.73 3.00 2.01
7.292 7.286 7.278 7.274 7.244 7.228 7.163 7.150 7.145 7.132 6.587 6.454 6.436 6.267 6.122 6.110 5.893 5.881 4.959 4.865 4.827 4.731 4.679 4.585 4.531 4.451 4.299 4.272 4.260 4.241 4.228 4.189 3.901 3.755 3.556 3.529 3.526 3.503 3.499 3.479 3.456 3.429 3.394 3.205 3.134 3.124 3.106

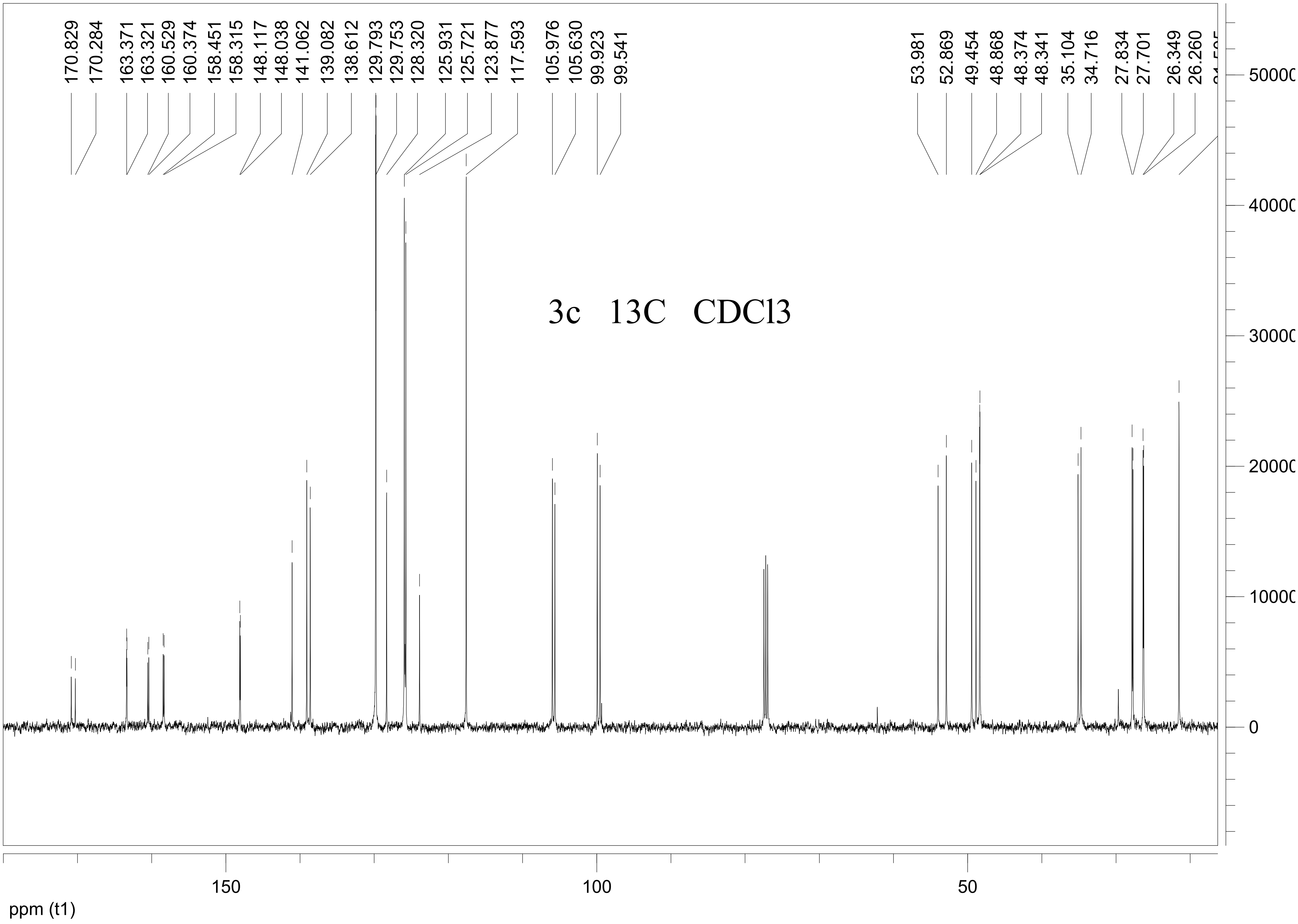
170.829
170.284
163.371
163.321
160.529
160.374
158.451
158.315
148.117
148.038
141.062
139.082
138.612
129.793
129.753
128.320
125.931
125.721
123.877
117.593
105.976
105.630
99.923
99.541
53.981
52.869
49.454
48.868
48.374
48.341
35.104
34.716
27.834
27.701
26.349
26.260
3c 13C CDCl3
50000
40000
30000
20000
10000
0
150
100
50
ppm (t1)

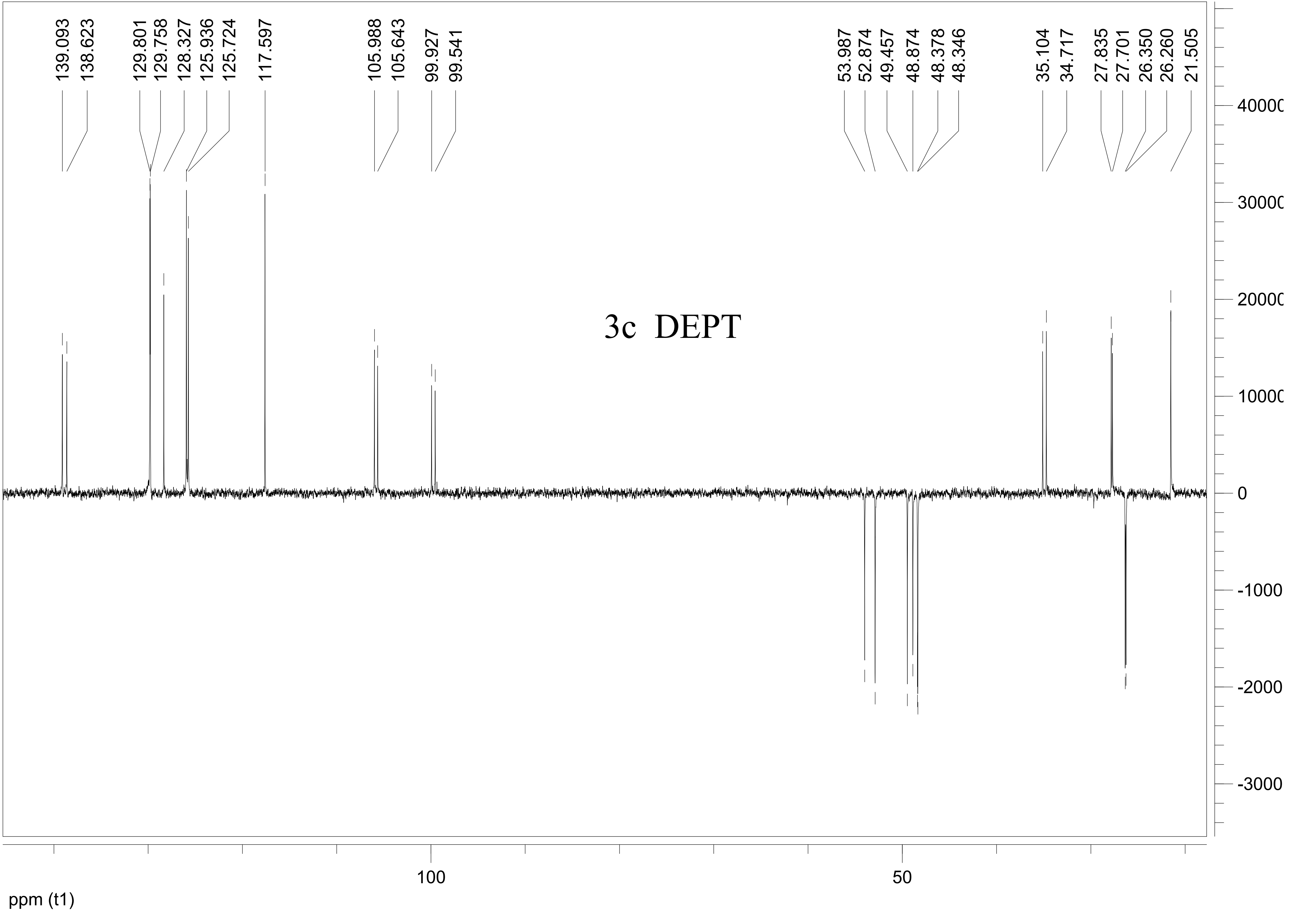

139.093
138.623
129.801
129.758
128.327
125.936
125.724
117.597
105.988
105.643
99.927
99.541
53.987
52.874
49.457
48.874
48.378
48.346
35.104
34.717
27.835
27.701
26.350
26.260
21.505
3c DEPT
100
50
ppm (t1)

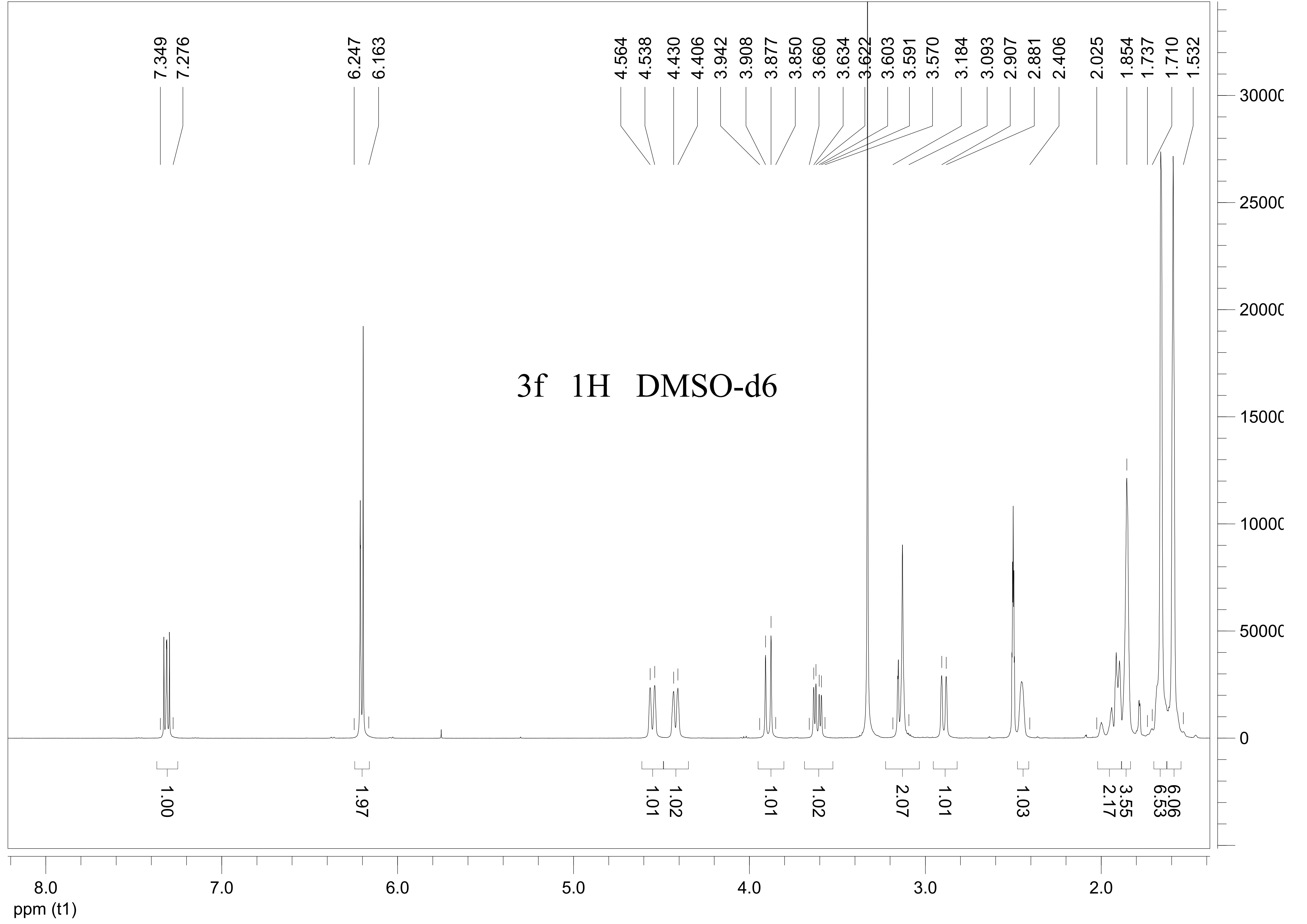

7.349
7.276
6.247
6.163
4.564
4.538
4.430
4.406
3.942
3.908
3.877
3.850
3.660
3.634
3.622
3.603
3.591
3.570
3.184
3.093
2.907
2.881
2.406
2.025
1.854
1.737
1.710
1.532
3f 1H DMSO-d6
1.00
1.97
1.01
1.02
1.01
1.02
2.07
1.01
1.03
2.17
3.55
6.53
6.06
8.0
7.0
6.0
5.0
4.0
3.0
2.0
ppm (t1)

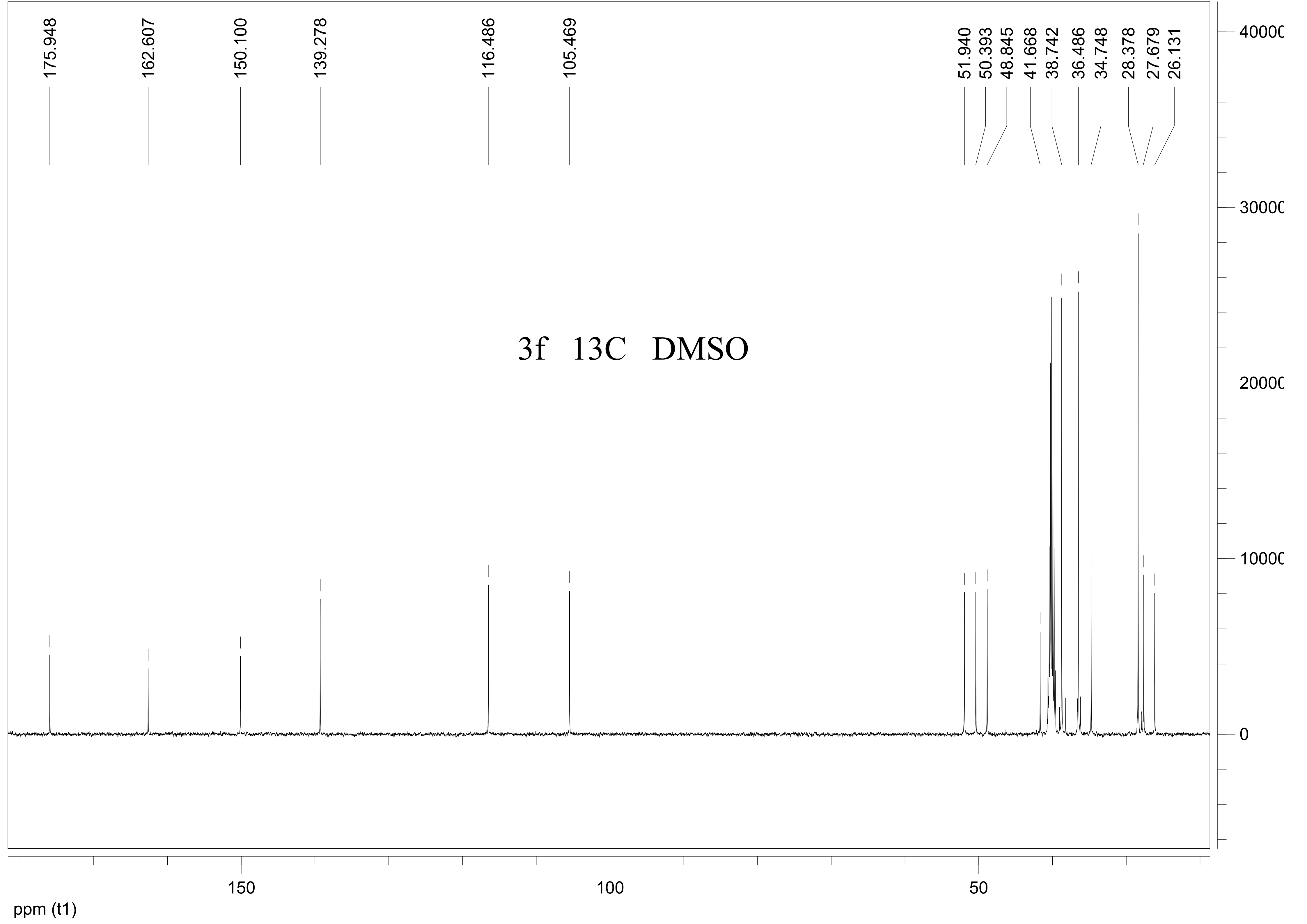

175.948
162.607
150.100
139.278
116.486
105.469
51.940
50.393
48.845
41.668
38.742
36.486
34.748
28.378
27.679
26.131
3f 13C DMSO
40000
30000
20000
10000
0
150
100
50
ppm (t1)

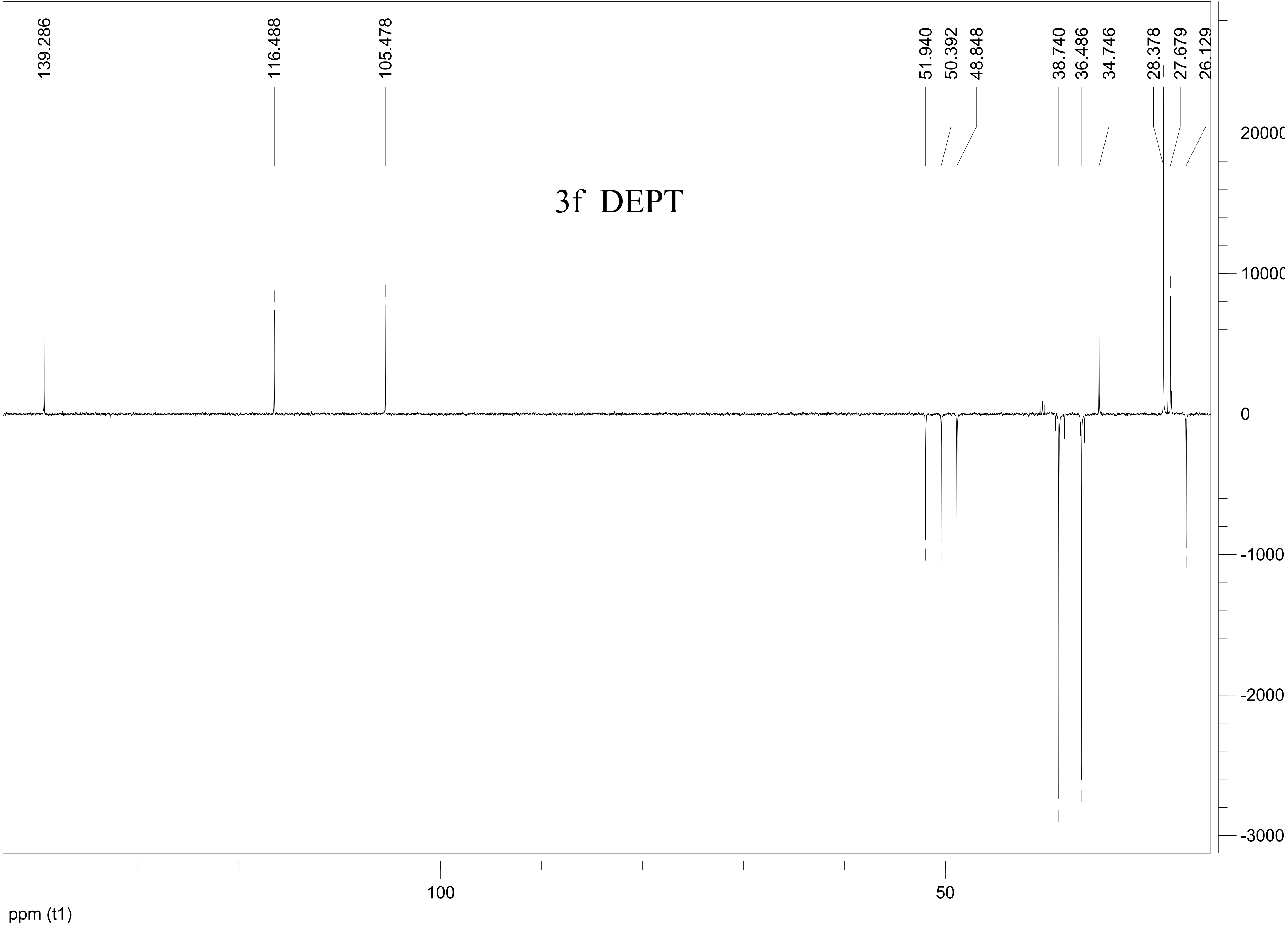
3f DEPT
139.286
116.488
105.478
51.940
50.392
48.848
38.740
36.486
34.746
28.378
27.679
26.129
20000
10000
0
-1000
-2000
-3000
100
50
ppm (t1)
