# Supplementary figures and images for: Synthesis and Biological Activity of N-acyl Anabasine and Cytisine Derivatives with Adamantane, Pyridine and 1,2-Azole Fragments
Source: Molecules. 2022 Oct 31;27(21):7387. doi: 10.3390/molecules27217387 (PMC9656753; doi:10.3390/molecules27217387)

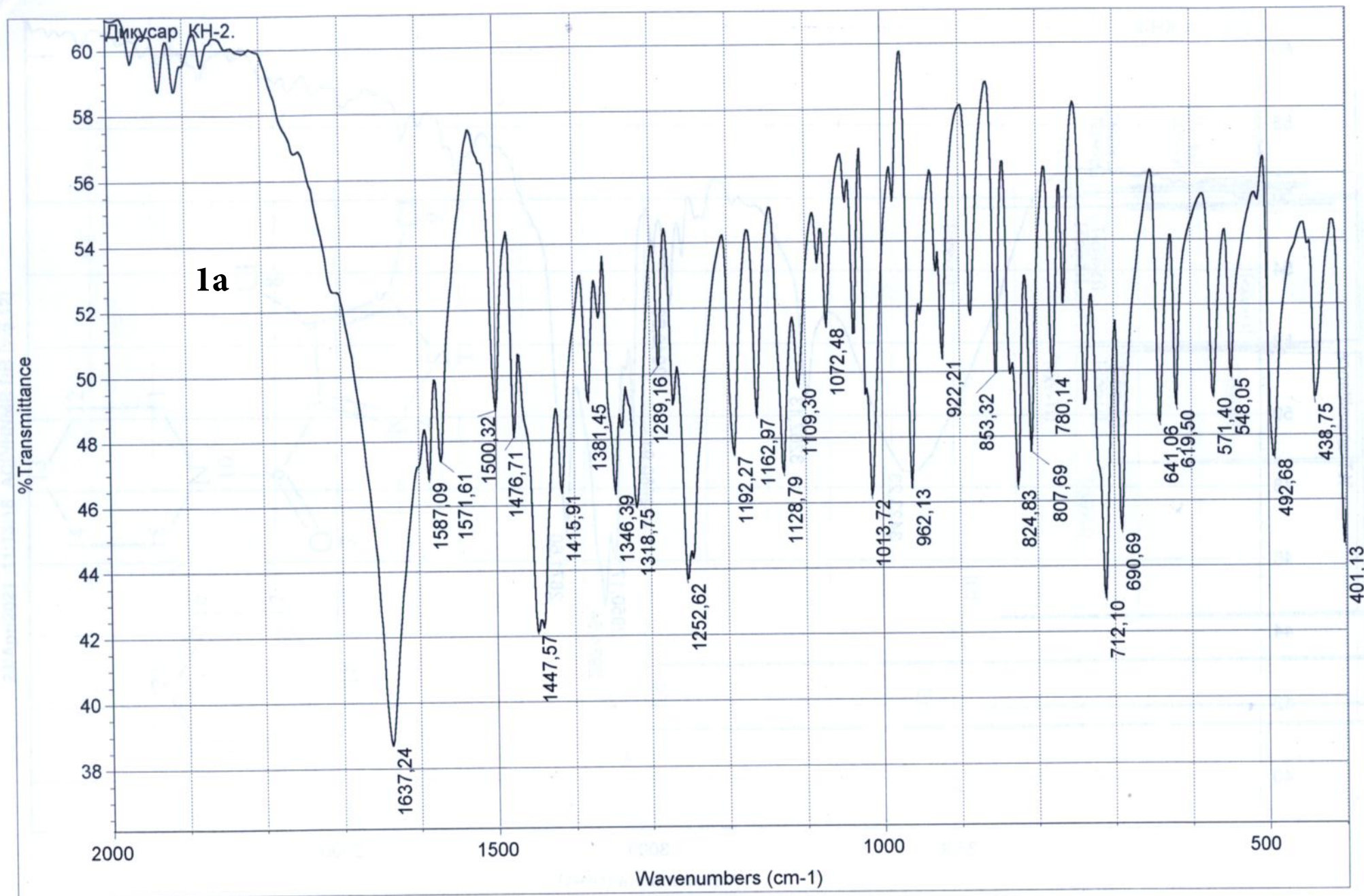

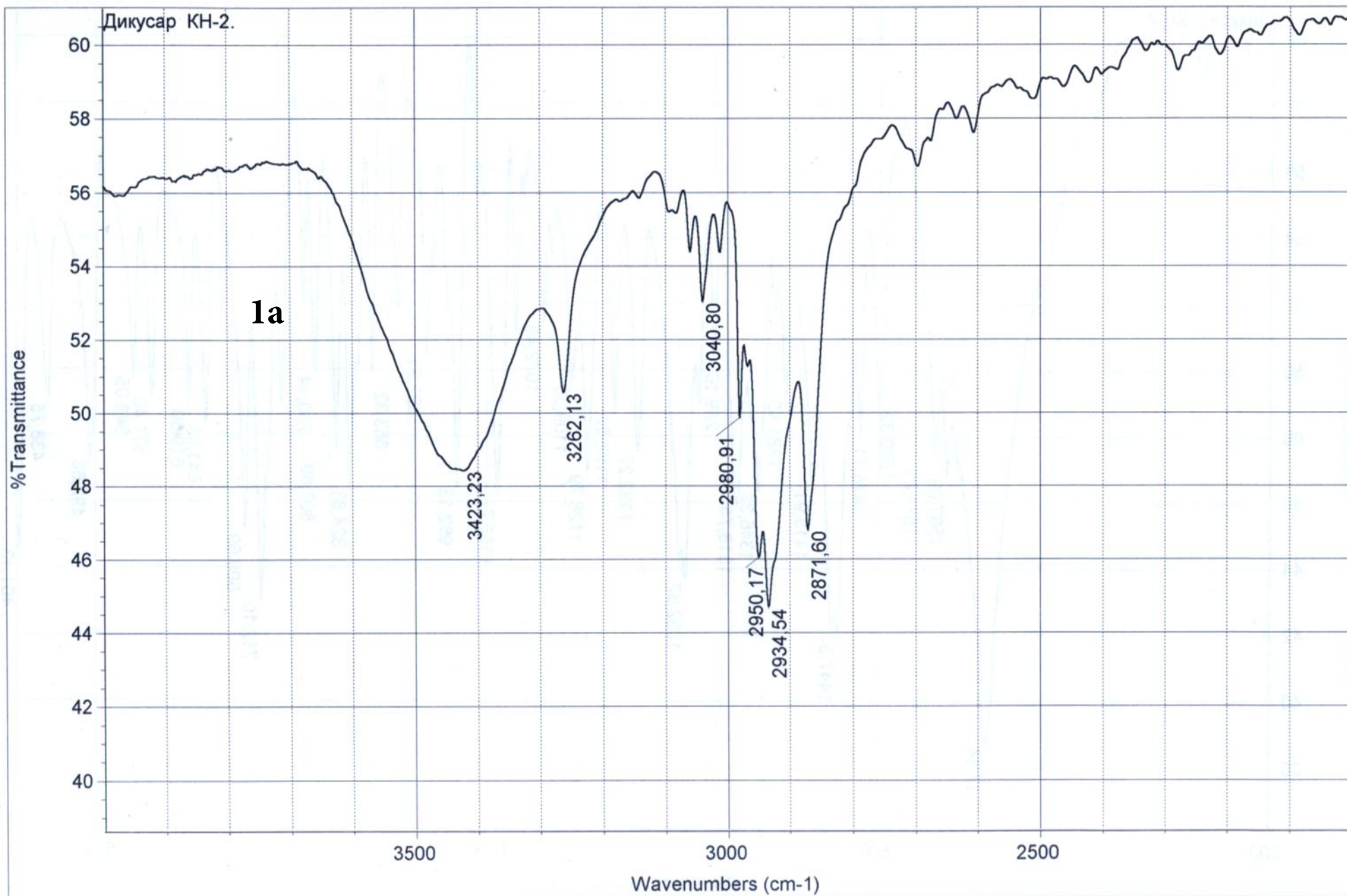

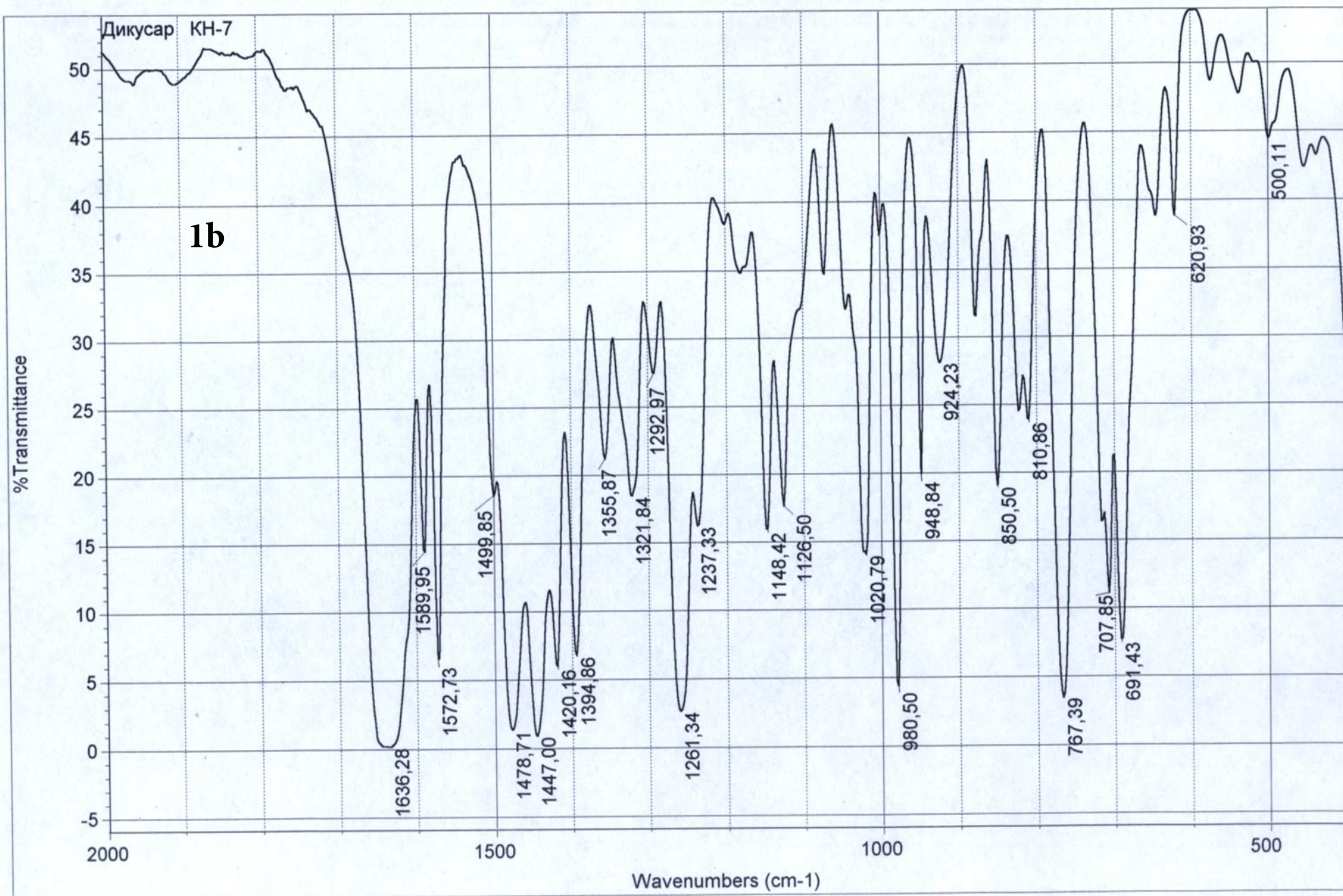

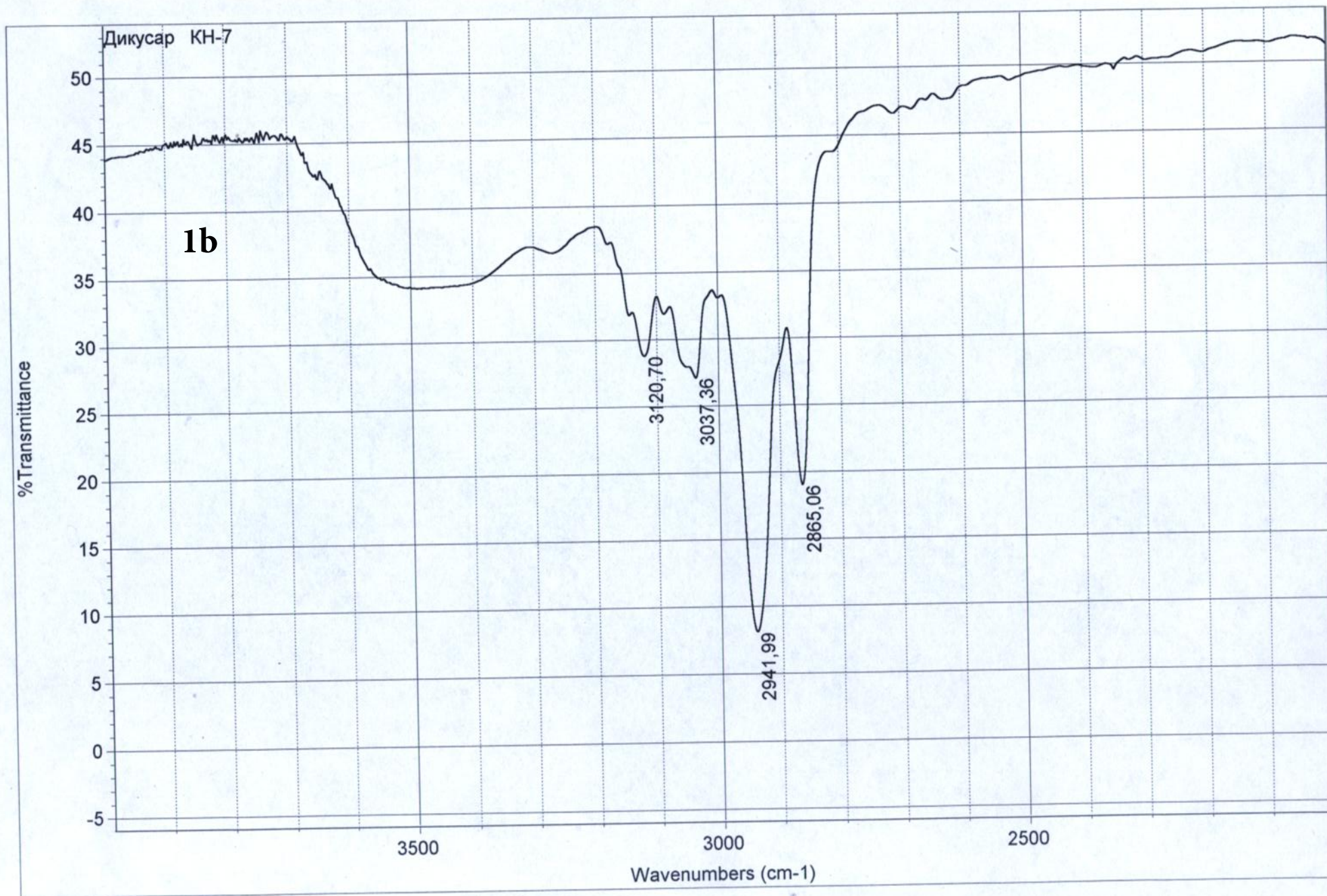

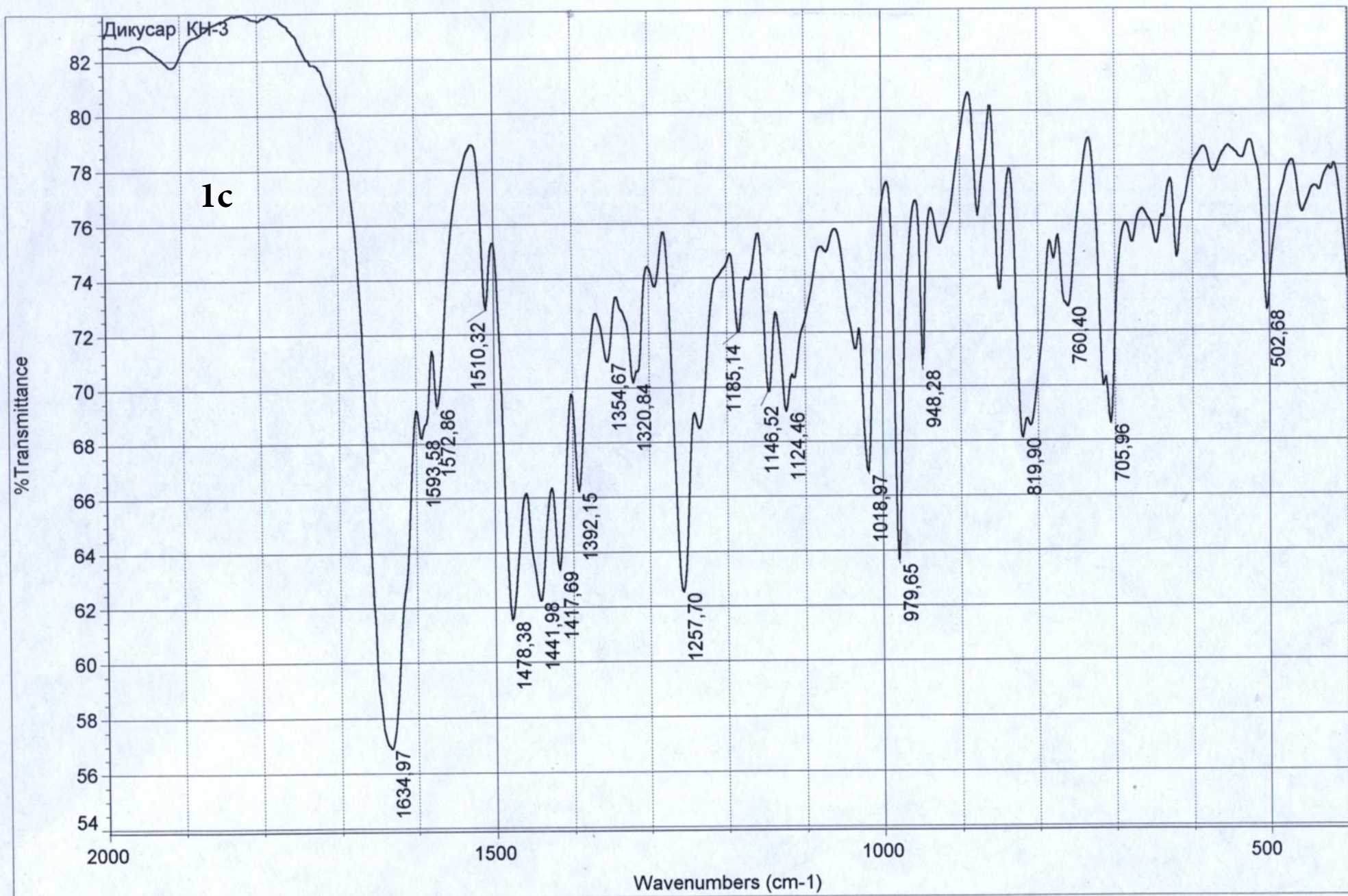

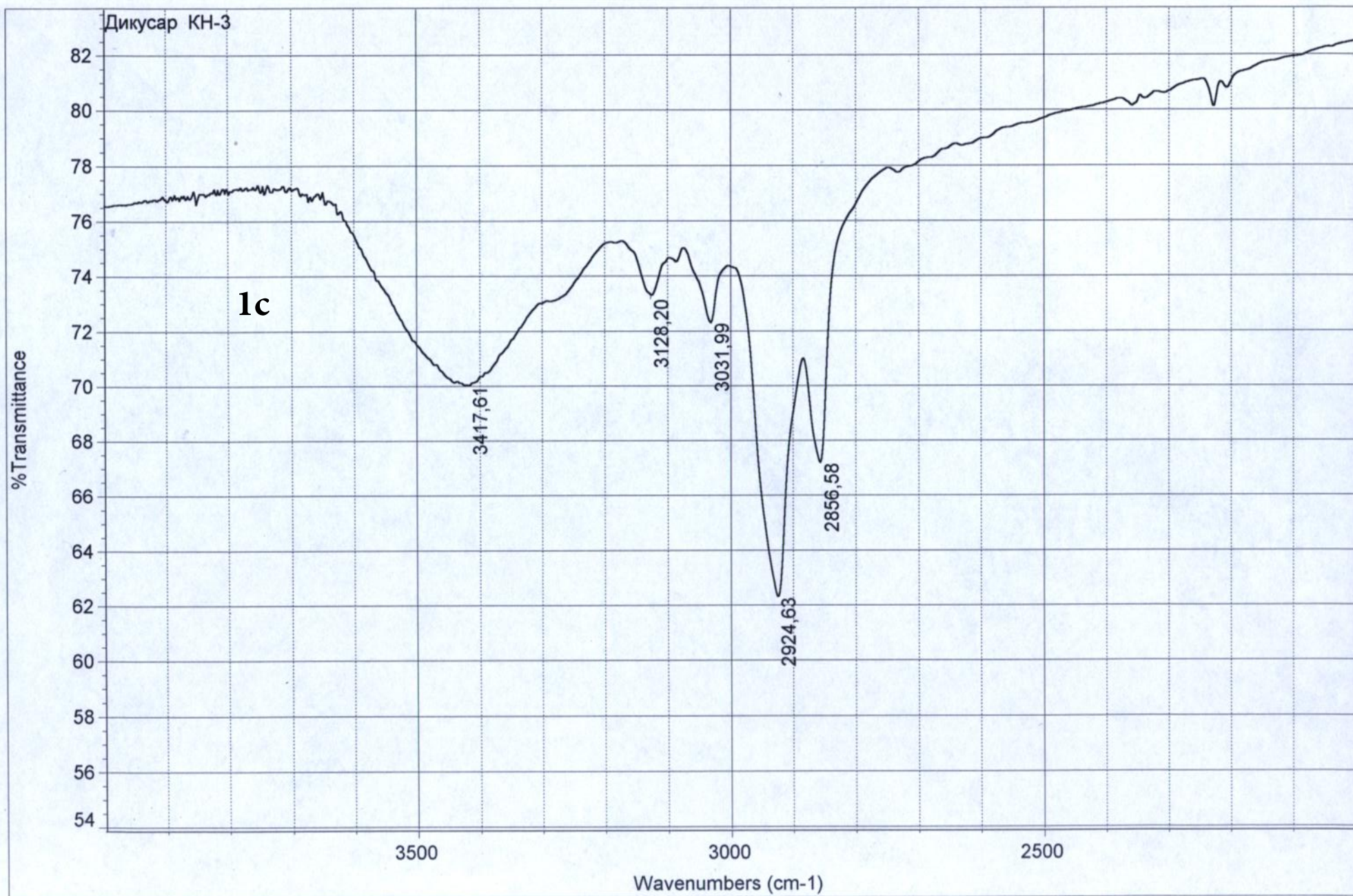

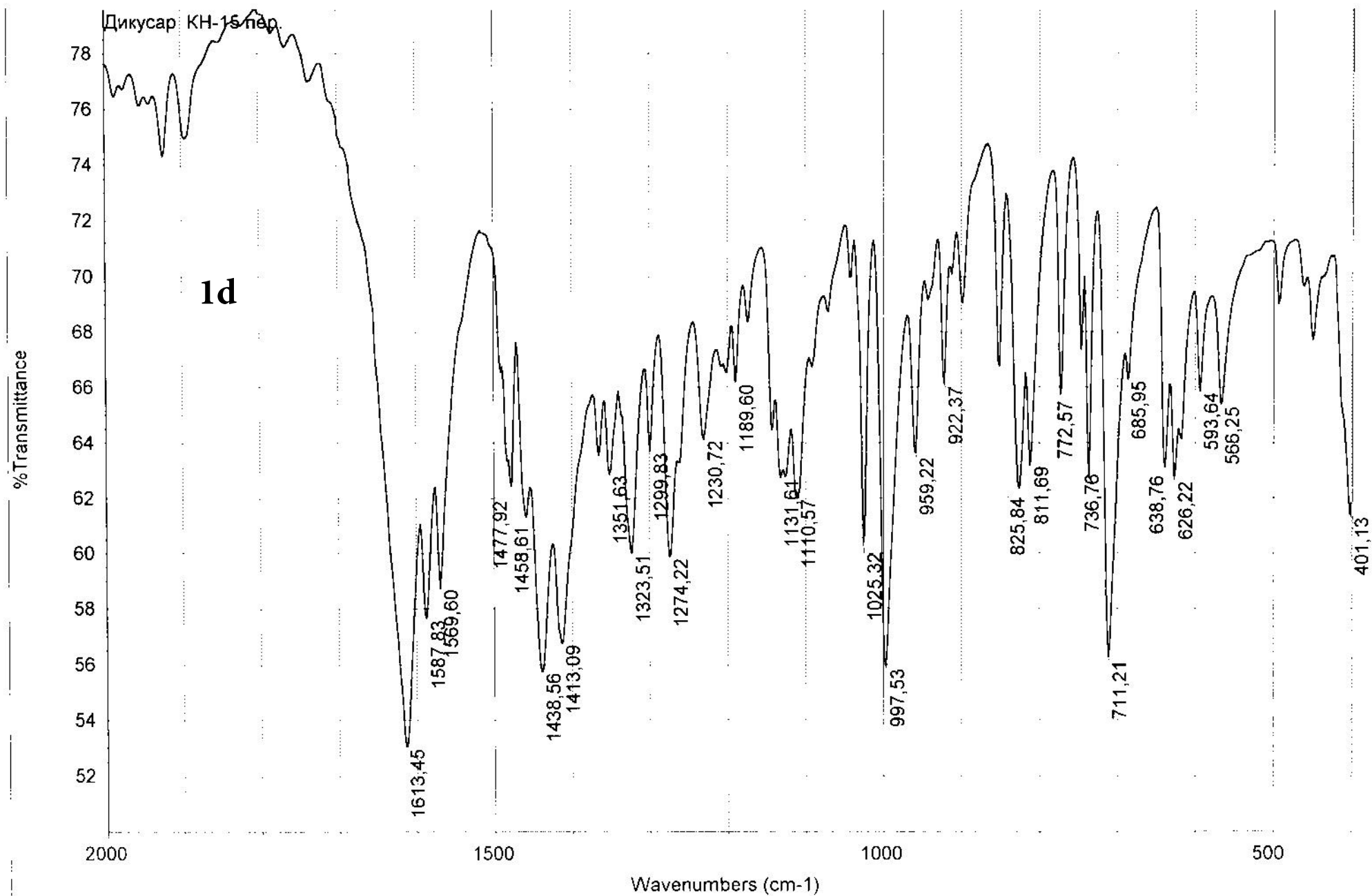

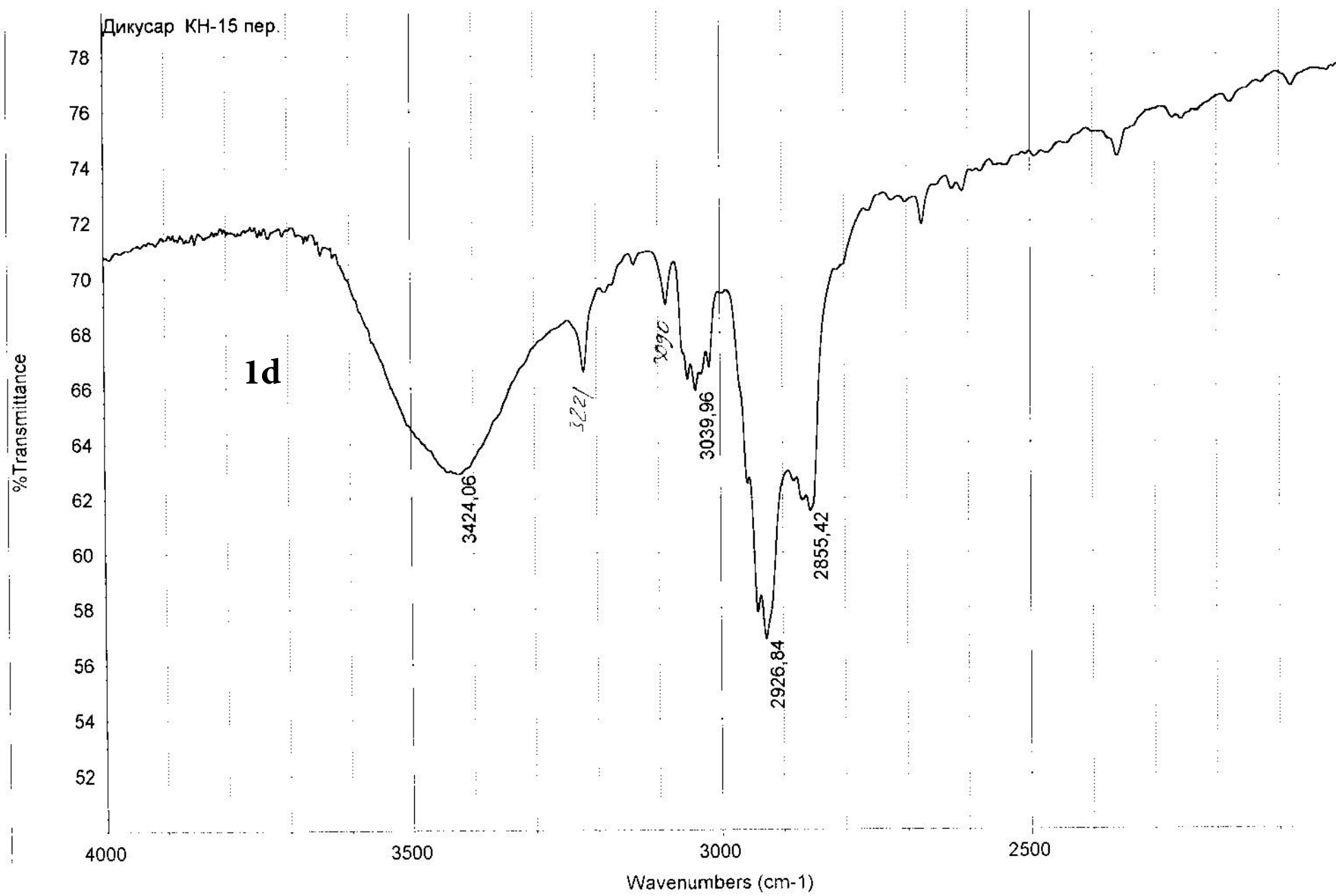

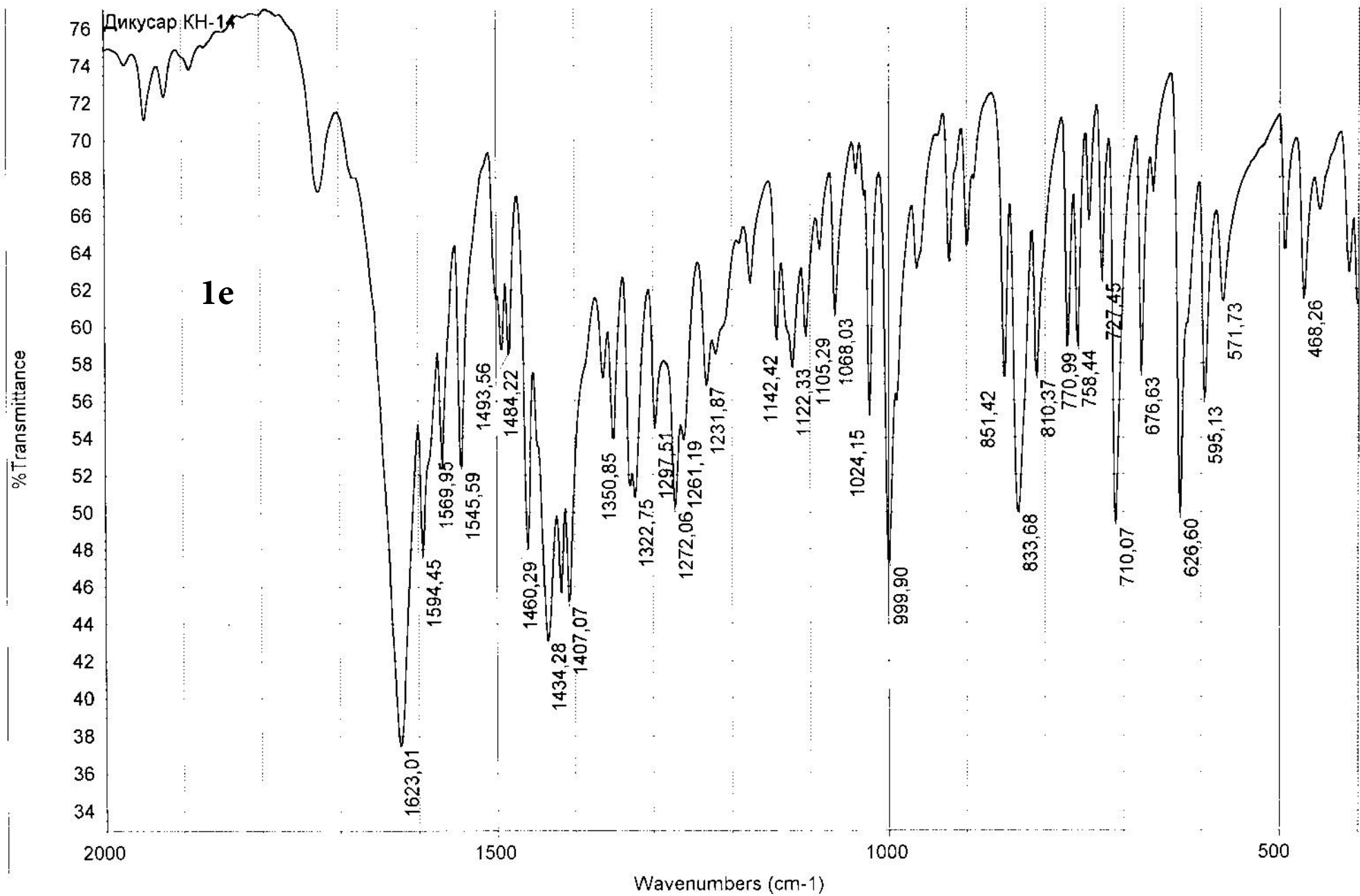

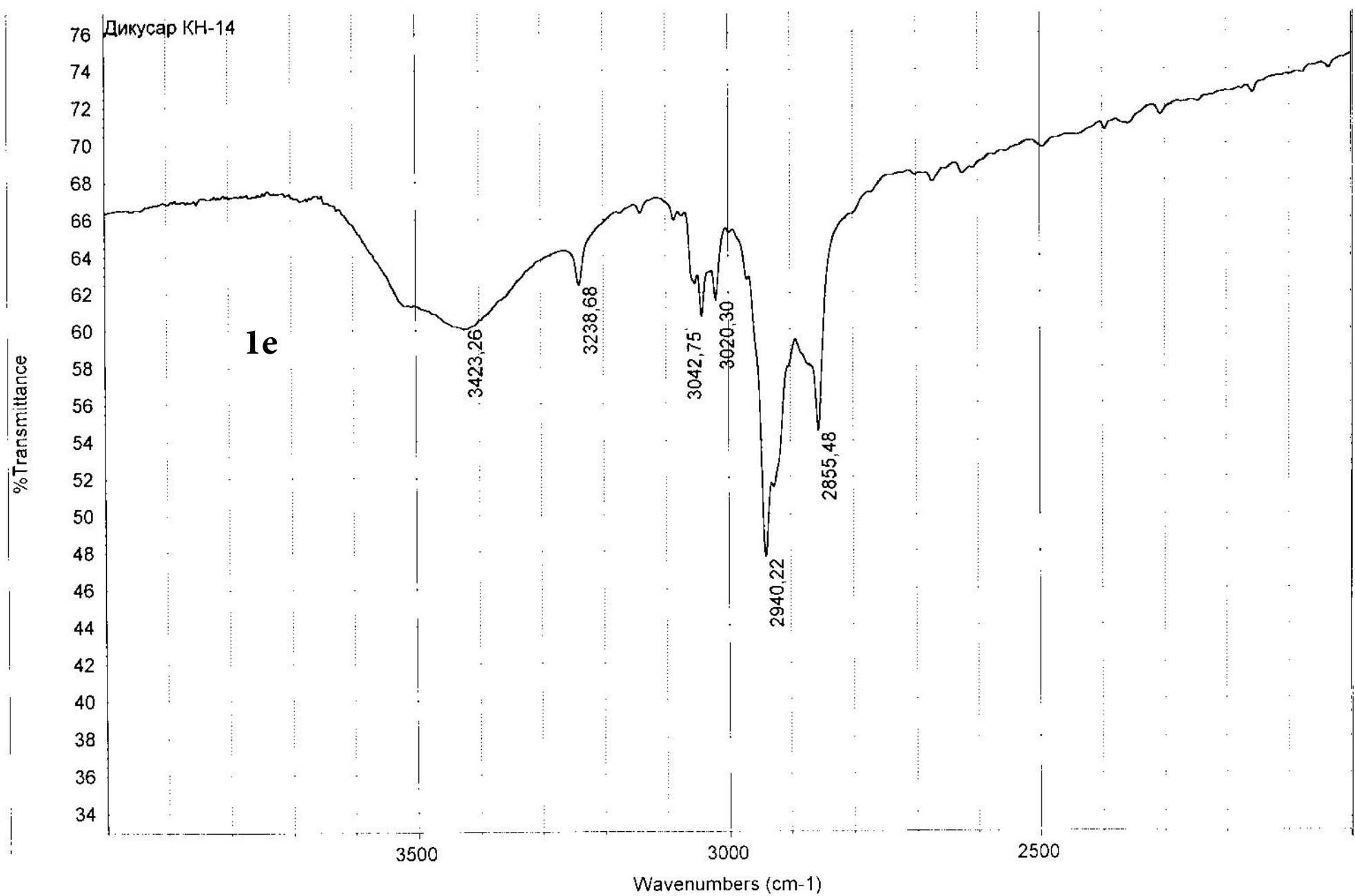

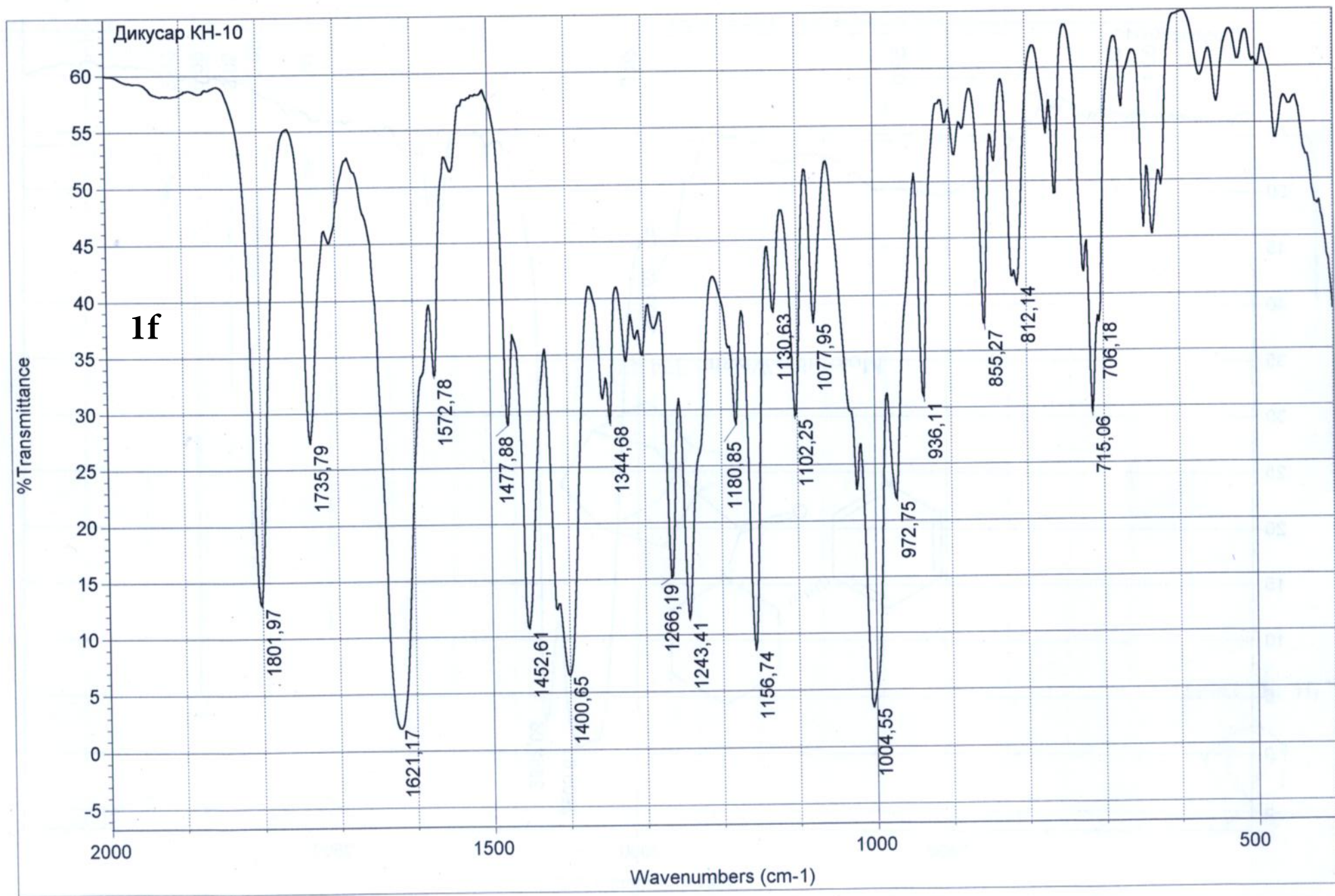

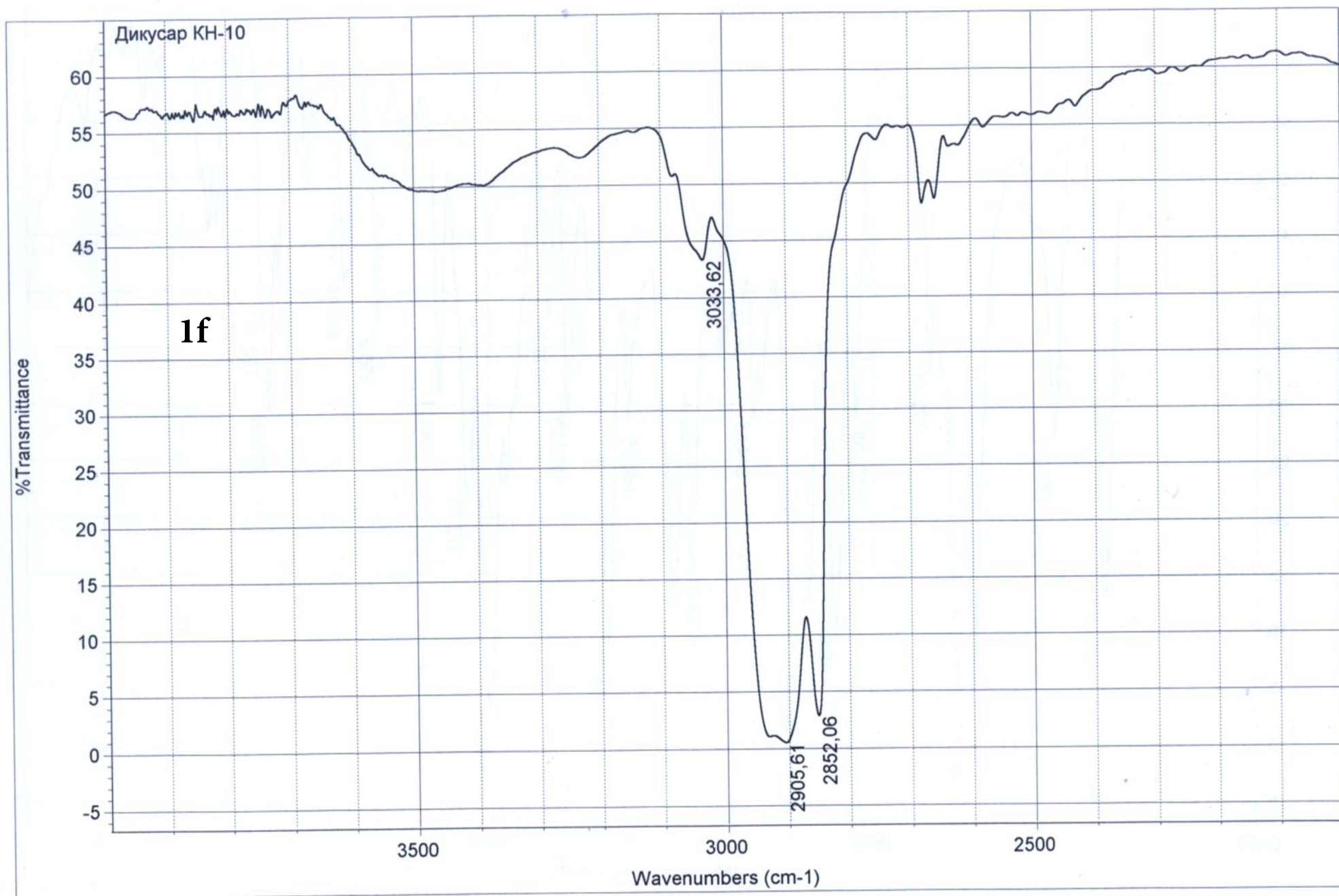

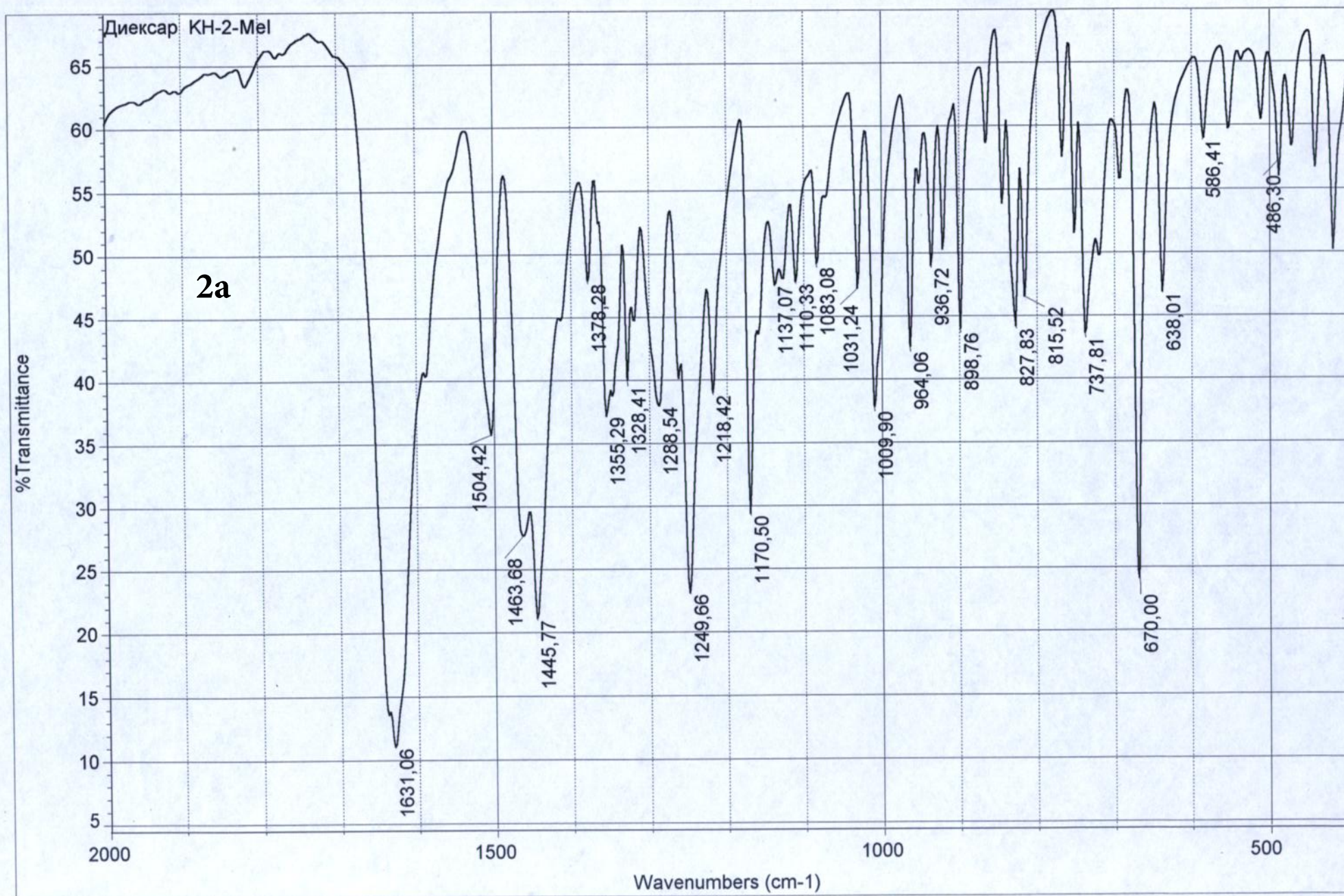

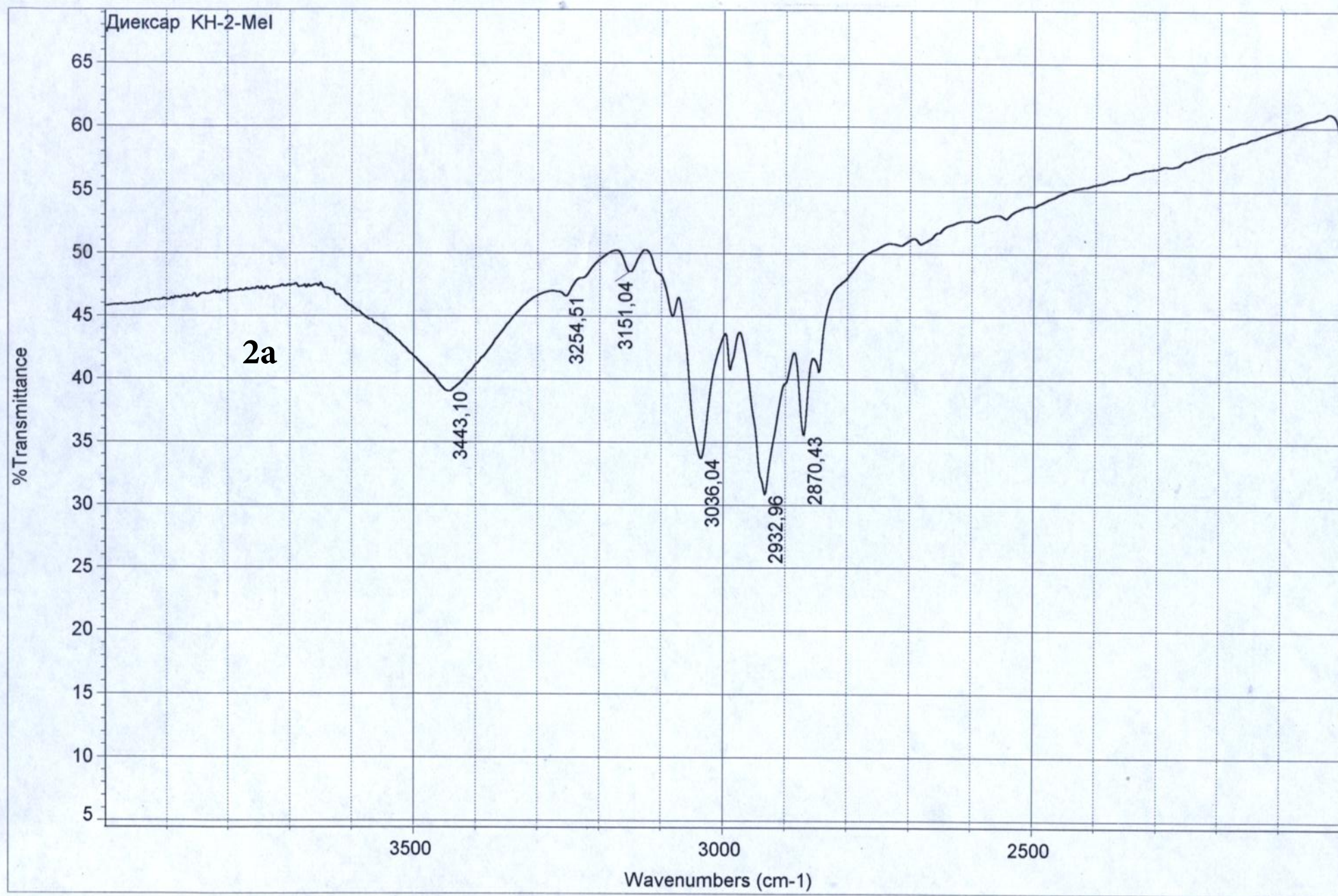

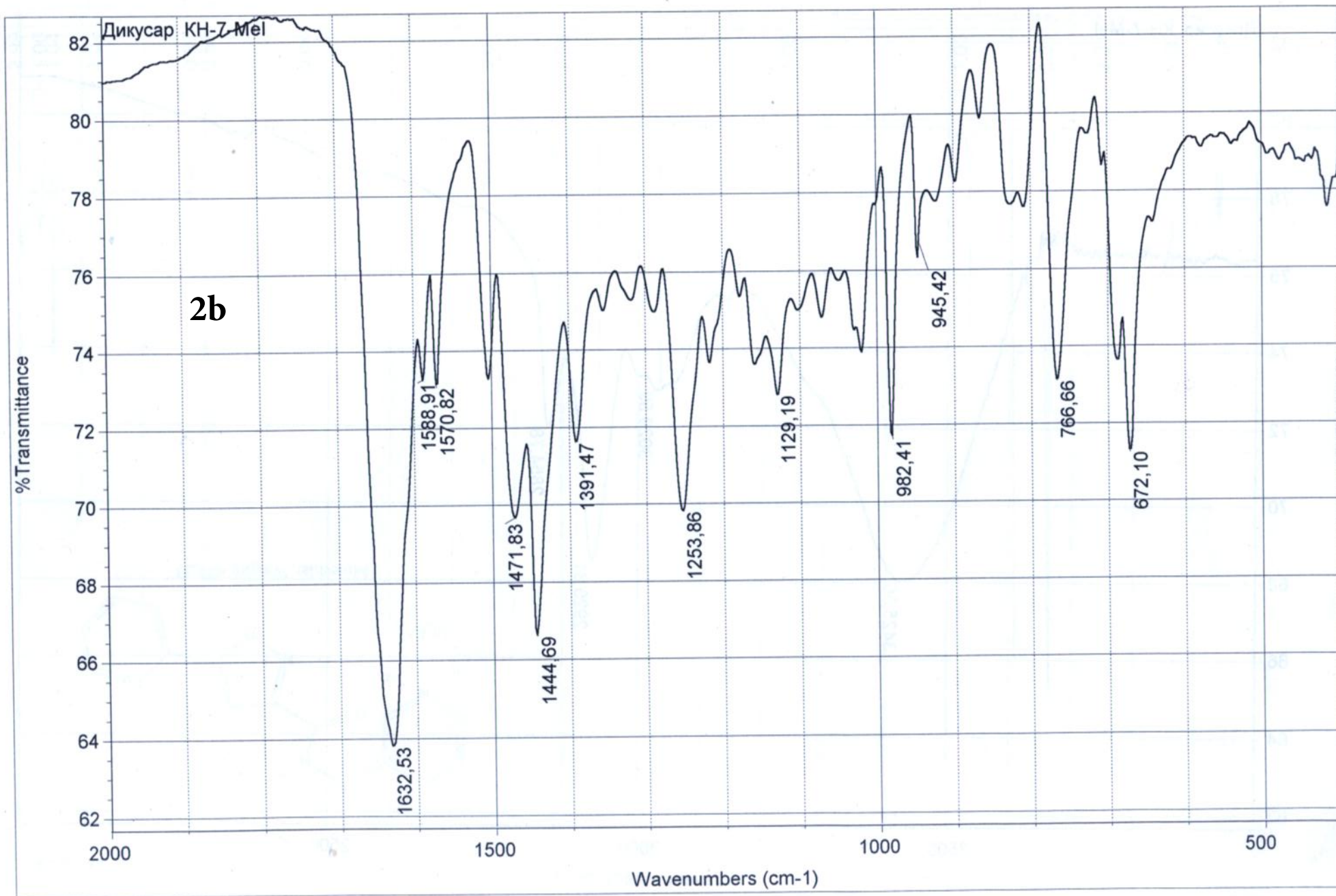

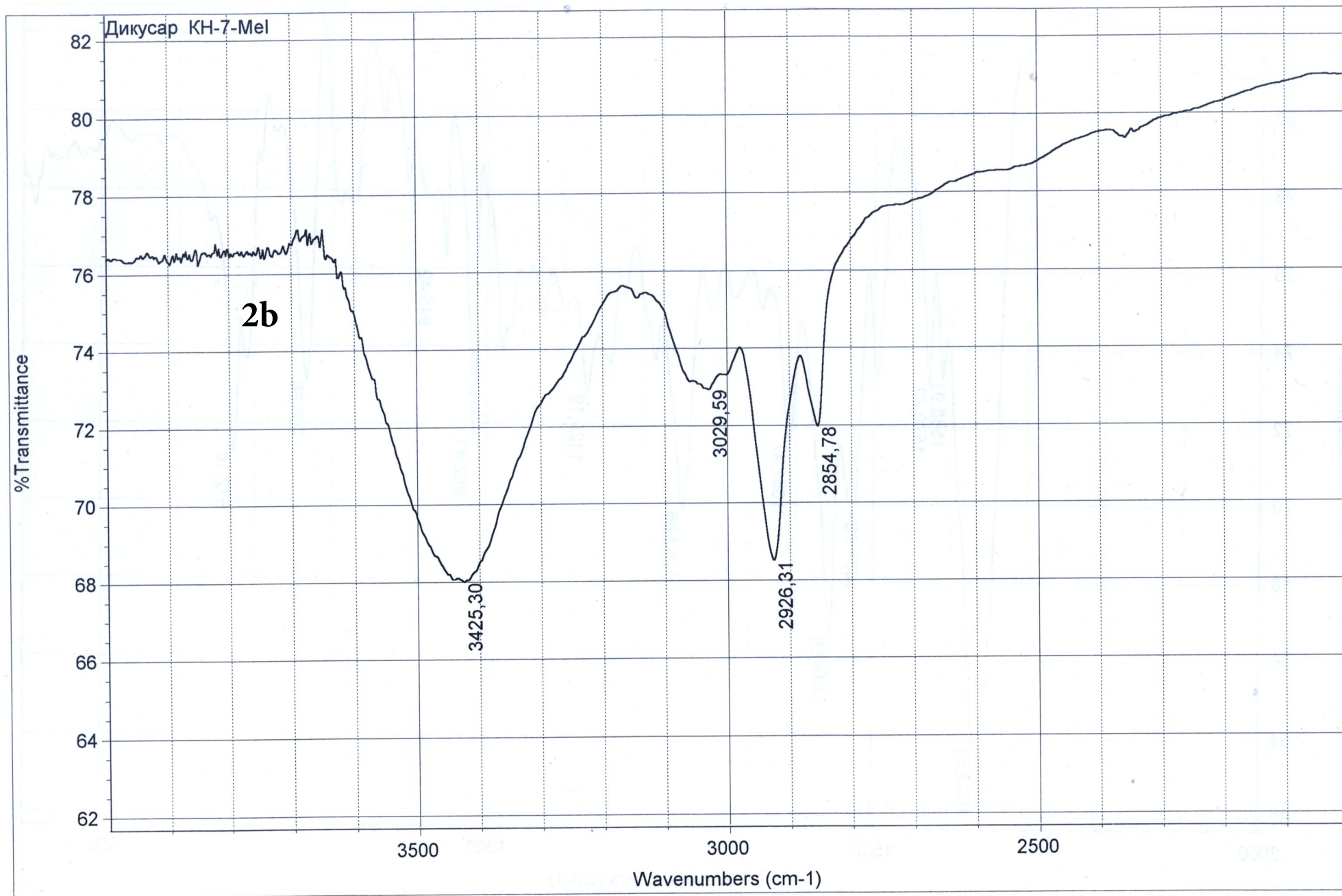

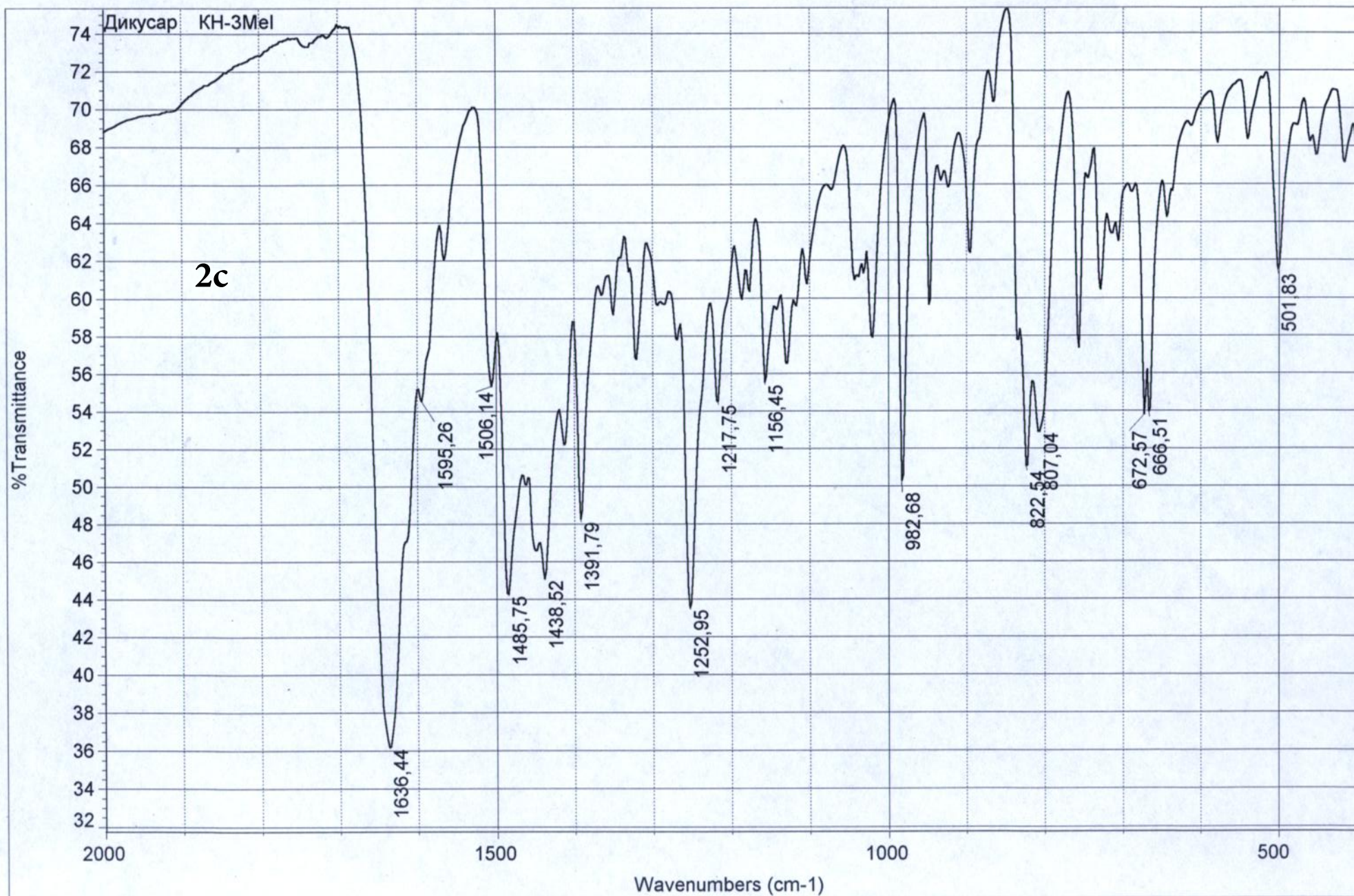

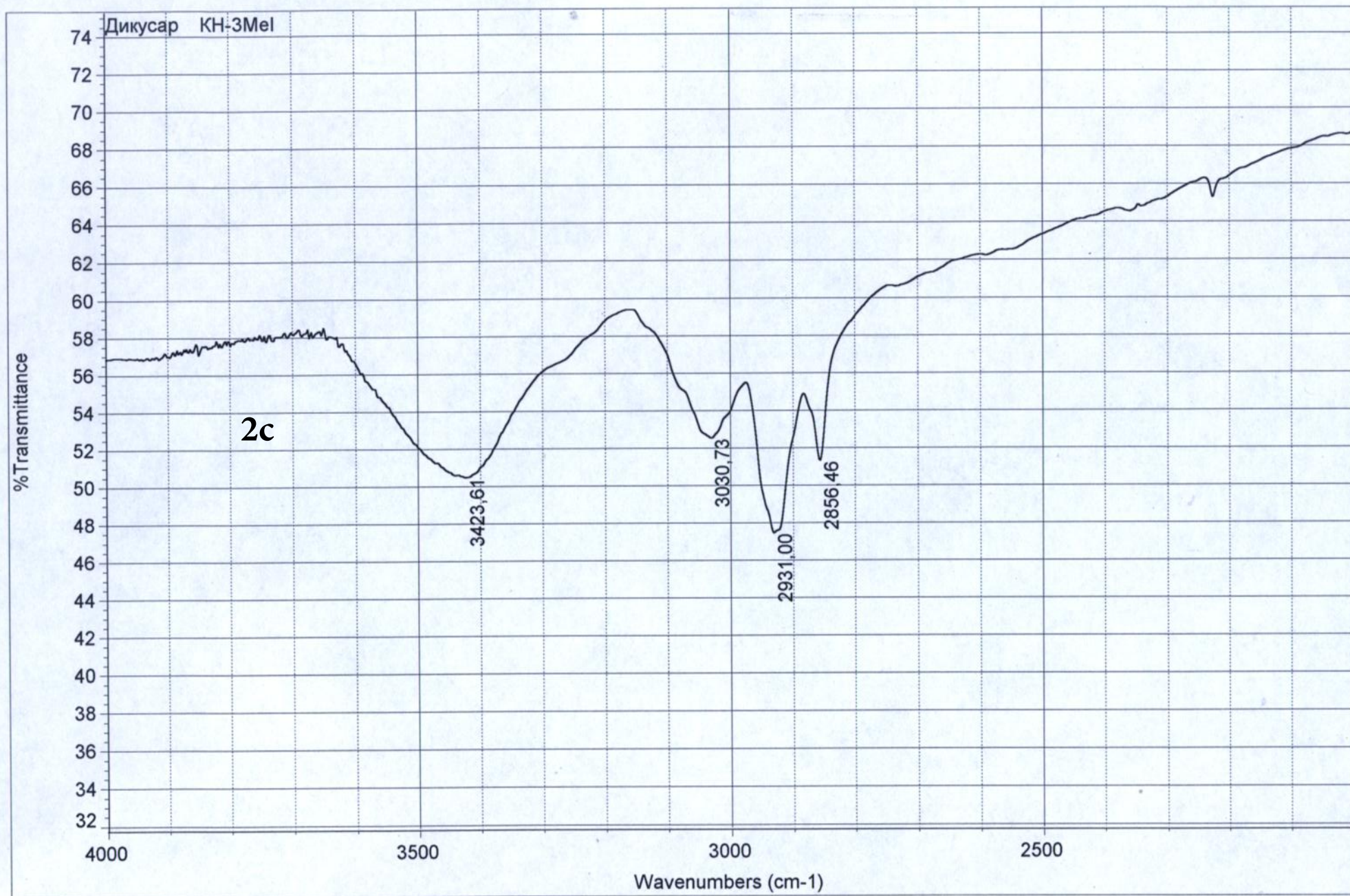

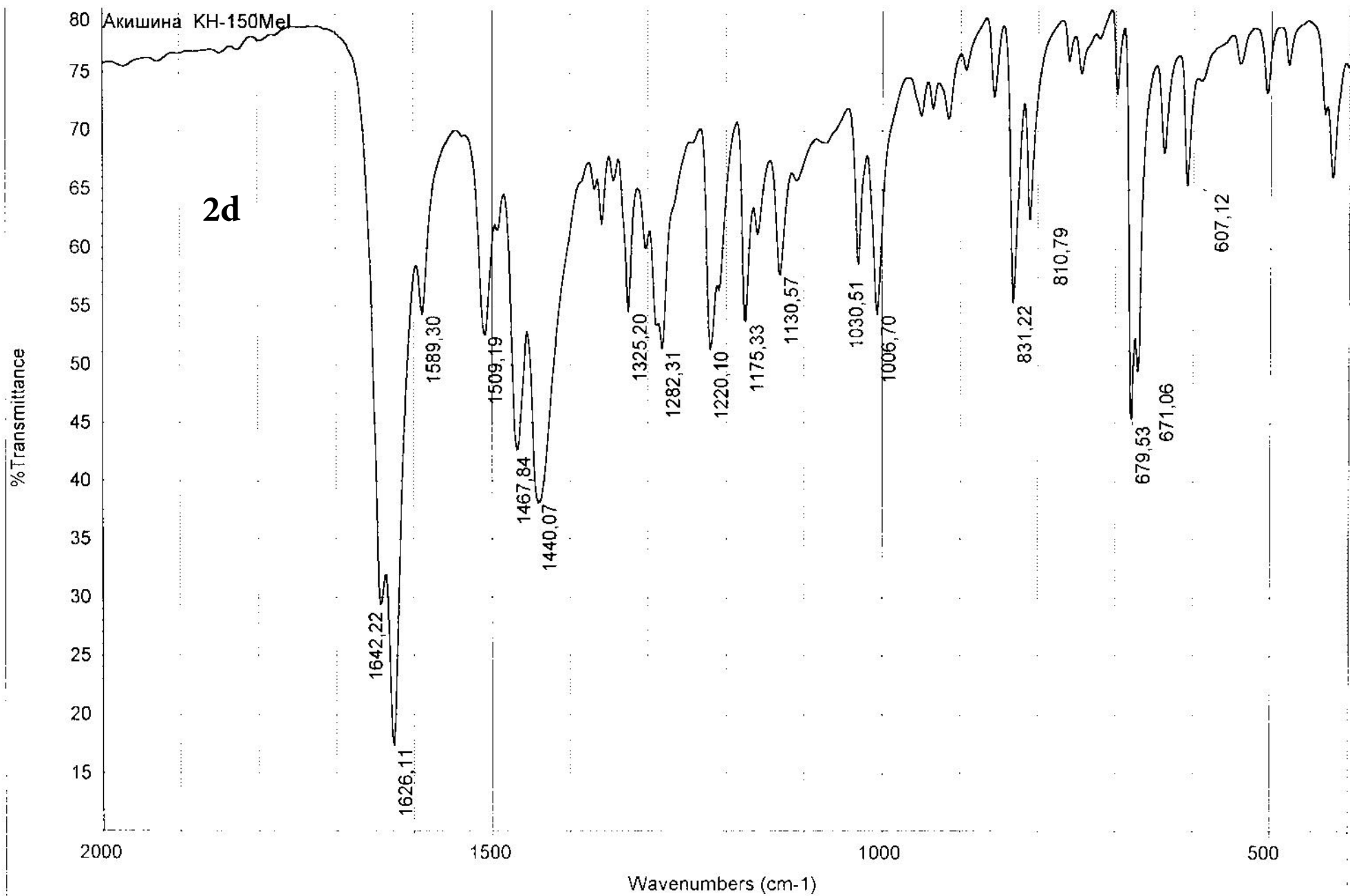

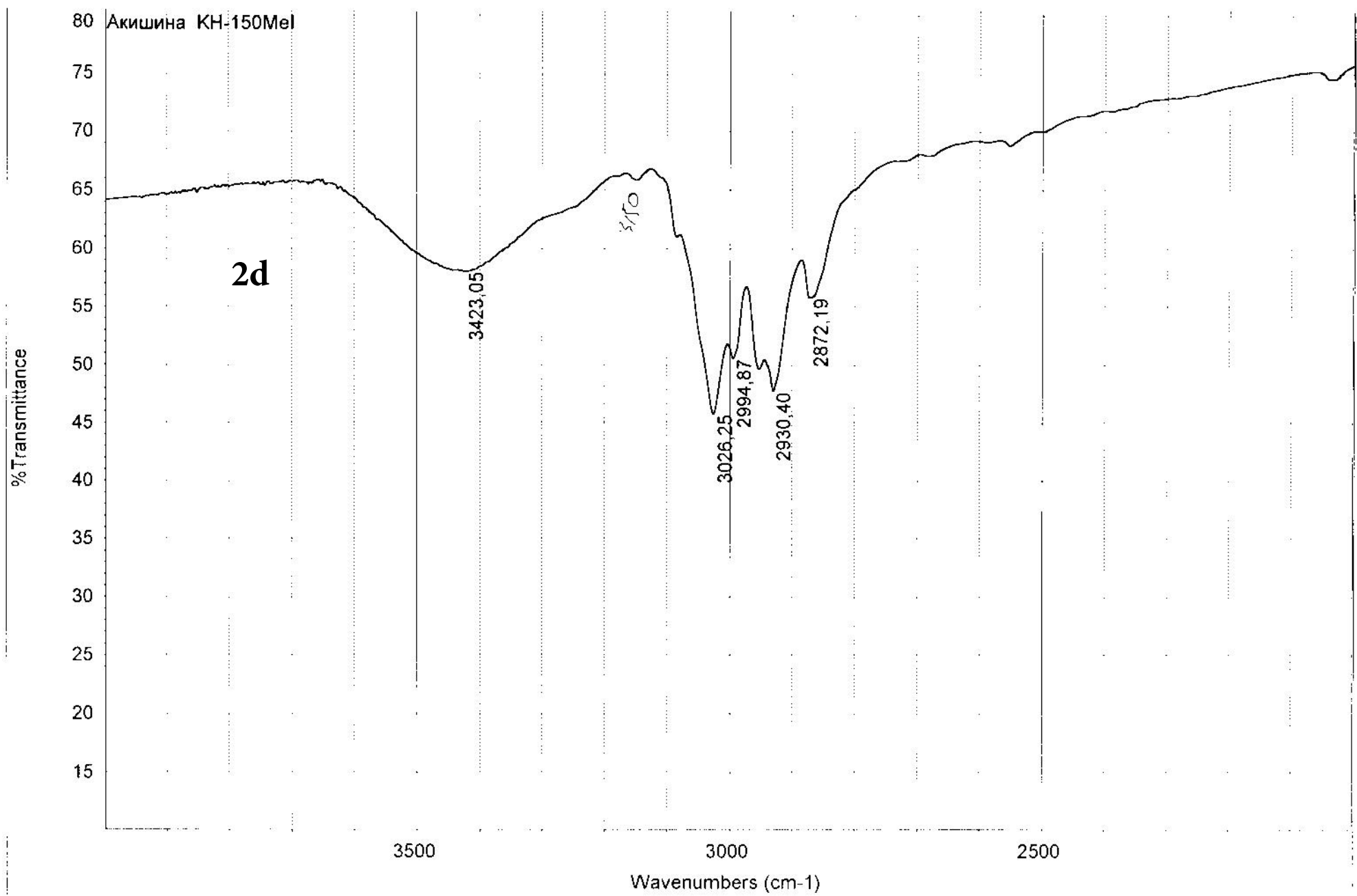

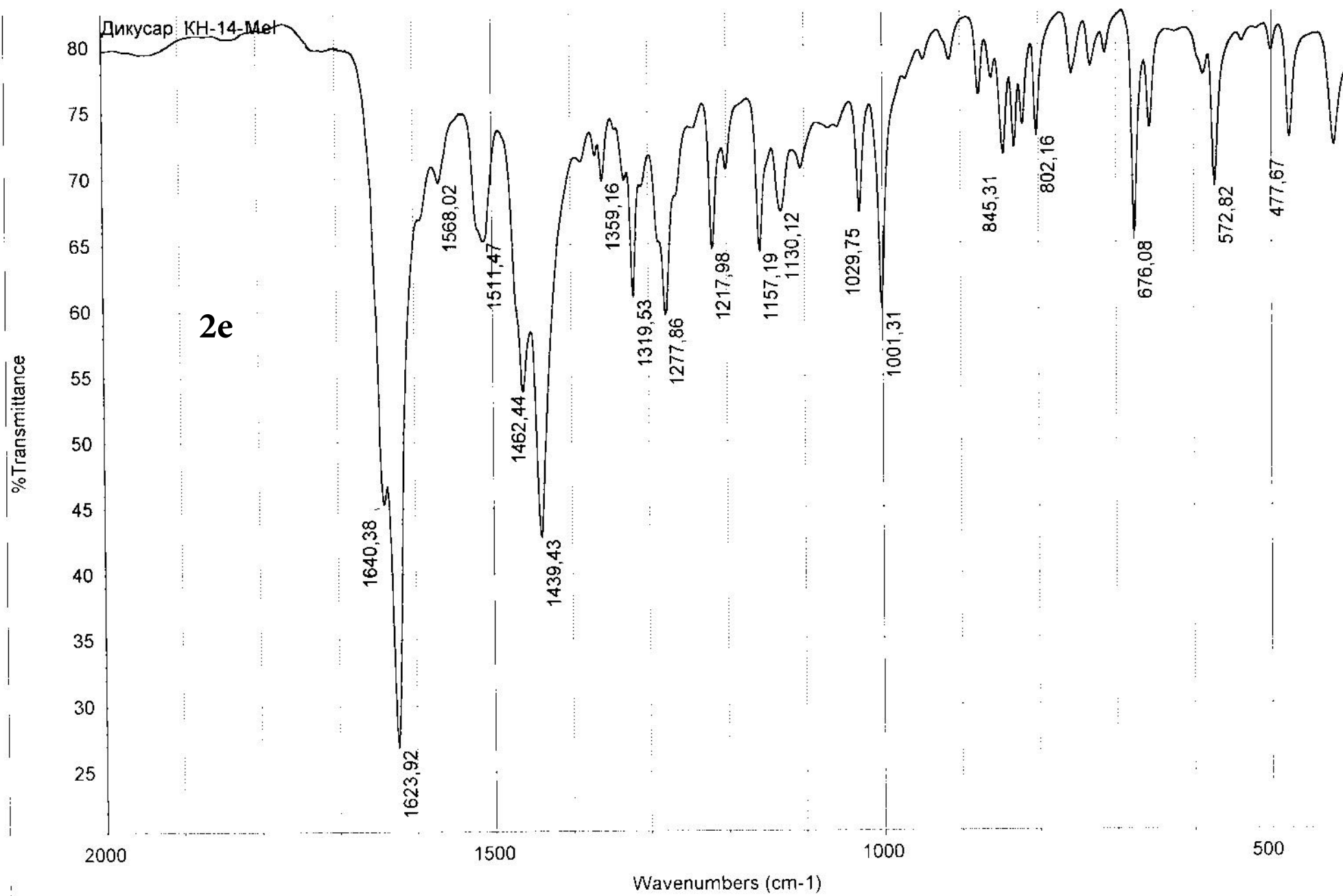

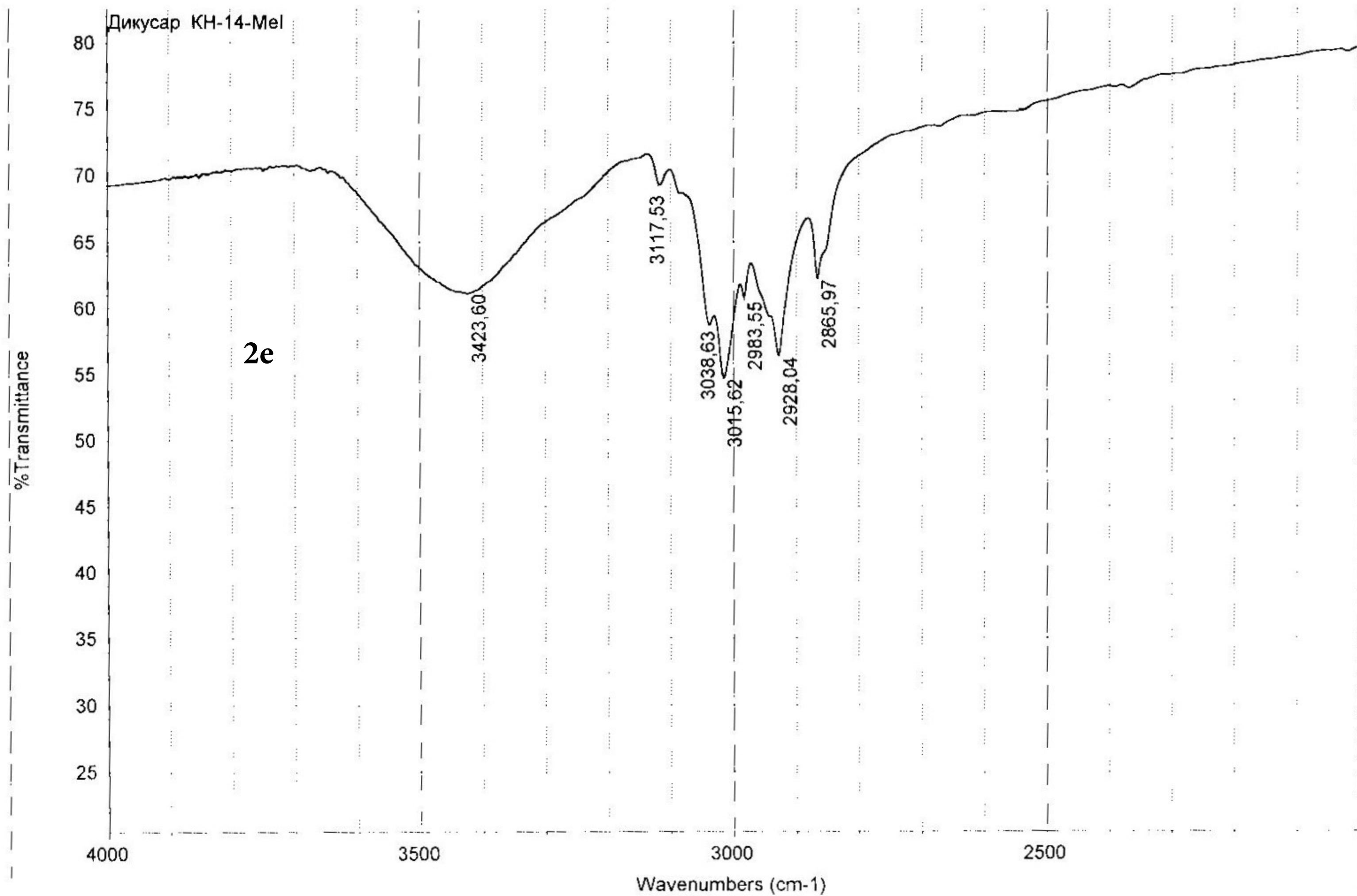

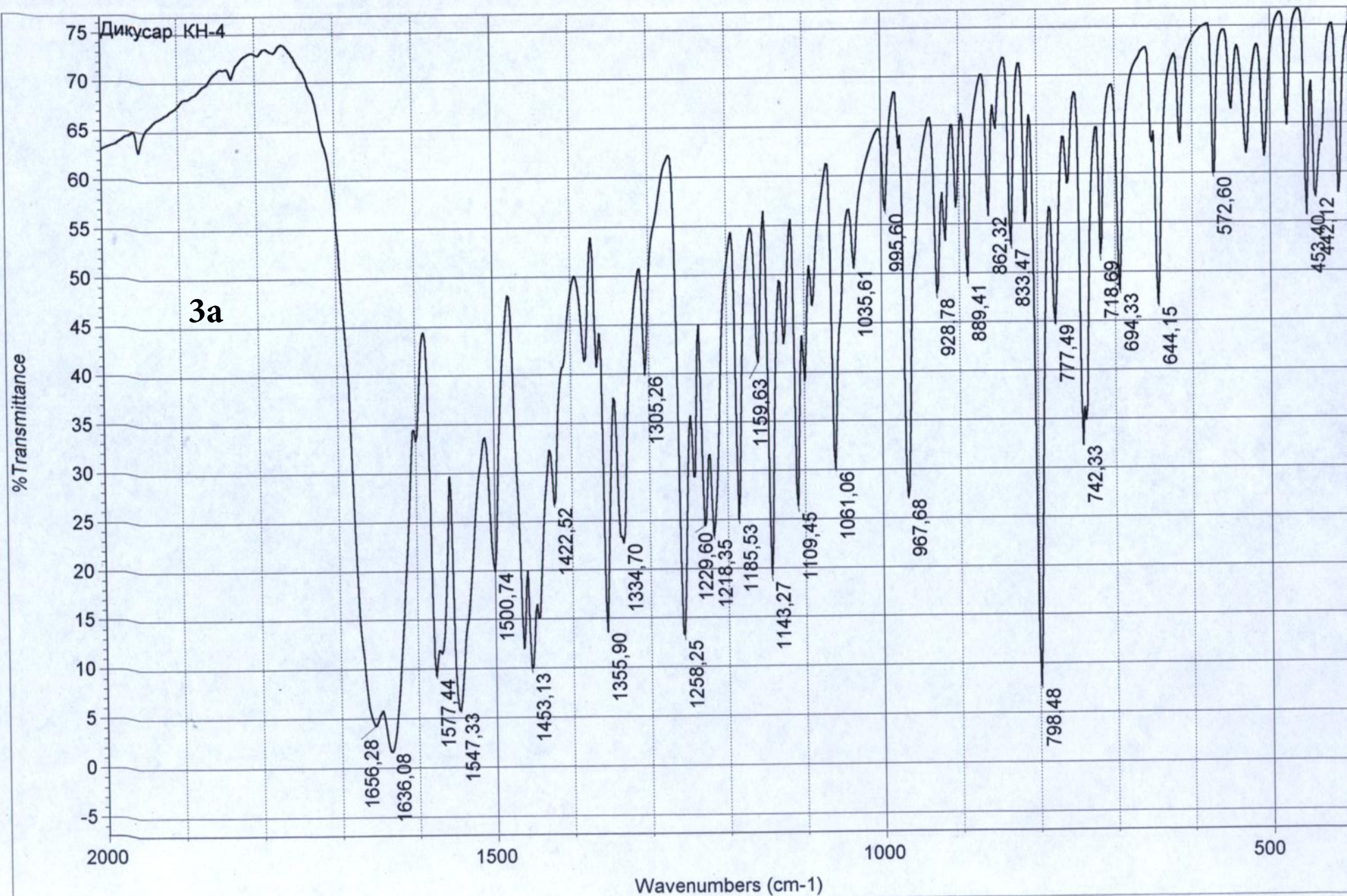

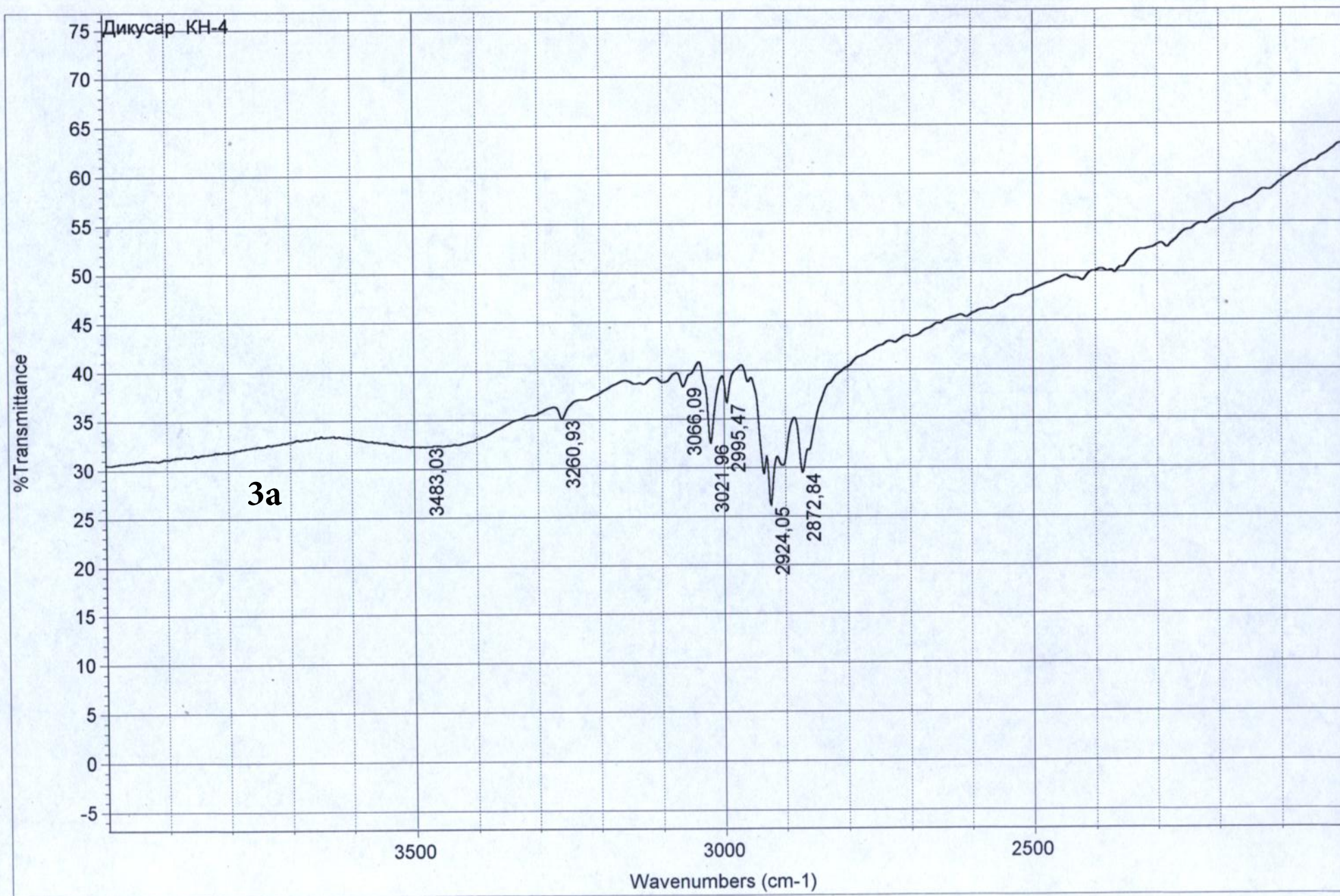

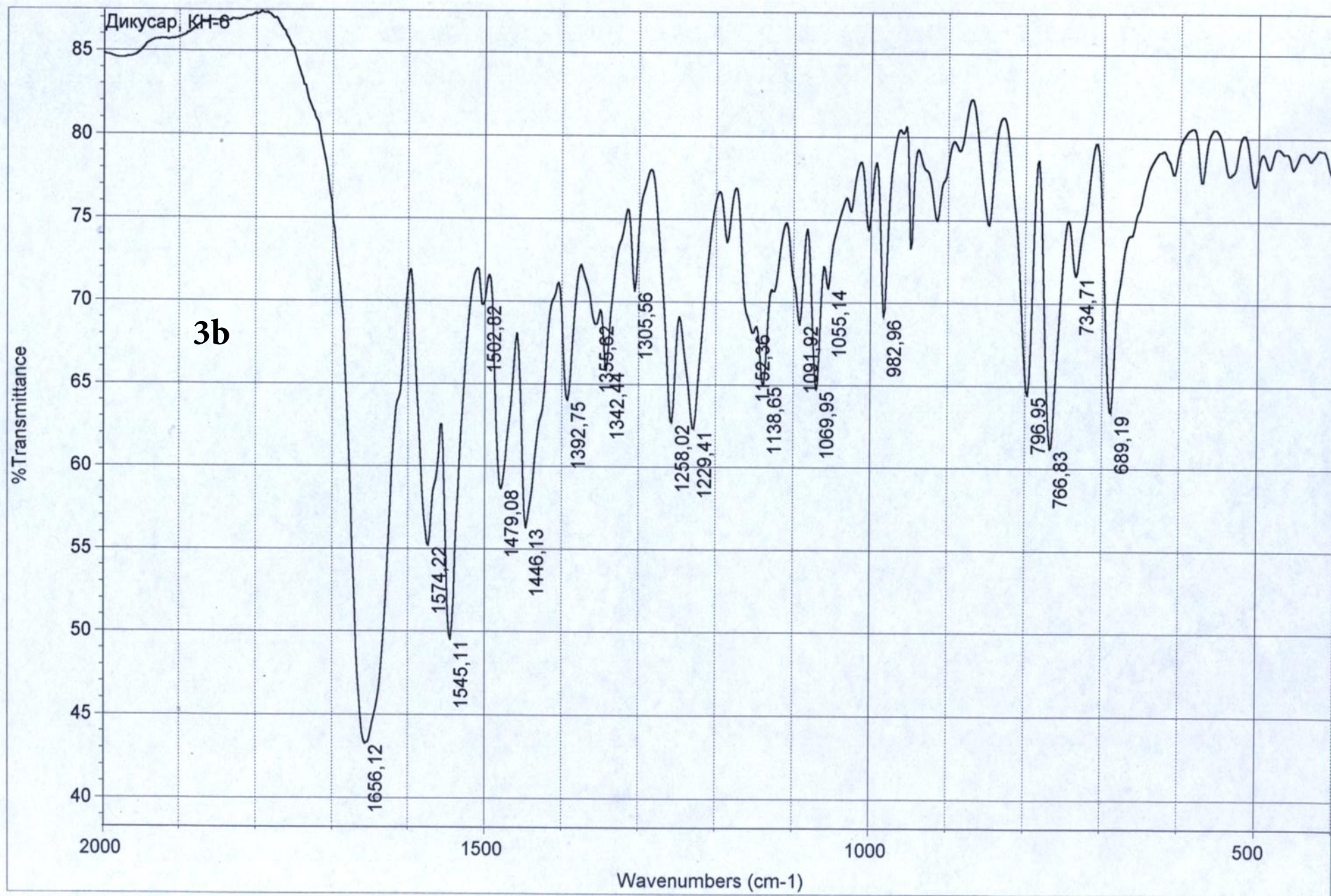

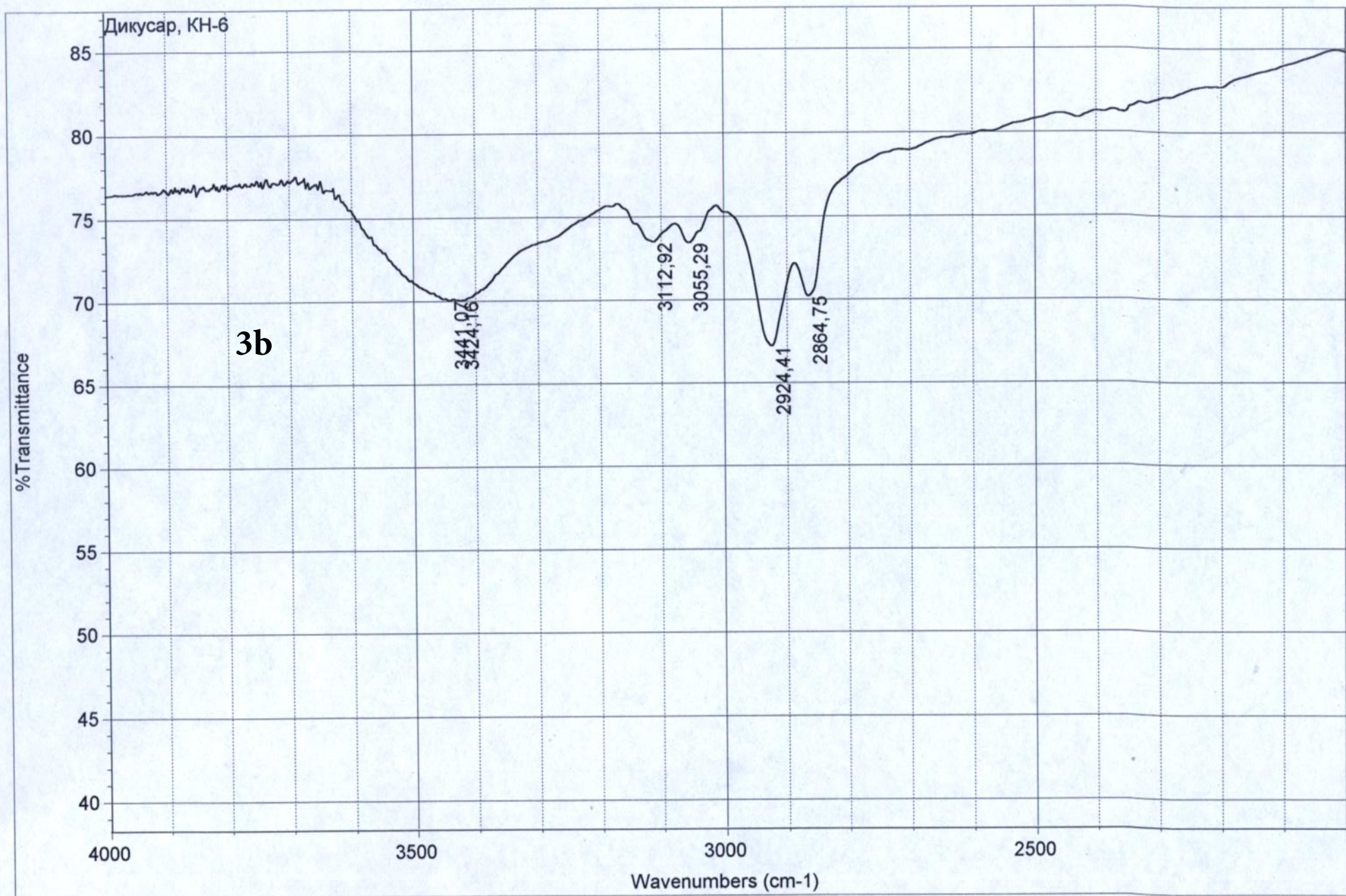

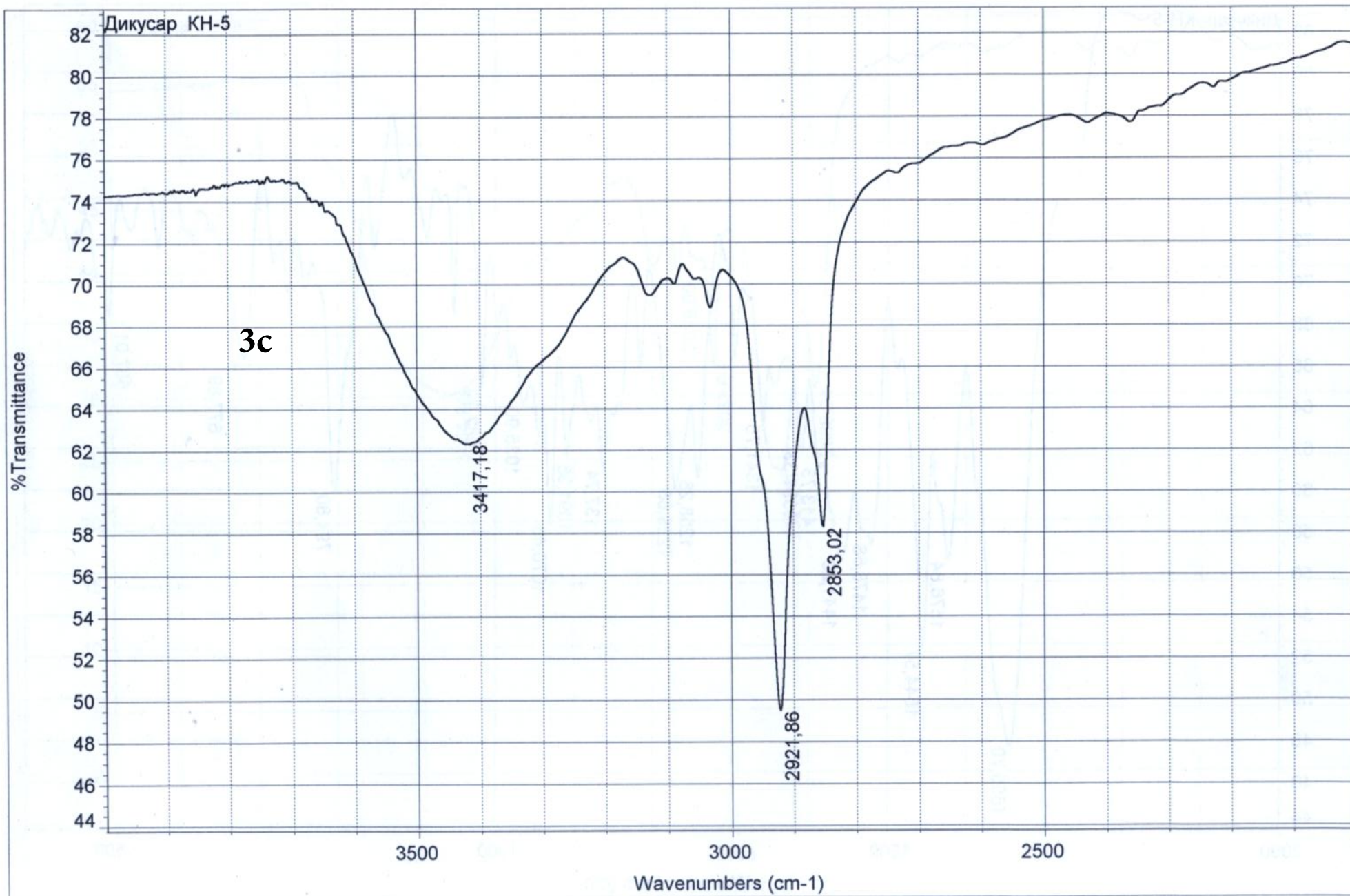

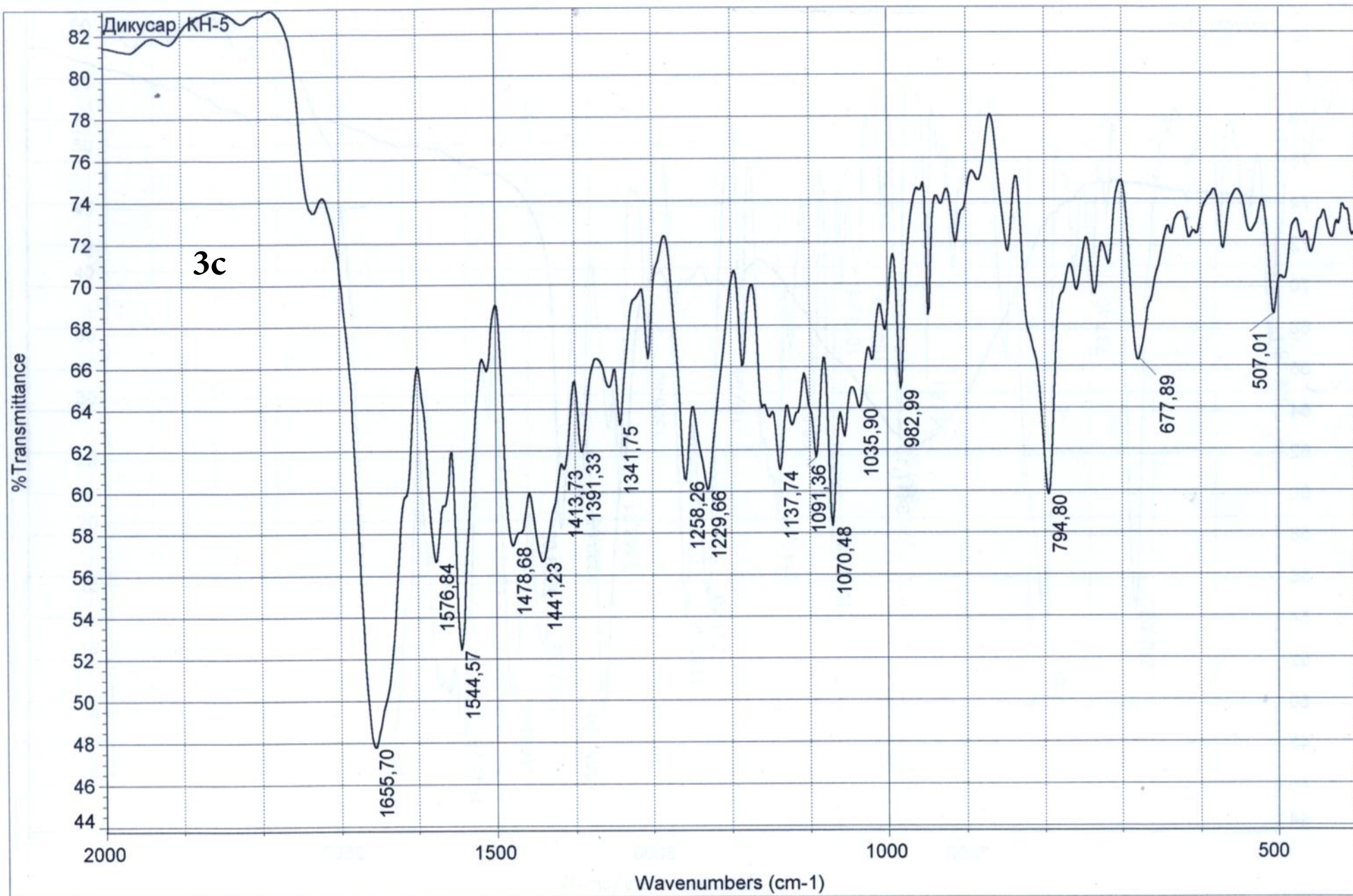

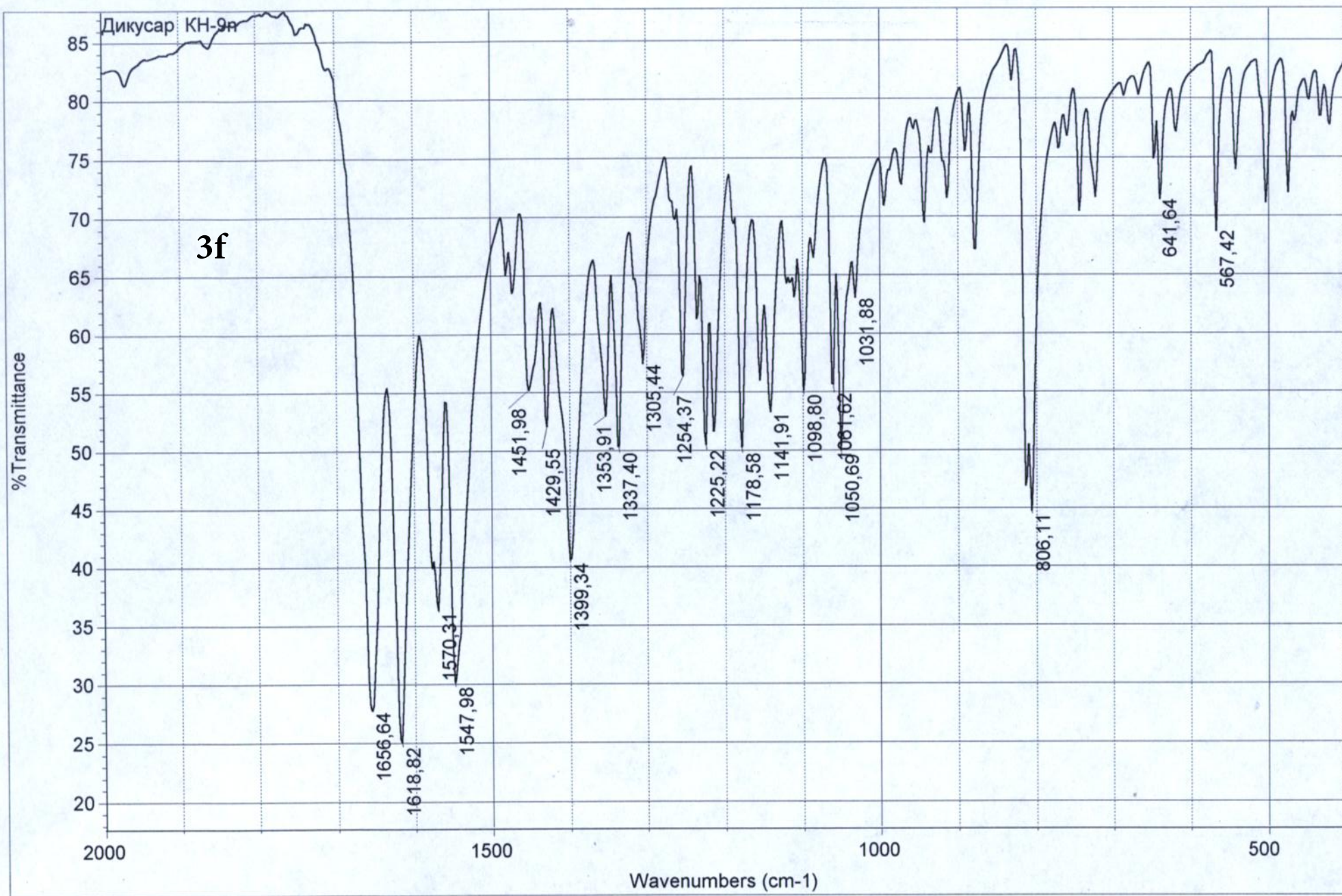

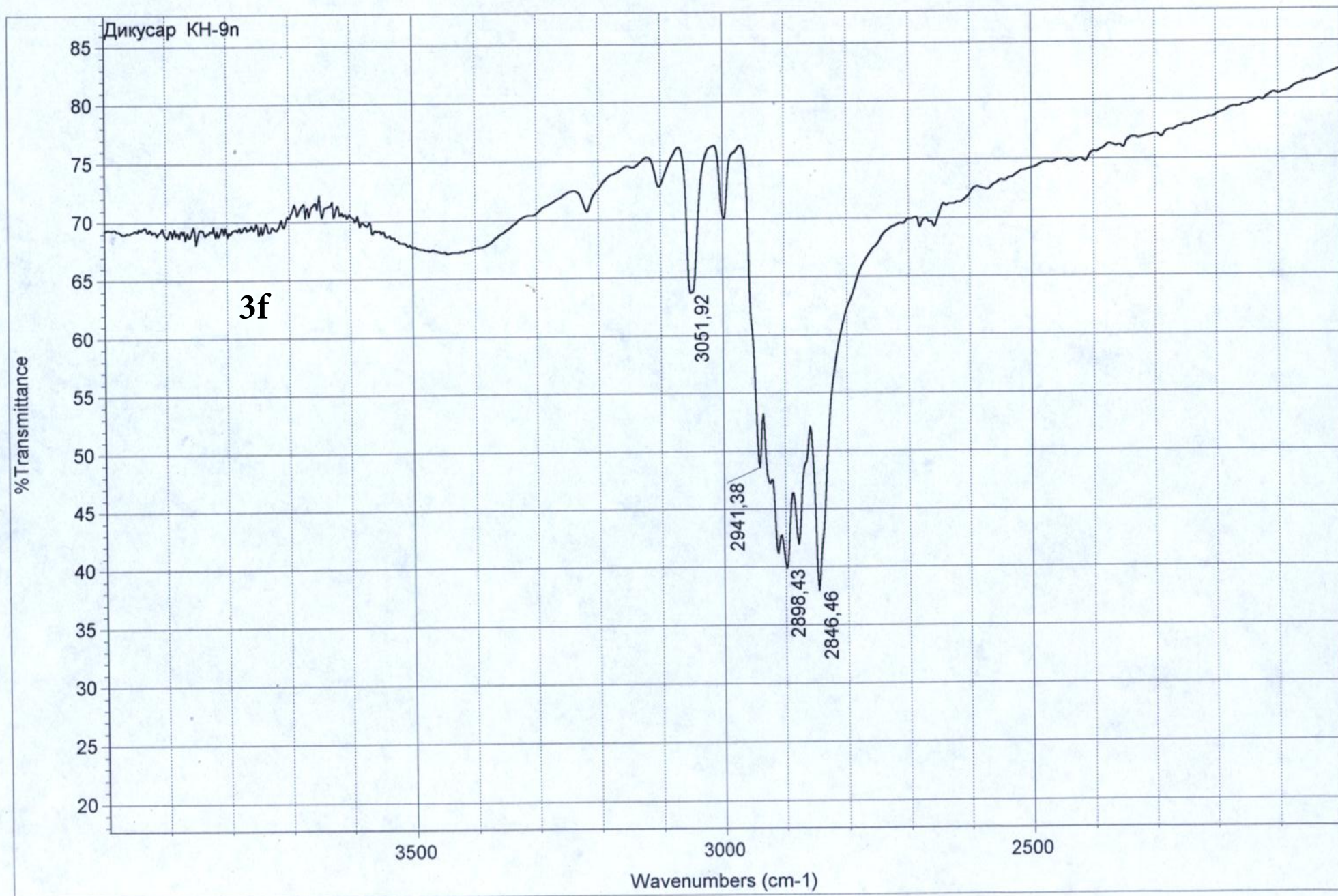

Supplement: Supplementary file 1 [file molecules-27-07387-s001.zip › IR.pdf]
